# Facile One-Pot Fischer–Suzuki–Knoevenagel Microwave-Assisted Synthesis of Fluorescent 5-Aryl-2-Styryl-3*H*-Indoles

**DOI:** 10.3390/molecules30122503

**Published:** 2025-06-07

**Authors:** Martynas Rojus Bartkus, Neringa Kleizienė, Aurimas Bieliauskas, Algirdas Šačkus

**Affiliations:** 1 Department of Organic Chemistry, Kaunas University of Technology, Radvilėnų pl. 19, LT-50254 Kaunas, Lithuania; 2 Institute of Synthetic Chemistry, Kaunas University of Technology, K. Baršausko g. 59, LT-51423 Kaunas, Lithuania; neringa.kleiziene@ktu.lt (N.K.); aurimas.bieliauskas@ktu.lt (A.B.)

**Keywords:** one-pot synthesis, consecutive multicomponent reaction, microwave synthesis, Fischer synthesis, Suzuki cross-coupling reaction, Knoevenagel condensation, 2-styryl-3*H*-indole, fluorescence

## Abstract

In this study, novel fluorescent 5-aryl-2-styryl-3*H*-indole derivatives were efficiently synthesized from 4-bromophenylhydrazine hydrochloride using the microwave-accelerated one-pot technique, which includes Fischer synthesis, Suzuki cross-coupling, and Knoevenagel condensation. The structural assignments of the synthesized compounds were based on ^1^H, ^13^C, ^15^N, and ^19^F NMR; IR spectroscopy; and HRMS spectral data. The optical properties of the newly obtained styryl-indole dyes were studied using UV-vis and fluorescence spectroscopy, which clearly demonstrated that the derivatives substituted with electron-donating or -withdrawing groups exhibited varying emission shifts and quantum yields ranging from negligible to high.

## 1. Introduction

**Figure 1:**
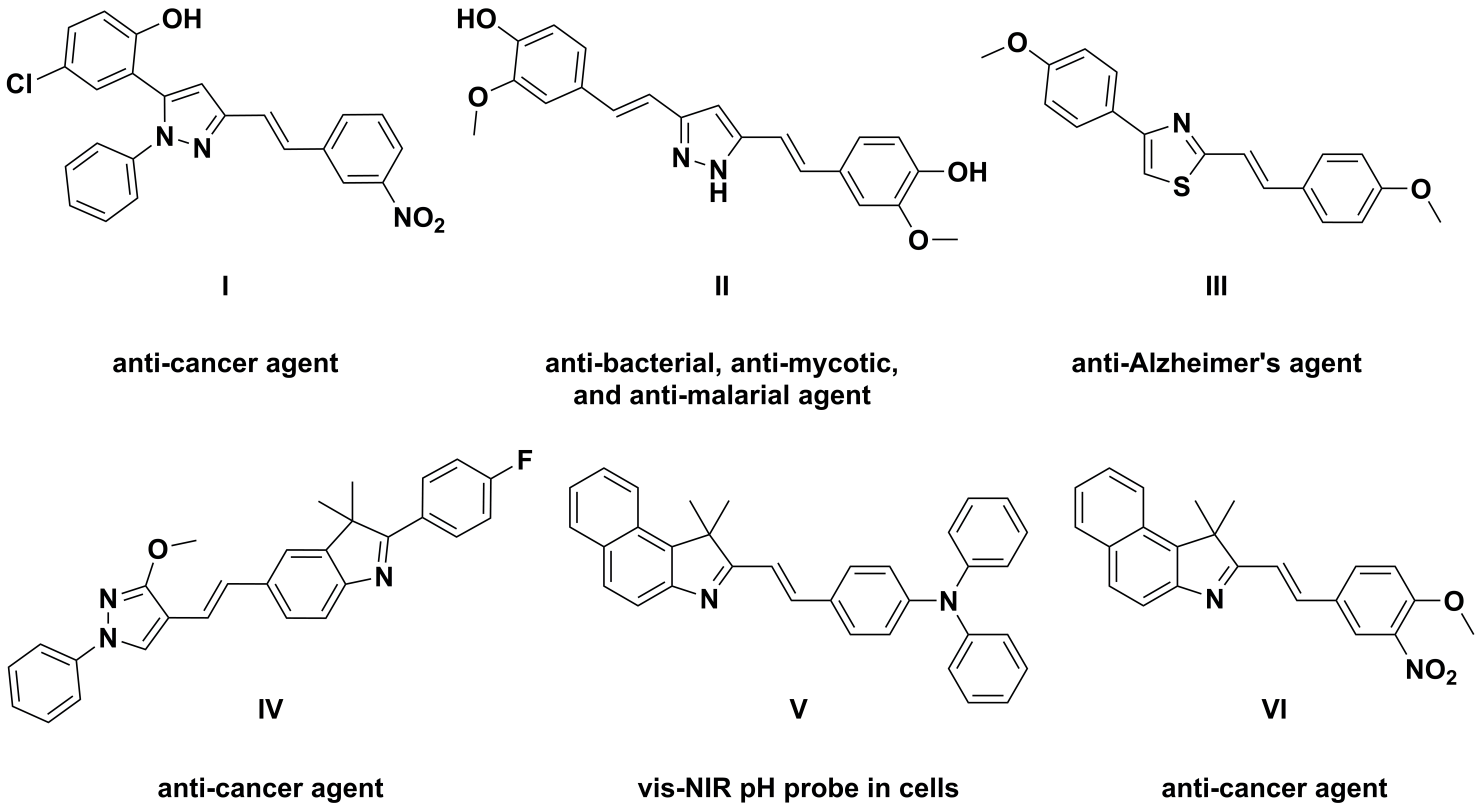
Examples of selected styryl-heterocycle derivatives.

Styryl-heterocycles are an important class of heterocyclic compounds. Their derivatives have numerous applications in various fields, such as biology, pharmaceutical research, medicine, and material sciences [1,2]. Many styryl-heterocycles containing diverse heterocyclic rings, such as oxadiazole [3], thiophene [4], benzimidazole [5], benzothiazolone [6], pyrazole [7], isoxazole, and isothiazole [8], have demonstrated anti-cancer activity; for instance, styryl-pyrazole **I** exhibits potent anti-cancer effects against bladder cancer cells (Figure 1). Moreover, styryl-pyrazoles showcase a wide variety of biological properties, including anti-bacterial, anti-mycotic [9], anti-malarial [10], anti-oxidant [11], anti-inflammatory [12], and other activities, namely 3,5-bis(styryl)pyrazole **II** [13]. Among the numerous biological properties of styryl-chromones [14], they exhibit selectivity for adenosine receptors, neuroprotection, and α-glucosidase inhibition [15]. In the literature, a series of styryl-pyridines was prepared and evaluated as selective NMDA receptor antagonists [16]. The styryl-thiazole hybrid **III** shows promise as an anti-Alzheimer’s agent [17], and 2-styryl quinolones have been noted for their anti-bacterial activity [18,19]. Antonioletti et al. described the synthesis of 3-(3,4-dimethoxystyryl)thiophene, which significantly inhibits the biosynthesis of ochratoxin A (OTA), a potent mycotoxin that contaminates agricultural products [20]. In addition, some styryl-heterocycles are found in natural compounds and possess interesting biological properties [14,21,22]. Styryl-lactones, especially styryl-pyrones, isolated from *Goniothalamus lanceolatus* plants, have shown cytotoxicity against a panel of human lung and colon cancer cell lines [23,24]. Some styryl-indole hybrids demonstrate strong anti-cancer activity against MCF-7 and G361 cancer cell lines, including the indole-pyrazole hybrid **IV** [25,26].

In recent years, there has been a growing interest in the application of styryl-heterocycles as fluorescent probes for DNA/RNA analysis and visualization in vitro and in cells [27,28,29,30]. For example, Pithan et al. demonstrated that ligands bind to double- and quadruple-stranded DNA in different binding modes at various ligand-to-DNA ratios through photometric and fluorimetric titration with styryl-coralyne derivatives [31]. Fan et al. reported a novel far-visible and near-infrared pH probe **V** based on 2-styryl-benzo[*e*]indole derivatives for intracellular pH imaging of living cells [32]. We recently developed a series of 2-styryl-benzo[*e*]indole dyes that displayed cytotoxic properties in a melanoma cell line when exposed to blue light at submicromolar doses, with derivative **VI** demonstrating the most potent anti-cancer activity. The treatment induced substantial reactive oxygen species generation, which led to DNA damage, followed by cell death [33].

In addition to biological and pharmaceutical activities, styryl-heterocycles have demonstrated interesting photophysical properties. For instance, 1-(2-pyridyl)-4-styrylpyrazoles have been utilized as a highly sensitive and selective switchable fluorescent probe for mercury(II) ions [34]. Kido et al. synthesized an orange-emitting zinc complex containing 2-styryl-8-quinolinolate ligands and employed it as an emitter in organic electroluminescent devices [35].

There are many methods for the preparation of styryl-heterocycles, including Knoevenagel condensation, the Wittig reaction, Mizoroki–Heck coupling, and one-pot synthesis [13,31,36,37,38,39,40,41,42]. It should be noted that one-pot synthesis for the preparation of heterocycles has been widely accepted in scientific research and industry [43,44]. The latter method of synthesis, including cascade reactions [45] and multicomponent reactions [46], has inherent advantages, including reaction and mass efficiency, less waste, low cost, and ease of operation, as it complies with the principles of sustainable chemistry [47]. Generally, multicomponent reactions can be conducted in a domino [48], sequential, or consecutive fashion [49]. Typically, consecutive multicomponent reactions of styryl-heterocycles have multiple operating steps that allow for the gradual introduction of reactants, reagents, and catalysts. For instance, the synthesis of 2-styryl-quinoline compounds via consecutive multicomponent reactions occurs in two steps: first, the Friedländer annulation, which involves 2-aminobenzophenone, β-ketoester, and the catalyst In(OTf)_3_ reacting at 100 °C for 15 minutes with the corresponding 2-methylquinolines to obtain key intermediates; second, the subsequent Knoevenagel condensation is induced by adding aromatic aldehydes [50]. Meyer et al. reported a facile three-component one-pot Suzuki–Knoevenagel synthesis method for merocyanine dyes, which provided access to a wide variety of structurally diverse merocyanine dyes [51]. However, the preparation of 2-styryl-3*H*-indoles, especially their 5-aryl derivatives, by multicomponent reactions via a one-pot synthesis is still poorly explored.

In continuation of our interest in efficient systems for synthesizing styryl-heterocycles, we present the synthesis of novel and diverse fluorescent 5-aryl-2-styryl-3*H*-indole derivatives using microwave-assisted one-pot synthesis, which incorporates Fischer synthesis, the Suzuki reaction, and Knoevenagel condensation reactions.

## 2. Results and Discussion

### 2.1. Chemistry

**Figure 2:**
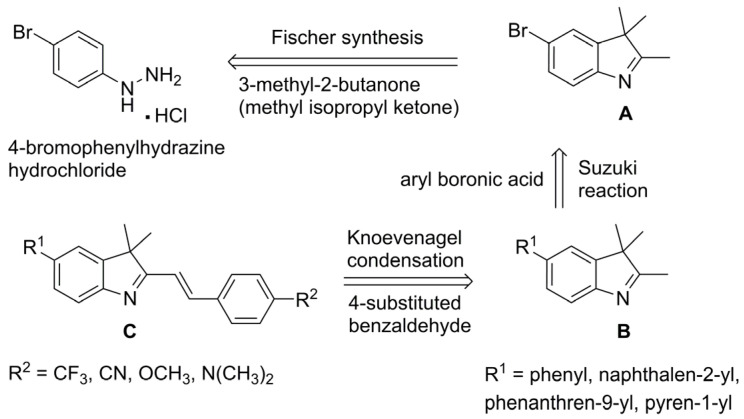
Retrosynthetic analysis and the protocols for one-pot synthesis of 5-aryl-2-styryl-3,3-dimethyl-3*H*-indoles.

We conducted a retrosynthetic analysis to prepare new 5-aryl-2-styryl-3,3-dimethyl-3*H*-indole derivatives using the one-pot synthesis approach, as shown in Figure 2. We determined that 5-bromo-2,3,3-trimethyl-3*H*-indole **A** should be synthesized via Fischer indole synthesis using 4-bromophenylhydrazine hydrochloride and 3-methyl-2-butanone, which would serve as the key intermediates. Then, the Suzuki reaction would yield 5-aryl-2,3,3-trimethyl-3*H*-indoles **B**, and the Knoevenagel condensation of the corresponding 3*H*-indole **B** in the active methyl group with various 4-substituted benzaldehydes would produce 5-aryl-2-styryl-3,3-dimethyl-3*H*-indole derivatives **C**.

An extensive literature search was conducted, providing insights into whether the outlined consecutive multicomponent reactions would work under one-pot conditions. The Fischer reaction (sometimes referred to as Fischer indolization) is one of the most well-established classical methods for synthesizing 3*H*-indole derivatives from substituted phenylhydrazine (or its salt) and 3-methyl-2-butanone (methyl isopropyl ketone) under acidic conditions [52,53]. The reaction is highly versatile due to the wide selection of acid catalysts (such as acetic acid [54], HClO_4_ [55], H_2_SO_4_ [56], and PTSA [57]) and solvents (such as ethanol [55,56] and toluene [57]). For example, Gaur et al. synthesized 2,3,3-trimethyl-3*H*-indole by refluxing phenylhydrazine hydrochloride and 3-methyl-2-butanone in acetic acid for 12 h (85% yield) [58]. Wu et al. reported the synthesis of 5-bromo-2,3,3-trimethyl-3*H*-indole from 4-bromophenylhydrazine hydrochloride and 3-methyl-2-butanone by refluxing in ethanol (with a catalytic amount of H_2_SO_4_) overnight under conventional heating (90% yield) [59]. The microwave-assisted Fischer reaction has also gained popularity due to its reduced reaction time and improved yield. For example, Saha et al. established the microwave-assisted synthesis of 2,3,3-trimethyl-3*H*-indole in acetic acid at 160 °C for 10 min (91% yield) [60]. Moreover, the Fischer reaction does not require dry reaction conditions and can be successfully performed in purely aqueous media under microwave heating. Owens et al. reported a microwave-assisted and environmentally friendly synthesis of 5-bromo-2,3,3-trimethyl-3*H*-indole in water, with a catalytic amount of H_2_SO_4_, which resulted in a quantitative yield [61].

It is well established in the literature that the Suzuki cross-coupling and Knoevenagel condensation reactions can be conducted in various solvents, often including water as a co-solvent [62,63]. Furthermore, the main Fischer reaction by-products are water and ammonium salts, which are unlikely to interfere with the Suzuki and Knoevenagel condensation reaction steps if performed stepwise [52,64]. Therefore, the Fischer reaction seems to be an ideal starting point for developing a Fischer–Suzuki–Knoevenagel consecutive multicomponent reaction using a one-pot synthesis protocol, as all reactions are water-tolerant and can be optimized accordingly.

With these aspects in mind, we began screening the reaction conditions for one-pot synthesis of 5-aryl-2-styryl-3,3-dimethyl-3*H*-indole derivatives, starting with Fischer synthesis. We initiated our work with the most common synthesis method for 5-bromo-2,3,3-trimethyl-3*H*-indole (**2**), which involves the overnight conventional heating of 4-bromophenylhydrazine hydrochloride (**1**) and 3-methyl-2-butanone in ethanol with a catalytic amount of H_2_SO_4_ [65]. In our experiments, after 10 minutes, we observed the formation of the hydrazone intermediate. The reaction proceeded overnight, yielding 55% of the product following purification via column chromatography (Table 1, entry 1). Replacing conventional heating with microwave irradiation (100 °C, 150 W, 10 min) resulted in a slightly higher yield of 58% with reduced reaction time (Table 1, entry 2). Carrying out the reaction in glacial acetic acid under microwave heating did not improve the yield (Table 1, entry 3). The best result was achieved when the reaction was conducted according to the Owens et al. procedure [61]; however, this resulted in a lower yield than that reported by the authors (Table 1, entry 4). Notably, additional attempts to improve the yield by prolonging the reaction time, increasing the amount of 3-methyl-2-butanone (Table 1, entry 5), or using solvent mixtures (Table 1, entry 6) were unsuccessful. We observed a full consumption of the starting material and hydrazone intermediate in all instances. Thus, the conditions using water and H_2_SO_4_ were selected as the most suitable for further synthesis optimization (Table 1, entry 4).

Next, it was essential to screen the Suzuki cross-coupling reaction conditions. According to the available literature, 5-(hetero)aryl-2,3,3-trimethyl-3*H*-indole derivatives are mainly synthesized using the Pd(PPh_3_)_4_ catalyst and K_2_CO_3_ as a base in various solvent mixtures with water, such as toluene/water [66], DME/water [65], and THF/water [67]. Surprisingly, there are no reported examples of employing simpler catalysts, such as Pd(OAc)_2_, utilizing microwave heating, or using more environmentally friendly solvent mixtures like ethanol/water. Water and protic polar solvents (e.g., ethanol) play a crucial role in activating Pd(OAc)_2_, rapidly increasing the formation rate of the active Pd(0) catalyst required for Suzuki cross-coupling [68,69]. Our group previously published a ligandless Suzuki–Miyaura cross-coupling protocol for coupling various brominated imidazo [1,2–*a*] indolone and pyrimido [1,2–*a*] indolone derivatives in ethanol/water media, using only a Pd(OAc)_2_ catalyst and Cs_2_CO_3_ base [70]. Therefore, we implemented these conditions for our one-pot synthesis optimization. Since Pd(PPh_3_)_4_ is a standard catalyst used in Suzuki cross-coupling reactions for these compounds, we initially conducted the reaction in an EtOH:H_2_O (3:1) solvent mixture with the Cs_2_CO_3_ base and Pd(OAc)_2_/PPh_3_ catalytic system, which generates Pd(PPh_3_)_4_ in situ under microwave irradiation at 80 °C for 40 min (Table 2, entry 1). After purification, the desired product **3** was obtained with an overall yield of 45% (the Suzuki reaction step resulted in a 67% yield). After the same duration but using conventional heating, the reaction remained incomplete (containing some compound **2**) and achieved a 35% yield (Table 2, entry 2). Extending the reaction time to completion (2 h), a slightly higher product yield was obtained (Table 2, entry 3). A similar yield with a shorter reaction time (30 min) was achieved when Pd(OAc)_2_ loading was reduced from 10% to 5%, and the reaction temperature was increased to 100 °C under microwave heating (Table 2, entry 4). Furthermore, omitting the PPh_3_ ligand (Table 2, entry 5) and using the weaker base K_2_CO_3_ had minimal effects on the reaction yield (Table 2, entry 6). However, when 1.1 equivalents of 2-naphthylboronic acid were used, the overall yield decreased to 29% (the Suzuki reaction step yield was 43%) (Table 2, entry 7). Thus, for the Suzuki cross-coupling step, we selected optimal conditions, which are ligandless, utilize a relatively more air and moisture-stable Pd(OAc)_2_ (compared to standard Pd(PPh_3_)_4_), employ K_2_CO_3_ as a base, and are performed in a polar protic reaction medium at 100 °C for 30 minutes under microwave irradiation (Table 2, entry 6).

**Scheme 1:**
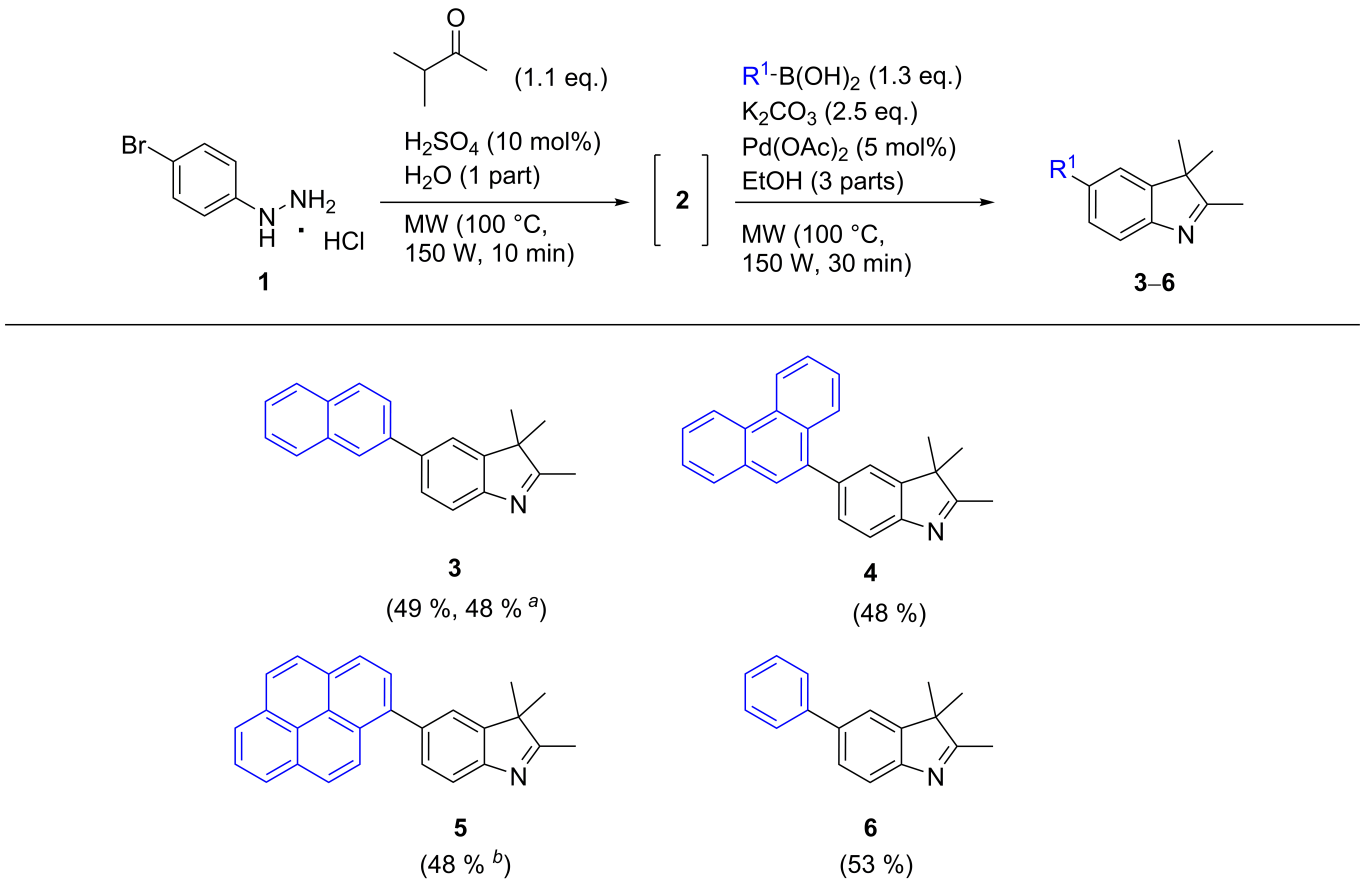
Fischer–Suzuki one-pot synthesis of 5-aryl-2,3,3-trimethyl-3*H*-indole derivatives **3**–**6** under optimal reaction conditions. Overall yields are provided for the entire Fischer–Suzuki one-pot procedure. ^*a*^ Isolated yield over two separate steps. ^*b*^ Suzuki cross-coupling reaction time of 40 min.

With optimal conditions established, we then tested and compared the two-step and one-pot synthesis approaches for synthesizing compound **3** utilizing the optimal conditions from Table 1 and Table 2. The catalytic amount of H_2_SO_4_ from the Fischer indolization step does not interfere with the Suzuki cross-coupling step, as it is neutralized to K_2_SO_4_ by adding excess K_2_CO_3_. As anticipated, the overall compound **3** yields were nearly identical, regardless of whether the synthesis was performed in two separate steps or as a one-pot reaction, resulting in 48% and 49% yields, respectively (Scheme 1). These results indicate no significant difference in reaction yield; therefore, a one-pot strategy could be selected as an easier and faster method for compound **3** synthesis. Additionally, it was discovered that compound **2** is not stable upon prolonged storage and tends to degrade over time. Therefore, an additional advantage of adopting a one-pot synthesis strategy is that compound **2** can be newly prepared each time and promptly used in Suzuki cross-coupling.

Furthermore, we investigated the applicability of the Fischer–Suzuki one-pot synthesis for producing other 5-aryl-2,3,3-trimethyl-3*H*-indoles. As illustrated in Scheme 1, the Fischer–Suzuki one-pot reaction proceeds very similarly with compounds **3**–**5** and exhibits good yields in the Suzuki cross-coupling reaction, regardless of the aromatic ring size (Suzuki reaction step yield: 72–73%; overall yield: 48–49%). Interestingly, when phenyl boronic acid was used, compound **6** was obtained with a higher yield (Suzuki cross-coupling step yield: 79%; overall yield: 53%) than other more structurally complex 5-aryl-2,3,3-trimethyl-3*H*-indole derivatives **3**–**5**. Nevertheless, these results suggest that the Fischer–Suzuki one-pot methodology can be effectively employed for the fast and efficient synthesis of various 5-aryl-2,3,3-trimethyl-3*H*-indoles.

Lastly, we aimed to integrate the Knoevenagel condensation reaction with the optimized Fischer–Suzuki method into a unified Fischer–Suzuki–Knoevenagel one-pot synthesis protocol. This method accelerates the overall synthesis process of 5-aryl-2-styryl-3,3-dimethyl-3*H*-indole derivatives by removing additional purification steps and combining the entire synthesis into one continuous one-pot process. To accomplish this, we initially screened the Knoevenagel condensation reaction conditions between 4-(trifluoromethyl)benzaldehyde and compound **3** (Table 3). The most common synthesis method for 2-styryl-3,3-dimethyl-3*H*-indole derivatives involves refluxing 2,3,3-trimethyl-3*H*-indole (or its salt) with substituted benzaldehyde in ethanol alone [71] or with a catalytic amount of piperidine [72]. In our case, the reaction mixture contained excess K_2_CO_3_ and several other inorganic salts, which were Fischer and Suzuki reaction by-products, making the reaction medium basic. However, the reaction without an additional catalyst (Table 3, entry 1) or with 0.5 eq. of piperidine (Table 3, entry 2) only generated traces of the product, leaving most of compound **3** unreacted. Adding the *L*-proline catalyst also did not improve the reaction (Table 3, entry 3). These results suggest that the basic medium is not sufficient for Knoevenagel condensation. According to the literature, conducting Knoevenagel condensation under acidic conditions requires employing acid catalysts [73]. Although adding 10 eq. of acetic acid did not lead to significant improvements (Table 3, entry 4), adding a volumetrically equal amount of acetic acid to the reaction mixture (four parts or 50% of the final volume) resulted in the complete consumption of compound **3** (Table 3, entry 5). The reaction was completed in 20 min, with a 29% overall yield for compound **7** (the Knoevenagel condensation step yield was 60%). In this context, acetic acid was used to neutralize residual K_2_CO_3_ from the Suzuki cross-coupling reaction, generating a KOAc/AcOH buffer system beneficial for the Knoevenagel step. Therefore, the conditions involving the addition of acetic acid were selected to finalize the Fischer–Suzuki–Knoevenagel one-pot protocol.

**Scheme 2:**
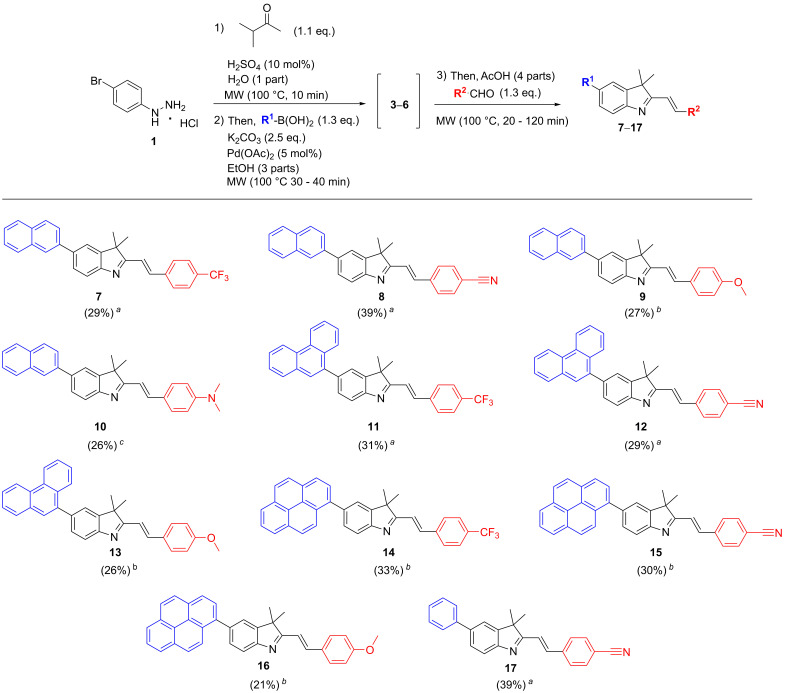
Fischer–Suzuki–Knoevenagel one-pot synthesis of 5-substituted 2-styryl-3*H*-indole derivatives **7**–**17** under optimal reaction conditions. Overall yields are provided for the entire Fischer–Suzuki–Knoevenagel one-pot procedure. Knoevenagel condensation reaction times of *^a^* 20 min, ^*b*^ 40 min, and ^*c*^ 120 min.

Once the optimal conditions for the Fischer–Suzuki–Knoevenagel one-pot protocol were established, we delved deeper into its scope by testing a range of different 4-substituted benzaldehydes. As shown in Scheme 2, the entire synthesis duration primarily depended on Knoevenagel condensation and the nature of the substituents on the aromatic aldehydes. Electron-donating substituents, such as -N(CH_3_)_2_ and -OCH_3_, resulted in prolonged Knoevenagel condensation. In contrast, reactions using aldehydes with electron-withdrawing substituents, such as -CN and -CF_3_, proceeded much faster and with higher yields, possibly due to electron-donating substituents increasing electron density at the carbonyl, making it less electrophilic and requiring a longer reaction time. Conversely, electron-withdrawing groups decrease electron density, making the carbonyl more electrophilic and thus more susceptible to nucleophilic attacks. In summary, the entire Fischer–Suzuki–Knoevenagel one-pot synthesis produced yields in the 21–39% range for compounds **7**–**17**.

### 2.2. NMR Spectroscopic Investigations

**Figure 3:**
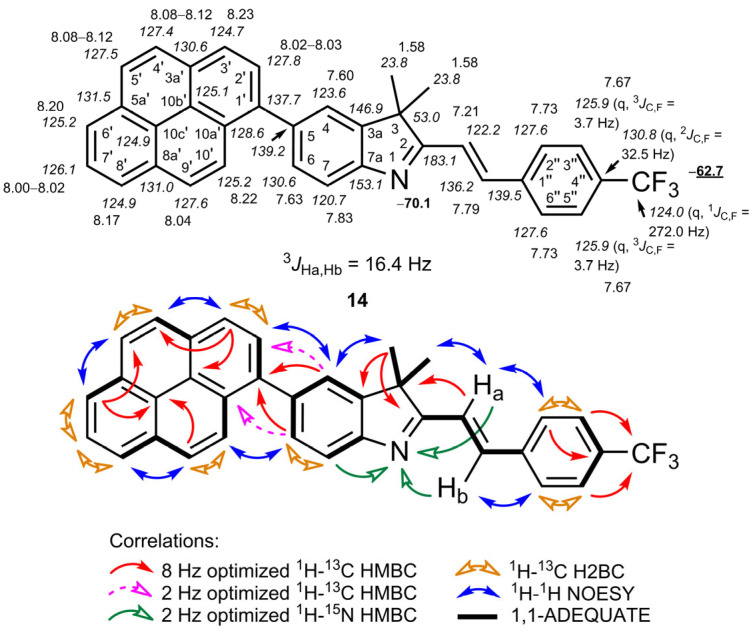
^1^H NMR, ^13^C NMR (italics), ^15^N NMR (bold), and ^19^F NMR (bold, underlined) chemical shifts and relevant ^1^H-^15^N HMBC, ^1^H-^13^C HMBC, 1,1-ADEQUATE, ^1^H-^1^H NOESY, and ^1^H-^13^C H2BC correlations of compound **14**.

The structures of all new 5-aryl-3*H*-indoles **3**–**6** and their 5-aryl-2-styryl-3*H*-indole derivatives **7**–**17** were unambiguously confirmed via an in-depth analysis of multinuclear NMR spectroscopy, infrared (IR) spectroscopy, and high-resolution mass spectrometry (HRMS) data. The spectroscopic data for all compounds investigated in this study are given in Section 3 and Supplementary Materials. The combined application of standard and advanced NMR spectroscopic techniques, such as ^1^H-^13^C HMBC, ^1^H-^13^C HSQC, ^1^H-^13^C H2BC, ^1^H-^15^N HMBC, ^1^H-^1^H COSY, ^1^H-^1^H TOCSY, ^1^H-^1^H NOESY, and 1,1-ADEQUATE experiments, confirmed an unequivocal assignment of the signals. Data analysis showed that the chemical shift values were highly consistent within each compound series, thus validating the shifts for each position. The corresponding NMR data for the representative compound **14** are shown in Figure 3.

The aforementioned new 3*H*-indoles contain one distinct nitrogen atom, which appears significantly downfield compared to a regular ^15^N chemical shift range. This key information was easily obtained via long-range ^1^H-^15^N HMBC correlations of the N-1 nitrogen atom, with neighboring 7-H indole ring proton and 2-methyl or 2-styryl moiety protons, respectively. The chemical shifts for the N-1 nitrogen atom of compounds **3**–**6** were in a range from δ −75.2 to −76.0 ppm and similar to the values of styryl-like derivatives **9**, **10**, **13**, and **16**, containing electron-donating substituents, such as -N(CH_3_)_2_ and -OCH_3_, and resonating in a range from δ −77.4 to −82.6 ppm. Meanwhile, compounds **7**-, **8**-, **11**-, **12**-, **14**-, **15**-, and **17**-containing electron-withdrawing substituents (-CN and -CF_3_) resonated slightly downfield (from δ −67.2 to −70.5 ppm), which is in good agreement with the literature [26].

In the case of styryl-like 3,3-dimethyl-5-(pyren-1-yl)-2-{(*E*)-2-[4-(trifluoromethyl)phenyl]ethenyl}-3*H*-indole **14**, the ^1^H-^15^N HMBC spectral data provided key information, allowing us to identify neighboring protons relative to the N-1 nitrogen. Strong long-range correlations were observed between the aforementioned nitrogen atom and three sets of ^1^H signals, an indole 7-H proton (doublet, δ 7.83 ppm), the olefinic H_a_ (doublet, δ 7.21 ppm), and H_b_ (doublet, δ 7.79 ppm) protons from an ethene bridge unit. The *E*-configuration of the ethene double bond unequivocally follows from the vicinal coupling magnitude between the olefinic protons H_a_ and H_b_, which exhibited an AB-spin system and appeared as two sets of doublets (^3^*J*_Ha,Hb_ = 16.4 Hz). The multiplicity-edited ^1^H-^13^C HSQC spectrum showed that these protons have a one-bond connectivity with the ethene bridge carbons CH_a_ (δ 122.2 ppm) and CH_b_ (δ 136.2 ppm), respectively. This finding, together with data from the 1,1-ADEQUATE experiment, allowed us to unambiguously assign the quaternary ^13^C signals of an adjacent indole C-2 carbon (δ 183.1 ppm) and C-1″ carbon (δ 139.5 ppm) from the 4-trifluoromethylphenyl moiety, thus affirming the connection between these different structural fragments via the ethene bridge. ^1^H-^13^C HMBC spectral data further supported the abovementioned findings, which showed strong long-range correlations between the olefinic proton H_a_ and distinct indole ring C-3 quaternary carbon (δ 53.0 ppm). As expected, the olefinic proton H_b_ showed strong long-range HMBC correlations with the neighboring C-2″,6″ carbons from the 4-trifluoromethylphenyl moiety.

The ^19^F NMR spectrum confirmed a typical chemical shift for the CF_3_ group, which resonated at δ –62.7 ppm. Moreover, the ^13^C NMR spectrum exhibited characteristic resonances of the 4-trifluoromethylphenyl moiety, where the CF_3_ group was observed as a quartet at δ 124.0 ppm (^1^*J*_C,F_ = 272.0 Hz), while the C-4″ and C-3″,5″ carbons appeared as quartets at δ 130.8 ppm (^2^*J*_C,F_ = 32.5 Hz) and δ 125.9 ppm (^3^*J*_C,F_ = 3.7 Hz), respectively. These assignments followed from the difference between the magnitudes of *J*_C,F_-coupling constants. Furthermore, 4-trifluoromethylphenyl ring protons 3″,5″-H (δ 7.67 ppm) were easily resolved, as they showed HSQC connectivity with carbons C-3″,5″ (δ 125.9 ppm). Then, the assignment of adjacent protonated carbons C-2″,6″ was easily achieved from the ^1^H-^13^C H2BC spectral data.

Finally, the NOESY spectral data allowed an unambiguous structural elucidation based on through-space correlations, where all the different structural fragments throughout the 3,3-dimethyl-5-(pyren-1-yl)-2-{(*E*)-2-[4-(trifluoromethyl)phenyl]ethenyl}-3*H*-indole could be joined, thus confirming their proximity in space. For instance, the ^1^H-^1^H NOESY spectrum not only revealed distinct NOEs between the well-resolved indole 4-H proton (singlet, δ 7.60 ppm) and the neighboring geminal methyl groups (singlet, δ 1.58 ppm) but also with the 2-H′ proton (δ 8.02–8.03 ppm) from the pyren-1-yl moiety. The distinction between the neighboring pyren-1-yl moiety C-1′, C-2′, and C-10a′ carbons was achieved by comparing the long-range 2 Hz and 8 Hz optimized ^1^H-^13^C HMBC spectra, where correlations with indole 4-H (δ 7.60 ppm) and 6-H (δ 7.63 ppm) protons were easily observed. This finding was unambiguously confirmed from 1,1-ADEQUATE spectral data, where the protonated methine carbon C-2′ (δ 127.8 ppm) showed a sole correlation with quaternary carbon C-1′ at δ 137.7 ppm. With this information in hand, the distinct ^1^H spin systems of the pyren-1-yl moiety were carefully assigned using a combination of ^1^H-^1^H COSY, ^1^H-^1^H TOCSY, and ^1^H-^1^H NOESY spectral data. Lastly, this allowed the assignment of the remaining ^13^C signals using ^1^H-^13^C HMBC, ^1^H-^13^C HSQC, ^1^H-^13^C H2BC, and 1,1-ADEQUATE experiments.

### 2.3. Optical Properties

The absorption spectra of intermediates **3**–**6** and newly synthesized 5-aryl-2-styryl-3*H*-indoles **7**–**17** were recorded in THF (Table 4; the representative spectra are shown in Figures S1 and S3). Among all compounds investigated, only compound **10** exhibited absorption in the visible part of the electronic spectrum (λ_abs_ = 412 nm) (Table 4, entry 8). As anticipated, the absorption maximum of pyrenyl-substituted 3*H*-indole **5** was the most redshifted (λ_abs_ = 344 nm) due to its larger aromatic system compared to compounds **3**, **4**, and **6**. Interestingly, incorporating the phenanthrenyl group led to a blueshifted absorption for compound **4** (λ_abs_ = 300 nm) compared to compound **3**, which bears a smaller naphthalenyl group (λ_abs_ = 304 nm) (Table 4, entries 1–2). Introducing the styryl moiety into the structure significantly induces a bathochromic shift in the absorption maximum for all compounds. However, the influence of the aryl substituent size on the absorption maximum shift is minimal. For example, the absorption maximum of compound **5** is redshifted by 40 nm compared to compound **3** (Table 4, entries 1 and 3), while its styryl analogs **7**–**9** display λ_abs_ (Table 4, entries 5–7) that are 1–2 nm more redshifted than compounds **14**–**16** (Table 4, entries 12–14), which possess a larger aromatic conjugated system.

**Figure 4:**
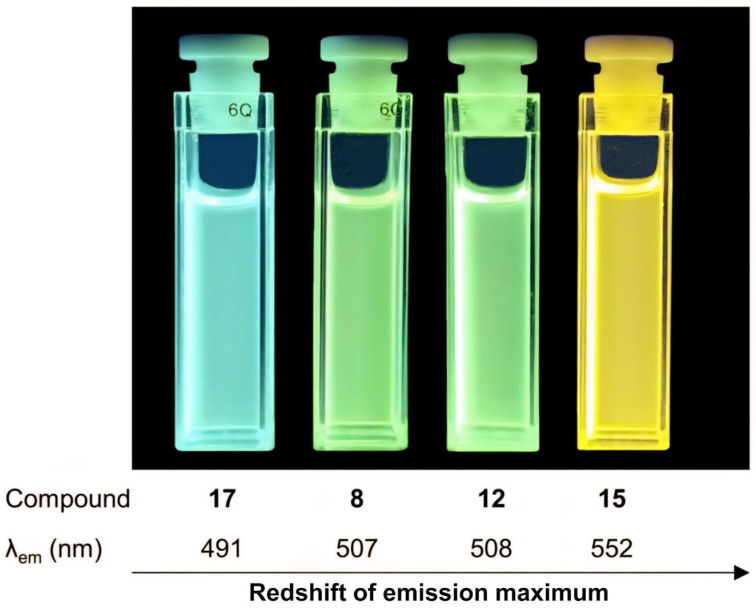
The visual representation of the redshift in the emission maximum value with the aryl substituent size in compounds **17**, **8**, **12**, and **15**.

The fluorescence spectra of intermediates **3**–**6** and 5-aryl-2-styryl-3*H*-indoles **7**–**17** (Table 4; the representative spectra are shown in Figures S2 and S4) were recorded in THF, and their fluorescence quantum yield (Φ*_f_*) was estimated using the integrating sphere method. Both the fluorescence quantum yield (Φ*_f_*) and the emission maxima (λ_em_) redshift were found to increase with larger aryl substituents for intermediates **3**–**5** (Table 4, entries 1–3). Surprisingly, the phenyl-substituted compound **6** exhibited a similar emission maximum value as compound **3** but with a higher Stokes shift and Φ*_f_* (Table 4, entry 4). The emission maxima (λ_em_) of the final compounds **7**–**17** were in the 448–552 nm range, which falls within the visible part of the spectrum. As seen in Table 4, increased aryl substituent size caused emission maxima (λ_em_) redshifts in most compounds. For example, the pyrenyl group caused an emission maxima (λ_em_) redshift for compounds **14**–**16** (Table 4, entries 12–14) compared to compounds **7**–**9** (Table 4, entries 5–7), **11**–**13** (Table 4, entries 9–11), and **17** (Table 4, entry 15), with their respective substituents. However, like the absorption maxima blueshift case, substitution with the phenanthrenyl group also resulted in an emission maxima (λ_em_) blueshift for compounds **11**–**13** (Table 4, entries 9–11) compared to the naphthalenyl-substituted variants **7**–**9**. Notably, the final compounds **7**–**17** exhibit large Stokes shifts, which increase with aryl substituent size, with compound **15** showing the largest Stokes shift (Figure 4).

The fluorescence quantum yield (Φ*_f_*) values of the final compounds strongly depended on the electron-donating or -withdrawing nature of the substituents on the phenyl ring. Electron-donating substituents, such as -N(CH_3_)_2_ and -OCH_3_, found in compounds **9**, **10**, **13**, and **16**, negatively impacted the fluorescence quantum yield (Φ*_f_*), which was estimated to be as high as 5.4%. Compound **10**, which bears the dimethylamino (-N(CH_3_)_2_) substituent, exhibited the lowest Φ*_f_* (Table 4, entry 8). In contrast, compounds **7**, **8**, **11**, **12**, **14**, **15**, and **17,** which contained electron-withdrawing substituents, like -CF_3_ and -CN, displayed high fluorescence quantum yields (Φ*_f_*), ranging from 42.2% to 71.5%. The highest Φ*_f_* values were observed in compounds **8**, **12**, **15**, and **17**, all containing a -CN substituent, yielding values of 71.5%, 67.9%, 71.0%, and 53.5%, respectively.

## 3. Materials and Methods

### 3.1. General

All chemicals and solvents were purchased from commercial suppliers and used without further purification unless otherwise specified. The ^1^H, ^13^C, and ^15^N NMR spectra were recorded in CDCl_3_ solutions at 25 °C on a Bruker Avance III 700 (700 MHz for ^1^H, 176 MHz for ^13^C, and 71 MHz for ^15^N) spectrometer (Bruker BioSpin GmbH, Rheinstetten, Germany) equipped with a 5 mm TCI ^1^H-^13^C/^15^N/D z-gradient cryoprobe, or a Bruker Avance III 400 (400 MHz for ^1^H, 101 MHz for ^13^C, and 41 MHz for ^15^N) using a directly detecting BBO probe (Bruker BioSpin International *AG*, Faellanden, Switzerland). The chemical shifts expressed in parts per million (ppm) were relative to tetramethylsilane (TMS). The ^15^N NMR spectra were referenced to neat, external nitromethane (coaxial capillary). ^19^F NMR spectra (376 MHz, absolute referencing via Ξ ratio) were obtained on a Bruker Avance III 400 using a directly detecting BBO probe. FT-IR spectra were collected using the ATR method on a Bruker Vertex 70v spectrometer (Bruker Optik GmbH, Ettlingen, Germany) with an integrated Platinum ATR accessory or on a Bruker Tensor 27 spectrometer (Bruker Optik GmbH, Ettlingen, Germany) in KBr pellets. The melting points of crystalline compounds were determined in open capillary tubes with a Buchi M 565 apparatus (Büchi Labortechnik AG, Flawil, Switzerland) and are uncorrected (temperature gradient—2 °C/min). High-resolution mass spectrometry (HRMS) spectra were obtained in ESI mode on a Bruker MicrOTOF-Q III spectrometer (Bruker Daltonik GmbH, Bremen, Germany). Accurate measurements were achieved using the internal mass calibration of each sample using sodium formate calibration solution as a standard procedure, with a standard deviation always less than 1 ppm. In addition, all data files were recalibrated with an internal standard of sodium formate injected prior to initial elution for each sample. The UV-vis spectra were recorded using 10^−4^ M solutions of the compounds in THF on a Shimadzu 2600 UV/Vis spectrometer (Shimadzu Corporation, Kyoto, Japan). The fluorescence spectra were recorded on a FL920 fluorescence spectrometer from Edinburgh Instruments (Edinburgh Analytical Instruments Limited, Edinburgh, UK). The fluorescence quantum yields were measured from dilute THF solutions by an absolute method using the Edinburgh Instruments integrating sphere excited with an Xe lamp. Optical densities of the sample solutions were ensured to be below 0.1 to avoid reabsorption effects. All optical measurements were performed at room temperature under ambient conditions. Microwave reactions were conducted using a CEM Discover Synthesis Unit (CEM Corp., Matthews, NC, USA) and performed in glass vessels (capacity: 10 mL), sealed with a septum. Reactions with conventional heating were performed in oven-dried flasks under an argon atmosphere with magnetic stirring. Reaction progress was monitored by TLC analysis on Macherey-Nagel™ ALUGRAM® Xtra SIL G/ UV254 plates (Macherey-Nagel GmbH & Co. KG, Düren, Germany). TLC plates were visualized with UV light (wavelengths 254 and 365 nm) or iodine vapor. Compounds were purified by chromatography in a glass column (stationary phase: silica gel, high-purity grade: 9385, pore size: 60 Å, particle size: 230–400 mesh, supplier: Sigma-Aldrich; Merck KGaA, Darmstadt, Germany).

### 3.2. Synthetic Procedures

#### 3.2.1. Synthesis of 5-Bromo-2,3,3-trimethyl-3*H*-indole (**2**)

Synthesis of 5-bromo-2,3,3-trimethyl-3*H*-indole was accomplished by modifying a previously reported procedure [61]. 4-Bromophenylhydrazine hydrochloride (500 mg, 2.237 mmol, 1 eq) was added to a microwave vessel with a magnetic stir bar along with 3-methyl-2-butanone (212 mg, 0.264 mL, 2.46 mmol, 1.1 eq.), H_2_SO_4_ (44 mg, 0.447 mmol, 0.2 eq.), and H_2_O (0.8 mL). The reaction mixture was purged with argon, the vessel was securely capped, and then it was irradiated (150 W) at 100 °C for 10 min. Upon completion, the reaction mixture was basified with K_2_CO_3_, diluted with H_2_O (10 mL), and extracted with EtOAc (2 × 20 mL). The combined organic layers were washed with brine (10 mL), dried over anhydrous Na_2_SO_4_, filtered, and concentrated under reduced pressure. The residue was purified by column chromatography (ethyl acetate/*n*-hexane, 1:4 *v*/*v*) to afford compound **2** as red-brown oil (362 mg, 67%). ^1^H NMR (400 MHz, CDCl_3_): *δ*_H_ 7.44–7.37 (m, 3H), 2.26 (s, 3H), 1.30 (s, 6H). ^13^C NMR (101 MHz, CDCl_3_): *δ*_C_ 188.5, 152.7, 147.8, 130.6, 124.8, 121.3, 118.8, 54.1, 22.9, 15.4.

#### 3.2.2. General Procedures for the Synthesis of 5-aryl-2,3,3-trimethyl-3*H*-indoles (**3**–**6**)

Procedure A (Suzuki cross-coupling): In a microwave vessel containing 5-bromo-2,3,3-trimethyl-3*H*-indole **2** (500 mg, 2.1 mmol, 1 eq.), aryl boronic acid (1.3 eq.), and K_2_CO_3_ (725 mg, 5.25 mmol, 2.5 eq.) was added to a 3:1 EtOH/H_2_O (3.2 mL) solvent mixture. The mixture was purged with argon for 10-15 min. After that, Pd(OAc)_2_ (23 mg, 0.105 mmol, 0.05 eq.) was added to the mixture, which was then subjected to microwave irradiation (150 W) at 100 °C for the indicated time (its progress was monitored by TLC). Upon completion, the reaction mixture was cooled to room temperature and vacuum filtered, and the resulting filter cake was washed with EtOAc (20 mL). Filtrate was diluted with water (10 mL) and extracted with EtOAc (2 × 20 mL). The combined organic layers were washed with brine (10 mL), dried over anhydrous Na_2_SO_4_, filtered, and concentrated under reduced pressure. The residue was purified by column chromatography.

Procedure B (Fischer–Suzuki one-pot reaction): A sealed microwave vessel containing a mixture of 4-bromophenylhydrazine hydrochloride (500 mg, 2.237 mmol, 1 eq.), 3-methyl-2-butanone (212 mg, 0.264 mL, 2.46 mmol, 1.1 eq.), and H_2_SO_4_ (44 mg, 0.447 mmol, 0.2 eq.) in H_2_O (0.8 mL) was irradiated (150 W) under argon atmosphere at 100 °C for 10 min. The resulting mixture was then basified with K_2_CO_3_ (773 mg, 5.6 mmol, 2.5 eq.), followed by the addition of the corresponding boronic acid (1.3 eq.) and EtOH (2.4 mL). The mixture was purged with argon for 10-15 min. After that, Pd(OAc)_2_ (25 mg, 0.112 mmol, 0.05 eq.) was added to the mixture, which was then subjected to microwave irradiation (150 W) at 100 °C for the indicated time (its progress was monitored by TLC). Upon completion, the reaction mixture was cooled to room temperature and vacuum filtered, and the resulting filter cake was washed with EtOAc (20 mL). Filtrate was diluted with water (10 mL) and extracted with EtOAc (2 × 20 mL). The combined organic layers were washed with brine (10 mL), dried over anhydrous Na_2_SO_4_, filtered, and concentrated under reduced pressure. The residue was purified by column chromatography.

##### 2,3,3-Trimethyl-5-(naphthalen-2-yl)-3*H*-indole (**3**)

This was prepared following the general procedures A and B described above. The cross-coupling was performed with 2-naphthylboronic acid (for procedure A, 470 mg, 2.73 mmol; for procedure B, 500 mg, 2.91 mmol), and the reaction time was 30 min. The residue was purified by column chromatography [ethyl acetate/toluene, 1:6 → acetone/*n*-hexane, 1:8 (*v*/*v*)] to afford compound **3** as yellowish crystals. Procedure A (432 mg, 72%). Procedure B (313 mg, overall yield 49%, Suzuki reaction yield 73%). M.p. 145–147 °C. ^1^H NMR (700 MHz, CDCl_3_): *δ*_H_ ppm 8.05 (s, 1H, Naph 1-H), 7.93–7.91 (m, 1H, Naph 4-H), 7.91–7.89 (m, 1H, Naph 8-H), 7.87 (d, *J* = 7.9 Hz, 1H, Naph 5-H), 7.77 (d, *J* = 8.4 Hz, 1H, Naph 3-H), 7.67 (d, *J* = 7.9 Hz, 1H, Ind 6-H), 7.63 (d, *J* = 8.0 Hz, 1H, Ind 7-H), 7.62 (s, 1H, Ind 4-H), 7.51 (t, *J* = 7.3 Hz, 1H, Naph 7-H), 7,48 (t, *J* = 7.4 Hz, 1H, Naph 6-H), 2.32 (s, 3H, Ind 2-CH_3_), 1.39 (s, 6H, Ind 3-(CH_3_)_2_). ^13^C NMR (176 MHz, CDCl_3_): *δ*_C_ ppm 188.4 (Ind C-2), 153.3 (Ind C-7a), 146.4 (Ind C-3a), 138.8 (Naph C-2), 138.3 (Ind C-5), 133.7 (Naph C-8a), 132.5 (Naph C-4a), 128.4 (Naph C-4), 128.1 (Naph C-8), 127.7 (Naph C-5), 127.1 (Ind C-6), 126.3 (Naph C-7), 125.9 (Naph C-6), 125.8 (Naph C-3), 125.7 (Naph C-1), 120.5 (Ind C-4), 120.1 (Ind C-7), 53.8 (Ind C-3), 23.2 (Ind 3-(CH_3_)_2_), 15.6 (Ind 2-CH_3_). ^15^N NMR (71 MHz, CDCl_3_): *δ*_N_ ppm –75.4 (Ind N-1). IR (neat, *ν_max_*, cm^−1^): 3048, 3024 (C-H_aromatic_), 2959, 2922, 2897, 2865, 2837 (C-H_aliphatic_), 1599, 1522, 1504, 1451, 1356, 1186, 1111, 973, 818, 810, 746 (C=C, C=N, C-N, =C-H). HRMS (ESI-TOF): found: [M + H]^+^ 286.1590; molecular formula C_21_H_20_N requires [M + H]^+^ 286.1590.

##### 2,3,3-Trimethyl-5-(phenanthren-9-yl)-3*H*-indole (**4**)

This was prepared following the general procedure B described above. The cross-coupling was performed with 9-phenanthracenylboronic acid (646 mg, 2.91 mmol), and the reaction time was 30 min. The residue was purified by column chromatography [ethyl acetate/toluene, 1:6 → acetone/*n*-hexane, 1:8 (*v*/*v*)] to afford compound **4** as a yellowish powder (338 mg, overall yield 48%). M.p. 68–70 °C. ^1^H NMR (400 MHz, CDCl_3_): *δ*_H_ ppm 8.77 (d, *J* = 8.3 Hz, 1H, Phen 5-H), 8.72 (d, *J* = 8.2 Hz, 1H, Phen 4-H), 7.94 (d, *J* = 8.2 Hz, 1H, Phen 8-H), 7.89 (d, *J* = 7.7 Hz, 1H, Phen 1-H), 7.71 (s, 1H, Phen 10-H), 7.69–7.63 (m, 3H, Phen 3-H, Phen 6-H, Ind 7-H), 7.61 (t, *J* = 6.8 Hz, 1H, Phen 2-H), 7.54 (t, *J* = 7.5 Hz, 1H, Phen 7-H), 7.48 (d, *J* = 6.2 Hz, 1H, Ind 6-H), 7.45 (s, 1H, Ind 4-H), 2.34 (s, 3H, Ind 2-CH_3_), 1.37 (s, 6H, Ind 3-(CH_3_)_2_). ^13^C NMR (101 MHz, CDCl_3_): *δ*_C_ ppm 188.4 (Ind C-2), 153.1 (Ind C-7a), 145.8 (Ind C-3a), 138.9 (Phen C-9), 137.7 (Ind C-5), 131.6 (Phen C-10a), 131.3 (Phen C-8a), 130.7 (Phen C-4b), 129.9 (Phen C-4a), 129.5 (Ind C-6), 128.6 (Phen C-1), 127.6 (Phen C-10), 126.92 (Phen C-8), 126.85 (Phen C-2), 126.54 (Phen C-3), 126.50 (Phen C-7), 126.4 (Phen C-6), 123.1 (Ind C-4), 122.9 (Phen C-5), 122.5 (Phen C-4), 119.6 (Ind C-7), 53.8 (Ind C-3), 23.2 (Ind 3-(CH_3_)_2_), 15.5 (Ind 2-CH_3_). ^15^N NMR (41 MHz, CDCl_3_): *δ*_N_ ppm –75.2 (Ind N-1). IR (KBr, *ν_max_*, cm^−1^): 3058, 3028, 3015 (C-H_aromatic_), 2959, 2924, 2860 (C-H_aliphatic_), 1574, 1461, 1450, 1422, 1375, 1248, 1224, 1201, 1116, 1042, 890, 834, 767, 749, 726 (C=C, C=N, C-N, =C-H). HRMS (ESI-TOF): found: [M + H]^+^ 336.1747; molecular formula C_25_H_22_N requires [M + H]^+^ 336.1747.

##### 2,3,3-Trimethyl-5-(pyren-1-yl)-3*H*-indole (**5**)

This was prepared following the general procedure B described above. The cross-coupling was performed with 1-pyrenylboronic acid (716 mg, 2.91 mmol), and the reaction time was 40 min. The residue was purified by column chromatography [ethyl acetate/toluene, 1:6 → acetone/*n*-hexane, 1:8 (*v*/*v*)] to afford compound **5** as a yellowish powder (362 mg, overall yield 48%). M.p. 151–153 °C. ^1^H NMR (700 MHz, CDCl_3_): *δ*_H_ ppm 8.22 (d, *J* = 7.7 Hz, 1H, Pyren 3-H), 8.20 (d, *J* = 7.6 Hz, 1H, Pyren 10-H), 8.19 (d, *J* = 5.0 Hz, 1H, Pyren 8-H), 8.16 (d, *J* = 7.5 Hz, 1H, Pyren 6-H), 8.11–8.07 (m, 2H, Pyren 4-H, Pyren 5-H), 8.04–8.02 (m, 1H, Pyren 9-H), 8.02–8.01 (m, 1H, Pyren 7-H), 8.01–7.99 (m, 1H, Pyren 2-H), 7.71 (d, *J* = 7.7 Hz, 1H, Ind 7-H), 7.57 (d, *J* = 7.8 Hz, 1H, Ind 6-H), 7.53 (s, 1H, Ind 4-H), 2.36 (s, 3H, Ind 2-CH_3_), 1.40 (s, 6H, Ind 3-(CH_3_)_2_). ^13^C NMR (176 MHz, CDCl_3_): *δ*_C_ ppm 188.5 (Ind C-2), 153.1 (Ind C-7a), 145.9 (Ind C-3a), 138.2 (Ind C-5), 137.9 (Pyren C-1), 131.5 (Pyren C-5a), 131.0 (Pyren C-8a), 130.5 (Pyren C-3a), 130.1 (Ind C-6), 128.6 (Pyren C-10a), 127.8 (Pyren C-2), 127.5 (Pyren C-9), 127.4 (Pyren C-4 and C-5), 126.0 (Pyren C-7), 125.3 (Pyren C-10), 125.1 (Pyren C-8), 125.0 (Pyren C-10b), 124.9 (Pyren C-10c), 124.8 (Pyren C-6), 124.6 (Pyren C-3), 123.6 (Ind C-4), 119.6 (Ind C-7), 53.9 (Ind C-3), 23.2 (Ind 3-(CH_3_)_2_), 15.6 (Ind 2-CH_3_). ^15^N NMR (71 MHz, CDCl_3_): *δ*_N_ ppm –75.4 (Ind N-1). IR (neat, *ν_max_*, cm^−1^): 3041 (C-H_aromatic_), 2967, 2955, 2908, 2866 (C-H_aliphatic_), 1600, 1573, 1457, 1429, 1377, 1313, 1233, 1201, 952, 902, 846, 834, 760, 721 (C=C, C=N, C-N, =C-H). HRMS (ESI-TOF): found: [M + H]^+^ 360.1747; molecular formula C_27_H_22_N requires [M + H]^+^ 360.1747.

##### 2,3,3-Trimethyl-5-phenyl-3*H*-indole (**6**)

This was prepared following the general procedure B described above. The cross-coupling was performed with phenylboronic acid (355 mg, 2.91 mmol), and the reaction time was 30 min. The residue was purified by column chromatography [ethyl acetate/toluene, 1:6 → acetone/*n*-hexane, 1:8 (*v*/*v*)] to afford compound **6** as a yellowish powder (274 mg, overall yield 53%). M.p. 99–101 °C. ^1^H NMR (700 MHz, CDCl_3_): *δ*_H_ ppm 7.55–7.49 (m, 3H, Ind 7-H, Ph 2,6-H), 7.45 (dd, *J* = 8.0, 1.8 Hz, 1H, Ind 6-H), 7.41 (d, *J* = 1.8 Hz, 1H, Ind 4-H), 7.36 (t, *J* = 7.6 Hz, 2H, Ph 3,5-H), 7.25 (t, *J* = 7.2 Hz, 1H, Ph 4-H), 2.23 (s, 3H, Ind 2-CH_3_), 1.27 (s, 6H, Ind 3-(CH_3_)_2_). ^13^C NMR (176 MHz, CDCl_3_): *δ*_C_ ppm 188.4 (Ind C-2), 153.1 (Ind C-7a), 146.3 (Ind C-3a), 141.5 (Ph C-1), 138.5 (Ind C-5), 128.8 (Ph C-3,5), 127.3 (Ph C-2,6), 127.1 (Ph C-4), 126.8 (Ind C-6), 120.3 (Ind C-4), 120.0 (Ind C-7), 53.8 (Ind C-3), 23.2 (Ind 3-(CH_3_)_2_), 15.6 (Ind 2-CH_3_). ^15^N NMR (71 MHz, CDCl_3_): *δ*_N_ ppm –76.0 (Ind N-1). IR (neat, *ν_max_*, cm^−1^): 3056, 3023 (C-H_aromatic_), 2964, 2940, 2909, 2866 (C-H_aliphatic_), 1599, 1571, 1461, 1419, 1379, 1302, 1240, 1204, 1122, 1074, 947, 895, 835, 755, 699, 635 (C=C, C=N, C-N, =C-H). HRMS (ESI-TOF): found: [M + H]^+^ 236.1434; molecular formula C_17_H_18_N requires [M + H]^+^ 236.1434.

#### 3.2.3. General Procedure for the Synthesis of 5-aryl-3,3-dimethyl-2-styryl-3*H*-indoles via One-Pot Fischer–Suzuki–Knoevenagel Approach (**7**–**17**)

A sealed microwave vessel containing a mixture of 4-bromophenylhydrazine hydrochloride (250 mg, 1.119 mmol, 1 eq.), 3-methyl-2-butanone (106 mg, 0.132 mL, 1.23 mmol, 1.1 eq.), and H_2_SO_4_ (22 mg, 0.224 mmol, 0.2 eq.) in H_2_O (0.4 mL) was irradiated (150 W) under argon atmosphere at 100 °C for 10 min. The resulting mixture was then basified with K_2_CO_3_ (386 mg, 2.8 mmol, 2.5 eq.), followed by the addition of the corresponding boronic acid (1.3 eq.) and EtOH (1.2 mL). The mixture was purged with argon for 10-15 min. After that, Pd(OAc_2_) (13 mg, 0.056 mmol, 0.05 eq.) was added to the mixture, which was then subjected to microwave irradiation (150 W) at 100 °C for the indicated time (its progress was monitored by TLC). Upon completion, while mixing, AcOH (1.6 mL) was added dropwise to the reaction mixture (vigorous bubbling occurred) and once again purged with argon (2–3 min). Ultimately, the desired aldehyde (1.3 eq.) was added to the reaction mixture and then irradiated (150 W) at 100 °C for the specified time. After the completion of the reaction, as indicated by TLC, the reaction mixture was poured into saturated aqueous Na_2_CO_3_ (10 mL), the reaction vessel was washed with EtOAc into the same saturated aqueous Na_2_CO_3_, and the resulting mixture was extracted with EtOAc (3 × 20 mL). The combined organic layers were washed with brine (10 mL), dried over anhydrous Na_2_SO_4_, filtered, and concentrated under reduced pressure. The residue was purified by column chromatography. The obtained product was additionally washed with EtOH and dried.

##### 3,3-Dimethyl-5-(naphthalen-2-yl)-2-{(*E*)-2-[4-(trifluoromethyl)phenyl]ethenyl}-3*H*-indole (**7**)

The Suzuki cross-coupling was performed with 2-naphthylboronic acid (250 mg, 1.45 mmol), and the reaction time was 30 min. The Knoevenagel condensation was performed with 4-(trifluoromethyl)benzaldehyde (252 mg, 1.45 mmol), and the reaction time was 20 min. The residue was purified by column chromatography [ethyl acetate/toluene, 1:18 → acetone/*n*-hexane, 1:14 (*v*/*v*)], and the obtained product was washed with EtOH and dried to afford compound **7** (143 mg, overall yield 29%). M.p. 216–217 °C. ^1^H NMR (700 MHz, CDCl_3_): *δ*_H_ ppm 8.08 (s, 1H, Naph 1-H), 7.94 (d, *J* = 8.5 Hz, 1H, Naph 4-H), 7.92 (d, *J* = 8.0 Hz, 1H, Naph 8-H), 7.88 (d, *J* = 8.0 Hz, 1H, Naph 5-H), 7.80–7.78 (m, 1H, Naph 3-H), 7.77 (d, *J* = 16.4 Hz, 1H, Ind-CH=CH-Ph), 7.76–7.74 (m, 1H, Ind 7-H), 7.74–7.72 (m, 1H, Ind 6-H), 7.72 (d, *J* = 7.8 Hz, 2H, Ph 2,6-H), 7.68 (s, 1H, Ind 4-H), 7.67 (d, *J* = 8.2 Hz, 2H, Ph 3,5-H), 7.52 (t, *J* = 7.3 Hz, 1H, Naph 7-H), 7.49 (t, *J* = 7.3 Hz, 1H, Naph 6-H), 7.18 (d, *J* = 16.4 Hz, 1H, Ind-CH=CH-Ph), 1.56 (s, 6H, Ind 3-(CH_3_)_2_). ^13^C NMR (176 MHz, CDCl_3_): *δ*_C_ ppm 183.0 (Ind C-2), 153.3 (Ind C-7a), 147.4 (Ind C-3a), 139.5 (Ph C-1), 139.3 (Ind C-5), 138.6 (Naph C-2), 136.1 (Ind-CH=CH-Ph), 133.7 (Naph C-8a), 132.6 (Naph C-4a), 130.8 (q, ^2^*J*_C,F_ = 32.5 Hz, Ph C-4), 128.5 (Naph C-4), 128.2 (Naph C-8), 127.7 (Naph C-5), 127.6 (Ph C-2,6), 127.5 (Ind C-6), 126.4 (Naph C-7), 126.0 (Naph C-6), 125.87 (q, ^3^*J*_C,F_ = 3.8 Hz, Ph C-3,5), 125.83 (Naph C-1), 125.7 (Naph C-3), 124.0 (q, ^1^*J*_C,F_ = 272.0 Hz, -CF_3_), 122.1 (Ind-CH=CH-Ph), 121.2 (Ind C-7), 120.3 (Ind C-4), 53.0 (Ind C-3), 23.9 (Ind 3-(CH_3_)_2_). ^15^N NMR (71 MHz, CDCl_3_): *δ*_N_ ppm –70.5 (Ind N-1). ^19^F NMR (376 MHz, CDCl_3_): *δ*_F_ ppm –62.7 (s, CF_3_). IR (neat, *ν_max_*, cm^−1^): 3045 (C-H_aromatic_), 2960, 2906, 2867 (C-H_aliphatic_), 1609, 1598, 1454, 1413, 1323, 1168, 1111, 1067, 955, 863, 828, 816, 752 (C=C, C=N, CF_3_, C-N, =C-H). HRMS (ESI-TOF): found: [M + H]^+^ 442.1777; molecular formula C_29_H_23_F_3_N requires [M + H]^+^ 442.1777.

##### 4-{(*E*)-2-[3,3-Dimethyl-5-(naphthalen-2-yl)-3*H*-indol-2-yl]ethenyl}benzonitrile (**8**)

The Suzuki cross-coupling was performed with 2-naphthylboronic acid (250 mg, 1.45 mmol), and the reaction time was 30 min. The Knoevenagel condensation was performed with 4-cyanobenzaldehyde (190 mg, 1.45 mmol), and the reaction time was 20 min. The residue was purified by column chromatography [ethyl acetate/toluene, 1:18 → acetone/*n*-hexane, 1:14 (*v*/*v*)], and the obtained product was washed with EtOH and dried to afford compound **8** (174 mg, overall yield 39%). M.p. 190–192 °C. ^1^H NMR (700 MHz, CDCl_3_): *δ*_H_ ppm 8.08 (s, 1H, Naph 1-H), 7.93 (d, *J* = 8.5 Hz, 1H, Naph 4-H), 7.91 (d, *J* = 7.9 Hz, 1H, Naph 8-H), 7.87 (d, *J* = 8.0 Hz, 1H, Naph 5-H), 7.78 (dd, *J* = 8.5, 1.8 Hz, 1H, Naph 3-H), 7.77–7.75 (m, 1H, Ind 7-H), 7.75–7.72 (m, 2H, Ind 6-H, Ind-CH=CH-Ph), 7.69 (s, 4H, Ph 2,6-H, Ph 3,5-H), 7.68 (s, 1H, Ind 4-H), 7.52 (t, *J* = 7.2 Hz, 1H, Naph 7-H), 7.49 (t, *J* = 7.2 Hz, 1H, Naph 6-H), 7.18 (d, *J* = 16.3 Hz, 1H, Ind-CH=CH-Ph), 1.55 (s, 6H, Ind 3-(CH_3_)_2_). ^13^C NMR (176 MHz, CDCl_3_): *δ*_C_ ppm 182.6 (Ind C-2), 153.2 (Ind C-7a), 147.3 (Ind C-3a), 140.4 (Ph C-4), 139.5 (Ind C-5), 138.5 (Naph C-2), 135.6 (Ind-CH=CH-Ph), 133.7 (Naph C-8a), 132.65 (Ph C-2,6), 132.60 (Naph C-4a), 128.5 (Naph C-4), 128.2 (Naph C-8), 127.8 (Ph C-3,5), 127.7 (Naph C-5), 127.5 (Ind C-6), 126.4 (Naph C-7), 126.0 (Naph C-6), 125.9 (Naph C-1), 125.6 (Naph C-3), 123.1 (Ind-CH=CH-Ph), 121.3 (Ind C-7), 120.3 (Ind C-4), 118.7 (-CN), 112.3 (Ph C-1), 53.0 (Ind C-3), 23.8 (Ind 3-(CH_3_)_2_). ^15^N NMR (71 MHz, CDCl_3_): *δ*_N_ ppm –68.0 (Ind N-1). IR (neat, *ν_max_*, cm^−1^): 3062, 3037, 3023 (C-H_aromatic_), 2975, 2966, 2929, 2867 (C-H_aliphatic_), 2221 (C≡N), 1626, 1598, 1508, 1455, 1441, 1412, 1365, 1211, 1121, 1015, 965, 895, 863, 816, 744 (C=C, C=N, C-N, =C-H). HRMS (ESI-TOF): found: [M + H]^+^ 399.1856; molecular formula C_29_H_23_N_2_ requires [M + H]^+^ 399.1856.

##### 2-[(*E*)-2-(4-Methoxyphenyl)ethenyl]-3,3-dimethyl-5-(naphthalen-2-yl)-3*H*-indole (**9**)

The Suzuki cross-coupling was performed with 2-naphthylboronic acid (250 mg, 1.45 mmol), and the reaction time was 30 min. The Knoevenagel condensation was performed with 4-methoxybenzaldehyde (197 mg, 1.45 mmol), and the reaction time was 40 min. The residue was purified by column chromatography [ethyl acetate/toluene, 1:18 → acetone/*n*-hexane, 1:12 (*v*/*v*)], and the obtained product was washed with EtOH and dried to afford compound **9** (121 mg, overall yield 27%). M.p. 185–187 °C. ^1^H NMR (700 MHz, CDCl_3_): *δ*_H_ ppm 8.07 (s, 1H, Naph 1-H), 7.94–7.92 (m, 1H, Naph 4-H), 7.92–7.90 (m, 1H, Naph 8-H), 7.87 (d, *J* = 8.0 Hz, 1H, Naph 5-H), 7.79 (dd, *J* = 8.4, 1.8 Hz, 1H, Naph 3-H), 7.73 (d, *J* = 16.4 Hz, 1H, Ind-CH=CH-Ph), 7.72–7.70 (m, 2H, Ind 6-H, Ind 7-H), 7.66 (s, 1H, Ind 4-H), 7.58 (d, *J* = 8.7 Hz, 2H, Ph 2,6-H), 7.51 (t, *J* = 7.3 Hz, 1H, Naph 7-H), 7.48 (t, *J* = 7.3 Hz, 1H, Naph 6-H), 6.98 (d, *J* = 16.3 Hz, 1H, Ind-CH=CH-Ph), 6.94 (d, *J* = 8.7 Hz, 2H, Ph 3,5-H), 3.86 (s, 3H, -OCH_3_), 1.54 (s, 6H, Ind 3-(CH_3_)_2_). ^13^C NMR (176 MHz, CDCl_3_): *δ*_C_ ppm 183.9 (Ind C-2), 160.8 (Ph C-4), 153.6 (Ind C-7a), 147.3 (Ind C-3a), 138.8 (Naph C-2), 138.5 (Ind C-5), 137.8 (Ind-CH=CH-Ph), 133.7 (Naph C-8a), 132.5 (Naph C-4a), 129.1 (Ph C-2,6), 128.9 (Ph C-1), 128.4 (Naph C-4), 128.2 (Naph C-8), 127.7 (Naph C-5), 127.3 (Ind C-6), 126.3 (Naph C-7), 125.9 (Naph C-6), 125.7 (Naph C-1 and Naph C-3), 120.6 (Ind C-7), 120.2 (Ind C-4), 117.5 (Ind-CH=CH-Ph), 114.4 (Ph C-3,5), 55.4 (-OCH_3_), 52.8 (Ind C-3), 24.1 (Ind 3-(CH_3_)_2_). ^15^N NMR (71 MHz, CDCl_3_): *δ*_N_ ppm –77.7 (Ind N-1). IR (neat, *ν_max_*, cm^−1^): 3053, 3034 (C-H_aromatic_), 2989, 2958, 2932, 2904, 2864, 2832 (C-H_aliphatic_), 1625, 1598, 1511, 1454, 1247, 1174, 1030, 953, 857, 817, 750 (C=C, C=N, C-N, C-O, =C-H). HRMS (ESI-TOF): found: [M + H]^+^ 404.2009; molecular formula C_29_H_26_NO requires [M + H]^+^ 404.2009.

##### 4-{(*E*)-2-[3,3-Dimethyl-5-(naphthalen-2-yl)-3*H*-indol-2-yl]ethenyl}-*N*,*N*-dimethylaniline (**10**)

The Suzuki cross-coupling was performed with 2-naphthylboronic acid (250 mg, 1.45 mmol), and the reaction time was 30 min. The Knoevenagel condensation was performed with 4-(dimethylamino)benzaldehyde (217 mg, 1.45 mmol), and the reaction time was 120 min. The residue was purified by column chromatography [ethyl acetate/toluene, 1:18 → acetone/*n*-hexane, 1:12 (*v*/*v*)], and the obtained product was washed with EtOH and dried to afford compound **10** (111 mg, overall yield 24%). M.p. 250–252 °C. ^1^H NMR (700 MHz, CDCl_3_): *δ*_H_ ppm 8.07 (s, 1H, Naph 1-H), 7.94–7.92 (m, 1H, Naph 4-H), 7.92–7.90 (m, 1H, Naph 8-H), 7.87 (d, *J* = 7.9 Hz, 1H, Naph 5-H), 7.79 (dd, *J* = 8.4, 1.7 Hz, 1H, Naph 3-H), 7.72 (d, *J* = 16.2 Hz, 1H, Ind-CH=CH-Ph), 7.69–7.68 (m, 2H, Ind 6-H, Ind 7-H), 7.64 (s, 1H, Ind 4-H), 7.53 (d, *J* = 8.8 Hz, 2H, Ph 2,6-H), 7.52–7.49 (m, 1H, Naph 7-H), 7.48 (t, *J* = 7.3 Hz, 1H, Naph 6-H), 6.90 (d, *J* = 16.2 Hz, 1H, Ind-CH=CH-Ph), 6.72 (d, *J* = 8.7 Hz, 2H, Ph 3,5-H), 3.03 (s, 6H, -N(CH_3_)_2_), 1.54 (s, 6H, Ind 3-(CH_3_)_2_). ^13^C NMR (176 MHz, CDCl_3_): *δ*_C_ ppm 184.4 (Ind C-2), 153.9 (Ind C-7a), 151.2 (Ph C-1), 147.3 (Ind C-3a), 138.9 (Naph C-2), 138.7 (Ind-CH=CH-Ph), 138.0 (Ind C-5), 133.8 (Naph C-8a), 132.5 (Naph C-4a), 129.1 (Ph C-2,6), 128.4 (Naph C-4), 128.1 (Naph C-8), 127.6 (Naph C-5), 127.2 (Ind C-6), 126.3 (Naph C-7), 125.79 (Naph C-6), 125.74 (Naph C-3), 125.6 (Naph C-1), 124.1 (Ph C-4), 120.3 (Ind C-7), 120.1 (Ind C-4), 114.9 (Ind-CH=CH-Ph), 112.1 (Ph C-3,5), 52.6 (Ind C-3), 40.2 (-N(CH_3_)_2_), 24.3 (Ind 3-(CH_3_)_2_). ^15^N NMR (71 MHz, CDCl_3_): *δ*_N_ ppm –329.8 (N(CH_3_)_2_), –82.6 (Ind N-1). IR (neat, *ν_max_*, cm^−1^): 3048, 3024 (C-H_aromatic_), 2959, 2922, 2897, 2865, 2837 (C-H_aliphatic_), 1599, 1522, 1504, 1451, 1356, 1186, 1111, 973, 818, 810, 746 (C=C, C=N, C-N, C=C-H). HRMS (ESI-TOF): found: [M + H]^+^ 417.2325; molecular formula C_30_H_29_N_2_ requires [M + H]^+^ 417.2325.

##### 3,3-Dimethyl-5-(phenanthren-9-yl)-2-{(*E*)-2-[4-(trifluoromethyl)phenyl]ethenyl}-3*H*-indole (**11**)

The Suzuki cross-coupling was performed with 9-phenanthracenylboronic acid (322 mg, 1.45 mmol), and the reaction time was 30 min. The Knoevenagel condensation was performed with 4-(trifluoromethyl)benzaldehyde (252 mg, 1.45 mmol), and the reaction time was 20 min. The residue was purified by column chromatography [ethyl acetate/toluene, 1:18 → acetone/*n*-hexane, 1:14 (*v*/*v*)], and the obtained product was washed with EtOH and dried to afford compound **11** as a yellow powder (170 mg, overall yield 31%). M.p. 90–92 °C. ^1^H NMR (400 MHz, CDCl_3_): *δ*_H_ ppm 8.79 (d, *J* = 8.4 Hz, 1H, Phen 5-H), 8.73 (d, *J* = 8.2 Hz, 1H, Phen 4-H), 7.97 (d, *J* = 8.3 Hz, 1H, Phen 8-H), 7.91 (d, *J* = 7.8 Hz, 1H, Phen 1-H), 7.82–7.75 (m, 2H, Ind 7-H, Ind-CH=CH-Ph), 7.74 (s, 1H, Phen 10-H), 7.73 (d, *J* = 8.5 Hz, 2H, Ph 2,6-H), 7.70–7.60 (m, 5H, Phen 2-H, Phen 3-H, Phen 6-H, Ph 3,5-H), 7.58–7.53 (m, 2H, Ind 6-H, Phen 7-H), 7.52 (s, 1H, Ind 4-H), 7.20 (d, *J* = 16.4 Hz, 1H, Ind-CH=CH-Ph), 1.55 (s, 6H, Ind 3-(CH_3_)_2_). ^13^C NMR (101 MHz, CDCl_3_): *δ*_C_ ppm 183.0 (Ind C-2), 153.1 (Ind C-7a), 146.8 (Ind C-3a), 139.5 (Ph C-1), 138.8 (Ind C-5), 138.7 (Phen C-9), 136.2 (Ind-CH=CH-Ph), 131.5 (Phen C-10a), 131.2 (Phen C-8a), 130.8 (q, ^2^*J*_C,F_ = 32.6 Hz, Ph C-4), 130.7 (Phen C-4b), 129.99 (Ind C-6), 129.96 (Phen C-4a), 128.7 (Phen C-1), 127.7 (Phen C-10), 127.6 (Ph C-2,6), 126.9 (Phen C-8), 126.87 (Phen C-2), 126.7 (Phen C-3), 126.6 (Phen C-7), 126.5 (Phen C-6), 125.9 (q, ^3^*J*_C,F_ = 3.7 Hz, Ph C-3,5), 124.0 (q, ^1^*J*_C,F_ = 272.1 Hz, -CF_3_), 123.0 (Ind C-4 and Phen C-5), 122.6 (Phen C-4), 122.2 (Ind-CH=CH-Ph), 120.6 (Ind C-7), 53.0 (Ind C-3), 23.8 (Ind 3-(CH_3_)_2_). ^15^N NMR (41 MHz, CDCl_3_): *δ*_N_ ppm –69.8 (Ind N-1). ^19^F NMR (376 MHz, CDCl_3_): *δ*_F_ ppm –62.6 (s, CF_3_). IR (KBr, *ν_max_*, cm^−1^): 3073, 3053, 3018 (C-H_aromatic_), 2964, 2928, 2863 (C-H_aliphatic_), 1613, 1491, 1461, 1415, 1323, 1166, 1124, 1066, 1015, 971, 825, 748, 725 (C=C, C=N, CF_3_, C-N, =C-H). HRMS (ESI-TOF): found: [M + H]^+^ 492.1936; molecular formula C_33_H_25_F_3_N requires [M + H]^+^ 492.1934.

##### 4-{(*E*)-2-[3,3-Dimethyl-5-(phenanthren-9-yl)-3*H*-indol-2-yl]ethenyl}benzonitrile (**12**)

The Suzuki cross-coupling was performed with 9-phenanthracenylboronic acid (322 mg, 1.45 mmol), and the reaction time was 30 min. The Knoevenagel condensation was performed with 4-cyanobenzaldehyde (190 mg, 1.45 mmol), and the reaction time was 20 min. The residue was purified by column chromatography [ethyl acetate/toluene, 1:18 → acetone/*n*-hexane, 1:14 (*v*/*v*)], and the obtained product was washed with EtOH and dried to afford compound **12** as a yellow powder (145 mg, overall yield 29%). M.p. 217–219 °C. ^1^H NMR (400 MHz, CDCl_3_): *δ*_H_ ppm 8.78 (d, *J* = 8.3 Hz, 1H, Phen 5-H), 8.72 (d, *J* = 8.1 Hz, 1H, Phen 4-H), 7.96 (d, *J* = 8.2 Hz, 1H, Phen 8-H), 7.90 (d, *J* = 7.7 Hz, 1H, Phen 1-H), 7.82–7.74 (m, 2H, Ind 7-H, Ind-CH=CH-Ph), 7.73 (s, 1H, Phen 10-H), 7.70–7.65 (m, 6H, Ph 2,6-H, Ph 3,5-H, Phen 3-H, Phen 6-H), 7.62 (t, *J* = 7.3 Hz, 1H, Phen 2-H), 7.58–7.53 (m, 2H, Ind 6-H, Phen 7-H), 7.52 (s, 1H, Ind 4-H), 7.20 (d, *J* = 16.3 Hz, 1H, Ind-CH=CH-Ph), 1.54 (s, 6H, Ind 3-(CH_3_)_2_). ^13^C NMR (101 MHz, CDCl_3_): *δ*_C_ ppm 182.7 (Ind C-2), 153.1 (Ind C-7a), 146.7 (Ind C-3a), 140.4 (Ph C-4), 139.0 (Ind C-5), 138.6 (Phen C-9), 135.6 (Ind-CH=CH-Ph), 132.6 (Ph C-2,6), 131.5 (Phen C-10a), 131.1 (Phen C-8a), 130.7 (Phen C-4b), 130.0 (Ind C-6), 129.9 (Phen C-4a), 128.6 (Phen C-1), 127.8 (Ph C-3,5), 127.7 (Phen C-10), 126.9 (Phen C-8), 126.8 (Phen C-2), 126.7 (Phen C-3), 126.6 (Phen C-7), 126.5 (Phen C-6), 123.1 (Ind-CH=CH-Ph), 123.0 (Ind C-4 and Phen C-5), 122.6 (Phen C-4), 120.8 (Ind C-7), 118.7 (-CN), 112.2 (Ph C-1), 53.0 (Ind C-3), 23.7 (Ind 3-(CH_3_)_2_). ^15^N NMR (41 MHz, CDCl_3_): *δ*_N_ ppm –67.3 (Ind N-1). IR (KBr, *ν_max_*, cm^−1^): 3071, 3055, 3010 (C-H_aromatic_), 2969, 2928, 2866 (C-H_aliphatic_), 2223 (C≡N), 1625, 1600, 1516, 1495, 1461, 1448, 1343, 1212, 1109, 975, 953, 897, 869, 823, 750, 728, 549 (C=C, C=N, C-N, =C-H). HRMS (ESI-TOF): found: [M + H]^+^ 449.2012; molecular formula C_33_H_25_N_2_ requires [M + H]^+^ 449.2012.

##### 2-[(*E*)-2-(4-Methoxyphenyl)ethenyl]-3,3-dimethyl-5-(phenanthren-9-yl)-3*H*-indole (**13**)

The Suzuki cross-coupling was performed with 9-phenanthracenylboronic acid (322 mg, 1.45 mmol), and the reaction time was 30 min. The Knoevenagel condensation was performed with 4-methoxybenzaldehyde (197 mg, 1.45 mmol), and the reaction time was 40 min. The residue was purified by column chromatography [ethyl acetate/toluene, 1:18 → acetone/*n*-hexane, 1:12 (*v*/*v*)], and the obtained product was washed with EtOH and dried to afford compound **13** as a yellow powder (132 mg, overall yield 26%). M.p. 166–168 °C. ^1^H NMR (400 MHz, CDCl_3_): *δ*_H_ ppm 8.79 (d, *J* = 8.3 Hz, 1H, Phen 5-H), 8.73 (d, *J* = 8.2 Hz, 1H, Phen 4-H), 7.98 (d, *J* = 8.2 Hz, 1H, Phen 8-H), 7.91 (d, *J* = 7.7 Hz, 1H, Phen 1-H), 7.78–7.72 (m, 3H, Ind 7-H, Ind-CH=CH-Ph, Phen 10-H), 7.71–7.61 (m, 3H, Phen 2-H, Phen 3-H, Phen 6-H), 7.59 (d, *J* = 8.8 Hz, 2H, Ph 2,6-H), 7.57–7.50 (m, 2H, Ind 6-H, Phen 7-H), 7.49 (s, 1H, Ind 4-H), 7.00 (d, *J* = 16.3 Hz, 1H, Ind-CH=CH-Ph), 6.95 (d, *J* = 8.4 Hz, 2H, Ph 3,5-H), 3.86 (s, 3H, -OCH_3_), 1.54 (s, 6H, Ind 3-(CH_3_)_2_). ^13^C NMR (101 MHz, CDCl_3_): *δ*_C_ ppm 183.9 (Ind C-2), 160.7 (Ph C-4), 153.5 (Ind C-7a), 146.7 (Ind C-3a), 138.9 (Phen C-9), 138.0 (Ind C-5), 137.8 (Ind-CH=CH-Ph), 131.6 (Phen C-10a), 131.3 (Phen C-8a), 130.7 (Phen C-4b), 129.9 (Phen C-4a), 129.8 (Ind C-6), 129.1 (Ind C-6), 128.9 (Ph C-1), 128.6 (Phen C-1), 127.6 (Phen C-10), 127.0 (Phen C-8), 126.9 (Phen C-2), 126.6 (Phen C-3), 126.5 (Phen C-7), 126.46 (Phen C-6), 122.9 (Ind C-4 and Phen C-5), 122.5 (Phen C-4), 120.1 (Ind C-7), 117.6 (Ind-CH=CH-Ph), 114.4 (Ph C-3,5), 55.4 (-OCH_3_), 52.8 (Ind C-3), 24.1 (Ind 3-(CH_3_)_2_). ^15^N NMR (41 MHz, CDCl_3_): *δ*_N_ ppm –77.4 (Ind N-1). IR (KBr, *ν_max_*, cm^−1^): 3071, 3033, 3009 (C-H_aromatic_), 2957, 2928, 2860 2835 (C-H_aliphatic_), 1602, 1512, 1461, 1421, 1303, 1253, 1173, 1110, 1031, 971, 823, 748, 726 (C=C, C=N, C-N, C-O, =C-H). HRMS (ESI-TOF): found: [M + H]^+^ 454.2165; molecular formula C_33_H_28_NO requires [M + H]^+^ 454.2165.

##### 3,3-Dimethyl-5-(pyren-1-yl)-2-{(*E*)-2-[4-(trifluoromethyl)phenyl]ethenyl}-3*H*-indole (**14**)

The Suzuki cross-coupling was performed with 1-pyrenylboronic acid (357 mg, 1.45 mmol), and the reaction time was 40 min. The Knoevenagel condensation was performed with 4-(trifluoromethyl)benzaldehyde (252 mg, 1.45 mmol), and the reaction time was 20 min. The residue was purified by column chromatography [ethyl acetate/toluene, 1:18 → acetone/*n*-hexane, 1:14 (*v*/*v*)], and the obtained product was washed with EtOH and dried to afford compound **14** as a yellow powder (190 mg, overall yield 33%). M.p. 210–211 °C. ^1^H NMR (700 MHz, CDCl_3_): *δ*_H_ ppm 8.23 (d, *J* = 7.6 Hz, 1H, Pyren 3-H), 8.22 (d, *J* = 7.5 Hz, 1H, Pyren 10-H), 8.20 (d, *J* = 7.6 Hz, 1H, Pyren 8-H), 8.17 (d, *J* = 7.5 Hz, 1H, Pyren 6-H), 8.12–8.08 (m, 2H, Pyren 4-H, Pyren 5-H), 8.04 (d, *J* = 7.5 Hz, 1H, Pyren 9-H), 8.03–8.02 (m, 1H, Pyren 2-H), 8.02–8.00 (m, 1H, Pyren 7-H), 7.83 (d, *J* = 7.8 Hz, 1H, Ind 7-H), 7.79 (d, *J* = 16.4 Hz, 1H, Ind-CH=CH-Ph), 7.73 (d, *J* = 8.0 Hz, 2H, Ph 2,6-H), 7.67 (d, *J* = 8.1 Hz, 2H, Ph 3,5-H), 7.63 (d, *J* = 7.8 Hz, 1H, Ind 6-H), 7.60 (s, 1H, Ind 4-H), 7.21 (d, *J* = 16.4 Hz, 1H, Ind-CH=CH-Ph), 1.58 (s, 6H, Ind 3-(CH_3_)_2_). ^13^C NMR (176 MHz, CDCl_3_): *δ*_C_ ppm 183.1 (Ind C-2), 153.1 (Ind C-7a), 146.9 (Ind C-3a), 139.5 (Ph C-1), 139.2 (Ind C-5), 137.7 (Pyren C-1), 136.2 (Ind-CH=CH-Ph), 131.5 (Pyren C-5a), 131.0 (Pyren C-8a), 130.8 (q, ^2^*J*_C,F_ = 32.5 Hz, Ph C-4), 130.6 (Pyren C-3a and Ind C-6), 128.6 (Pyren C-10a), 127.8 (Pyren C-2), 127.6 (Ph C-2,6 and Pyren C-9), 127.5 (Pyren C-5), 127.4 (Pyren C-4), 126.1 (Pyren C-7), 125.9 (q, ^3^*J*_C,F_ = 3.7 Hz, Ph C-3), 125.2 (Pyren C-10 and Pyren C-6), 125.1 (Pyren C-10b), 124.93 (Pyren C-10c), 124.88 (Pyren C-8), 124.7 (Pyren C-3), 124.0 (q, ^1^*J*_C,F_ = 272.0 Hz, -CF_3_), 123.6 (Ind C-4), 122.2 (Ind-CH=CH-Ph), 120.7 (Ind C-7), 53.0 (Ind C-3), 23.8 (Ind 3-(CH_3_)_2_). ^15^N NMR (71 MHz, CDCl_3_): *δ*_N_ ppm –70.1 (Ind N-1). ^19^F NMR (376 MHz, CDCl_3_): *δ*_F_ ppm –62.7 (s, CF_3_). IR (KBr, *ν_max_*, cm^−1^): 3040, 3020, 3009 (C-H_aromatic_), 2981, 2965, 2931, 2865 (C-H_aliphatic_), 1626, 1611, 1601, 1577, 1513, 1453, 1414, 1322, 1171, 1100, 1057, 1014, 973, 952, 852, 838, 821, 766, 721 (C=C, C=N, C-N, CF_3_, =C-H). HRMS (ESI-TOF): found: [M + H]^+^ 516.1934; molecular formula C_35_H_25_F_3_N requires [M + H]^+^ 516.1934.

##### 4-{(*E*)-2-[3,3-Dimethyl-5-(pyren-1-yl)-3*H*-indol-2-yl]ethenyl}benzonitrile (**15**)

The Suzuki cross-coupling was performed with 1-pyrenylboronic acid (357 mg, 1.45 mmol), and the reaction time was 40 min. The Knoevenagel condensation was performed with 4-cyanobenzaldehyde (190 mg, 1.45 mmol), and the reaction time was 20 min. The residue was purified by column chromatography [ethyl acetate/toluene, 1:18 → acetone/*n*-hexane, 1:12 (*v*/*v*)], and the obtained product was washed with EtOH and dried to afford compound **15** as a yellow powder (159 mg, overall yield 30%). M.p. 244–246 °C. ^1^H NMR (700 MHz, CDCl_3_): *δ*_H_ ppm 8.24 (d, *J* = 7.7 Hz, 1H, Pyren 3-H), 8.23–8.21 (m, 1H, Pyren 10-H), 8.21 (d, *J* = 6.1 Hz, 1H, Pyren 6-H), 8.18 (d, *J* = 7.5 Hz, 1H, Pyren 8-H), 8.13–8.09 (m, 2H, Pyren 4-H, Pyren 5-H), 8.05 (d, *J* = 7.6 Hz, 1H, Pyren 9-H), 8.04–8.03 (m, 1H, Pyren 7-H), 8.03–8.01 (m, 1H, Pyren 2-H), 7.84 (d, *J* = 7.8 Hz, 1H, Ind 7-H), 7.77 (d, *J* = 16.4 Hz, 1H, Ind-CH=CH-Ph), 7.71 (d, *J* = 8.6 Hz, 2H, Ph 3,5-H), 7.70 (d, *J* = 8.6 Hz, 2H, Ph 2,6-H), 7.65 (dd, *J* = 7.8, 1.3 Hz, 1H, Ind 6-H), 7.61 (d, *J* = 1.3 Hz, 1H, Ind 4-H), 7.22 (d, *J* = 16.4 Hz, 1H, Ind-CH=CH-Ph), 1.57 (s, 6H, Ind 3-(CH_3_)_2_). ^13^C NMR (176 MHz, CDCl_3_): *δ*_C_ ppm 182.7 (Ind C-2), 153.0 (Ind C-7a), 146.9 (Ind C-3a), 140.4 (Ph C-4), 139.5 (Ind C-5), 137.6 (Pyren C-1), 135.6 (Ind-CH=CH-Ph), 132.7 (Ph C-2,6), 131.5 (Pyren C-5a), 131.0 (Pyren C-8a), 130.7 (Pyren C-3a and Ind C-6), 128.6 (Pyren C-10a), 127.8 (Ph C-3,5), 127.7 (Pyren C-2), 127.6 (Pyren C-9), 127.5 (Pyren C-5), 127.4 (Pyren C-4), 126.1 (Pyren C-7), 125.23 (Pyren C-6), 125.17 (Pyren C-10), 125.0 (Pyren C-10b), 124.9 (Pyren C-8 and Pyren C-10c), 124.7 (Pyren C-3), 123.6 (Ind C-4), 123.2 (Ind-CH=CH-Ph), 120.8 (Ind C-7), 118.7 (-CN), 112.3 (Ph C-1), 53.0 (Ind C-3), 23.8 (Ind 3-(CH_3_)_2_). ^15^N NMR (71 MHz, CDCl_3_): *δ*_N_ ppm –67.9 (Ind N-1). IR (KBr, *ν_max_*, cm^−1^): 3038, 3011 (C-H_aromatic_), 2963, 2929, 2864 (C-H_aliphatic_), 2220 (C≡N), 1657, 1626, 1599, 1507, 1452, 1410, 1344, 1286, 1213, 1186, 1108, 974, 951, 852, 837, 819, 763, 724, 682 (C=C, C=N, C-N, =C-H). HRMS (ESI-TOF): found: [M + H]^+^ 473.2012; molecular formula C_35_H_25_N_2_ requires [M + H]^+^ 473.2012.

##### 2-[(*E*)-2-(4-Methoxyphenyl)ethenyl]-3,3-dimethyl-5-(pyren-1-yl)-3*H*-indole (**16**)

The Suzuki cross-coupling was performed with 1-pyrenylboronic acid (357 mg, 1.45 mmol), and the reaction time was 40 min. The Knoevenagel condensation was performed with 4-methoxybenzaldehyde (197 mg, 1.45 mmol), and the reaction time was 40 min. The residue was purified by column chromatography [ethyl acetate/toluene, 1:18 → acetone/*n*-hexane, 1:12 (*v*/*v*)], and the obtained product was washed with EtOH and dried to afford compound **16** as a yellow powder (122 mg, overall yield 21%). M.p. 197–199 °C. ^1^H NMR (700 MHz, CDCl_3_): *δ*_H_ ppm 8.24 (d, *J* = 7.6 Hz, 1H, Pyren 10-H), 8.22 (d, *J* = 7.7 Hz, 1H, Pyren 3-H), 8.20 (d, *J* = 7.5 Hz, 1H, Pyren 6-H), 8.17 (d, *J* = 7.5 Hz, 1H, Pyren 8-H), 8.12–8.08 (m, 2H, Pyren 4-H, Pyren 5-H), 8.06–8.00 (m, 3H, Pyren 9-H, Pyren 2-H, Pyren 7-H), 7.79 (d, *J* = 7.8 Hz, 1H, Ind 7-H), 7.76 (d, *J* = 16.4 Hz, 1H, Ind-CH=CH-Ph), 7.62–7.57 (m, 4H, Ind 6-H, Ind 4-H, Ph 2,6-H), 7.02 (d, *J* = 16.4 Hz, 1H, Ind-CH=CH-Ph), 6.95 (d, *J* = 8.6 Hz, 2H, Ph 3,5-H), 3.84 (s, 3H, -OCH_3_), 1.55 (s, 6H, Ind 3-(CH_3_)_2_). ^13^C NMR (176 MHz, CDCl_3_): *δ*_C_ ppm 184.0 (Ind C-2), 160.7 (Ph C-4), 153.4 (Ind C-7a), 146.8 (Ind C-3a), 138.4 (Ind C-5), 137.9 (Pyren C-1), 137.85 (Ind-CH=CH-Ph), 131.5 (Pyren C-5a), 131.0 (Pyren C-8a), 130.5 (Pyren C-3a), 130.4 (Ind C-6), 129.1 (Ph C-2,6), 128.8 (Ph C-1), 128.6 (Pyren C-10a), 127.8 (Pyren C-2), 127.5 (Pyren C-9), 127.4 (Pyren C-4 and Pyren C-5), 126.0 (Pyren C-7), 125.3 (Pyren C-10), 125.1 (Pyren C-6), 125.0 (Pyren C-10b), 124.9 (Pyren C-10c), 124.8 (Pyren C-8), 124.7 (Pyren C-3), 123.4 (Ind C-4), 120.1 (Ind C-7), 117.6 (Ind-CH=CH-Ph), 114.4 (Ph C-3,5), 55.4 (-OCH_3_), 52.8 (Ind C-3), 24.1 (Ind 3-(CH_3_)_2_). ^15^N NMR (71 MHz, CDCl_3_): *δ*_N_ ppm –77.5 (Ind N-1). IR (KBr, *ν_max_*, cm^−1^): 3037 (C-H_aromatic_), 2958, 2927, 2906, 2861, 2834 (C-H_aliphatic_), 1796, 1600, 1573, 1511, 1455, 1303, 1248, 1171, 1109, 1030, 969, 837, 819, 720 (C=C, C=N, C-N, C-O, =C-H). HRMS (ESI-TOF): found: [M + H]^+^ 478.2165; molecular formula C_35_H_28_NO requires [M + H]^+^ 478.2165.

##### 4-{(*E*)-2-[3,3-Dimethyl-5-phenyl-3*H*-indol-2-yl]ethenyl}benzonitrile (**17**)

The Suzuki cross-coupling was performed with phenylboronic acid (177 mg, 1.45 mmol), and the reaction time was 30 min. The Knoevenagel condensation was performed with 4-cyanobenzaldehyde (190 mg, 1.45 mmol), and the reaction time was 20 min. The residue was purified by column chromatography [ethyl acetate/toluene, 1:18 → acetone/*n*-hexane, 1:14 (*v*/*v*)], and the obtained product was washed with EtOH and dried to afford compound **17** as a yellow powder (152 mg, overall yield 39%). M.p. 208–210 °C. ^1^H NMR (700 MHz, CDCl_3_): *δ*_H_ ppm 7.74–7.70 (m, 2H, Ind 7-H, Ind-CH=CH-Ph), 7.68 (s, 4H, Ph 2,6-H, Ph 3,5-H), 7.64–7.62 (m, 2H Ind 5-CPh 2,6-H), 7.60 (dd, *J* = 8.0, 1.8 Hz, 1H, Ind 6-H), 7.55 (d, *J* = 1.8 Hz, 1H, Ind 4-H), 7.46 (t, *J* = 7.7 Hz, 2H, Ind 5-CPh 3,5-H), 7.37–7.34 (m, 1H, Ind 5-CPh 4-H), 7.17 (d, *J* = 16.3 Hz, 1H, Ind-CH=CH-Ph), 1.52 (s, 6H, Ind 3-(CH_3_)_2_). ^13^C NMR (176 MHz, CDCl_3_): *δ*_C_ ppm 182.5 (Ind C-2), 153.1 (Ind C-7a), 147.2 (Ind C-3a), 141.1 (Ind 5-CPh C-1), 140.4 (Ph C-4), 139.6 (Ind C-5), 135.5 (Ind-CH=CH-Ph), 132.6 (Ph C-2,6), 128.8 (Ind 5-CPh C-3,5), 127.8 (Ph C-3,5), 127.3 (Ind 5-CPh C-4), 127.2 (Ind 5-CPh C-2,6 and Ind C-6), 123.1 (Ind-CH=CH-Ph), 121.2 (Ind C-7), 120.1 (Ind C-4), 118.7 (-CN), 112.2 (Ph C-1), 52.9 (Ind C-3), 23.7 (Ind 3-(CH_3_)_2_). ^15^N NMR (71 MHz, CDCl_3_): *δ*_N_ ppm –67.9 (Ind N-1). IR (neat, *ν_max_*, cm^−1^): 3058, 3035 (C-H_aromatic_), 2962, 2935, 2904, 2862 (C-H_aliphatic_), 2224 (C≡N), 1625, 1598, 1507, 1456, 1352, 1212, 1109, 977, 906, 835, 821, 775, 757, 697, 547 (C=C, C=N, C-N, =C-H). HRMS (ESI-TOF): found: [M + H]^+^ 349.1699; molecular formula C_25_H_21_N_2_ requires [M + H]^+^ 349.1699.

## 4. Conclusions

In conclusion, we developed a straightforward and rapid one-pot Fischer–Suzuki–Knoevenagel protocol for synthesizing novel fluorescent 5-aryl-2-styryl-3*H*-indole derivatives using commercially accessible 4-bromophenylhydrazine hydrochloride as the starting material. This method employs water, ethanol, and acetic acid as solvents, making it an environmentally conscious approach to synthesizing new fluorescent styryl dyes. The novel compounds were characterized using IR and advanced NMR spectroscopies, along with HRMS data. The synthesized 5-aryl-2-styryl-3*H*-indole derivatives exhibit large Stokes shift values, which generally increase with the size of the aryl substituents in most compounds. Furthermore, compounds featuring electron-withdrawing substituents show strong fluorescence and possess high quantum yields, making them interesting candidates for various biomedical and technical applications.

## Figures and Tables

**Table 1:** Screening of reaction conditions for Fischer synthesis of compound **2** *^a^*.

** 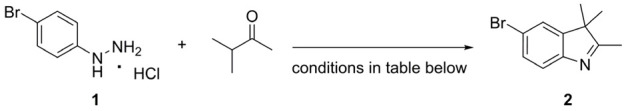 **					
Entry	Catalyst (mol%)	Solvent	Ketone (eq.)	Time (min)	Yield (%) *^b^*
1 *^c^*	H_2_SO_4_ (10)	EtOH	1.5	18 hours *^d^*	55
2	H_2_SO_4_ (10)	EtOH	1.5	10	58
3	-	AcOH	1.2	10	54
4 *^e^*	H_2_SO_4_ (10)	H_2_O	1.1	10	67
5	H_2_SO_4_ (10)	H_2_O	1.5	15	67
6 *^f^*	H_2_SO_4_ (10)	EtOH:H_2_O	1.5	10	55

*^a^* Unless otherwise specified, the reaction was performed under microwave heating at 100 °C. *^b^* Isolated yield. *^c^* Reflux under conventional heating. *^d^* After 10 minutes, the formation of the hydrazone intermediate was observed. *^e^* Optimal conditions. *^f^* Solvent mixture ratio of 3:1.

**Table 2:** Screening of Suzuki cross-coupling reaction conditions for the synthesis of compound **3** *^a^*.

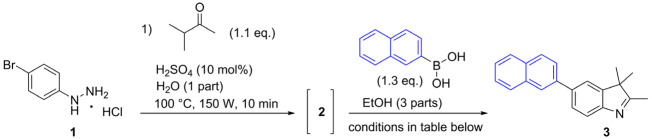						
Entry	Catalyst (mol%)	Base (eq.)	Ligand (eq.)	Temperature (°C)	Time (min)	Overall Yield (%) *^b^*
1	Pd(OAc)_2_ (10)	Cs_2_CO_3_ (3)	PPh_3_ (0.4)	80	40	45
2 *^c^*	Pd(OAc)_2_ (10)	Cs_2_CO_3_ (3)	PPh_3_ (0.4)	80	40	35
3 *^c^*	Pd(OAc)_2_ (10)	Cs_2_CO_3_ (3)	PPh_3_ (0.4)	80	2 h	48
4	Pd(OAc)_2_ (5)	Cs_2_CO_3_ (3)	PPh_3_ (0.4)	100	30	48
5	Pd(OAc)_2_ (5)	Cs_2_CO_3_ (2.5)	-	100	30	46
6 *^d^*	Pd(OAc)_2_ (5)	K_2_CO_3_ (2.5)	-	100	30	48
7 *^e^*	Pd(OAc)_2_ (5)	K_2_CO_3_ (2)	-	100	30	29

*^a^* Unless otherwise specified, the reaction was performed under microwave heating. *^b^* Isolated yield. *^c^* Conventional heating. *^d^* Optimal conditions. *^e^* 1.1 eq. of 2-naphthylboronic acid was used.

**Table 3:** Screening of Knoevenagel reaction conditions for the Fischer–Suzuki–Knoevenagel one-pot synthesis of compound **7** ^*a*^.

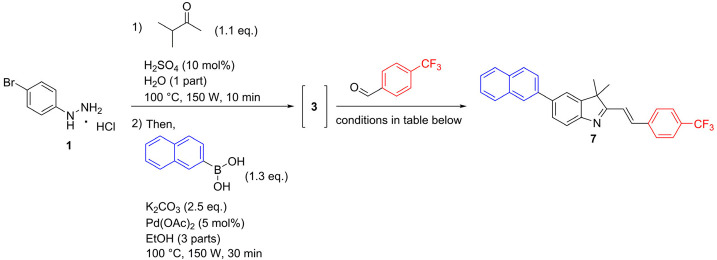				
Entry	Additive (eq.)	Aldehyde (eq.)	Time (min)	Overall Yield (%) *^b^*
1	-	1.5	60	Traces
2	Piperidine (0.5)	1.5	60	Traces
3	*L*-Proline (0.5)	1.5	60	Traces
4	AcOH (10)	1.3	60	Traces
5 *^c^*	AcOH *^d^*	1.3	20	29

*^a^* Optimization was conducted under microwave heating with a reaction temperature of 100 °C. ^*b*^ Isolated yield. *^c^* Optimal conditions. *^d^* The volumetrically equal amount of acetic acid, which forms a 4:3:1 mixture of AcOH:EtOH:H_2_O.

**Table 4:** Absorption and emission parameters for intermediates **3**–**6** and final compounds **7**–**17** in THF.

Entry	Compound	λ_abs_ (nm) *^a^*	ε × 10^3^ (M^−1^cm^−1^) *^b^*	λ_em_ (nm)	Stokes Shift (cm^−1^)	λ_ex_ *^c^* (nm)	Φ*_f_* (%)
1	**3**	304	24.5	351 367	4405 5647	320	<0.1
2	**4**	300	17.3	360 376	5556 6738	300	9.5
3	**5**	344	32.8	389 403	3363 4256	350	46.6
4	**6**	283	19.0	367	8088	320	3.2
5	**7**	368	35.6	490	6766	380	49.4
6	**8**	376	20.2	507	6872	380	71.5
7	**9**	370	44.7	464 491	5475 6660	380	0.2
8	**10**	412	49.5	538	5684	380	<0.1
9	**11**	355	26.2	485	7550	350	42.2
10	**12**	368	29.8	508	7489	350	67.9
11	**13**	361	31.3	448	5379	350	1.3
12	**14**	366	40.2	538	8735	380	70.1
13	**15**	371	38.6	552	8838	380	71.0
14	**16**	372	43.8	492	6557	380	5.4
15	**17**	369	33.4	491	6734	380	53.5

*^a^* The most bathochromically shifted absorption maximum value. ^*b*^ Concentration: 2.5 × 10^−4^ M. *^c^* Excitation wavelength for Φ*_f_* calculation.

## Data Availability

The data that support the findings of this study are available upon request.

## Supplementary Materials

The following supporting information can be downloaded at: https://www.mdpi.com/article/10.3390/molecules30122503/s1, Figure S1: Absorption spectra of **3**–**6** in THF; Figure S2: Emission spectra of **3**–**6** in THF; Figure S3: Absorption spectra of **7**–**17** in THF; Figure S4: Fluorescence emission spectra of **7**–**17** in THF; Figures S5–S67: ^1^H, ^13^C, ^1^H-^15^N HMBC NMR spectra, HRMS (ESI-TOF) spectral data of compounds **3**–**17**, and ^19^F NMR spectra of compounds **7**, **11**, and **14**.
