# Supplementary material for: Facile One-Pot Fischer–Suzuki–Knoevenagel Microwave-Assisted Synthesis of Fluorescent 5-Aryl-2-Styryl-3H-Indoles
Source: Molecules. 2025 Jun 7;30(12):2503. doi: 10.3390/molecules30122503 (PMC12196462; doi:10.3390/molecules30122503)
Supplement: Supplementary file 1 [file molecules-30-02503-s001.zip › molecules-3663302-supplementary.pdf]

SUPPORTING INFORMATION

Facile One-Pot Fischer–Suzuki–Knoevenagel Microwave-Assisted Synthesis of Fluorescent 5-Aryl-2-Styryl-3*H*-Indoles

Martynas Rojus Bartkus[1]*, Neringa Kleizienė[2], Aurimas Bieliauskas[2] and Algirdas Šačkus[1,2]*

[1] Department of Organic Chemistry, Kaunas University of Technology, Radvilėnų pl. 19, LT-50254 Kaunas, Lithuania

[2] Institute of Synthetic Chemistry, Kaunas University of Technology, K. Baršausko g. 59, LT-51423, Kaunas, Lithuania

* Corresponding authors: martynas.bartkus@ktu.lt (M. R. Bartkus), algirdas.sackus@ktu.lt (A. Šačkus)

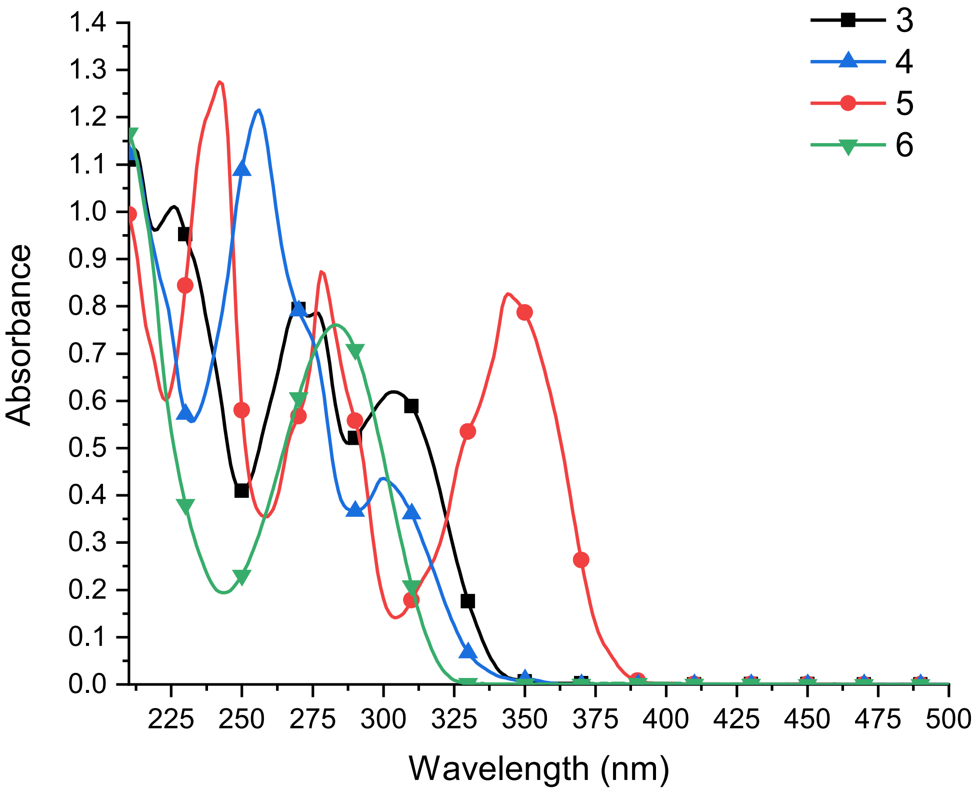

Figure S1. Absorption spectra of **3–6** in THF.

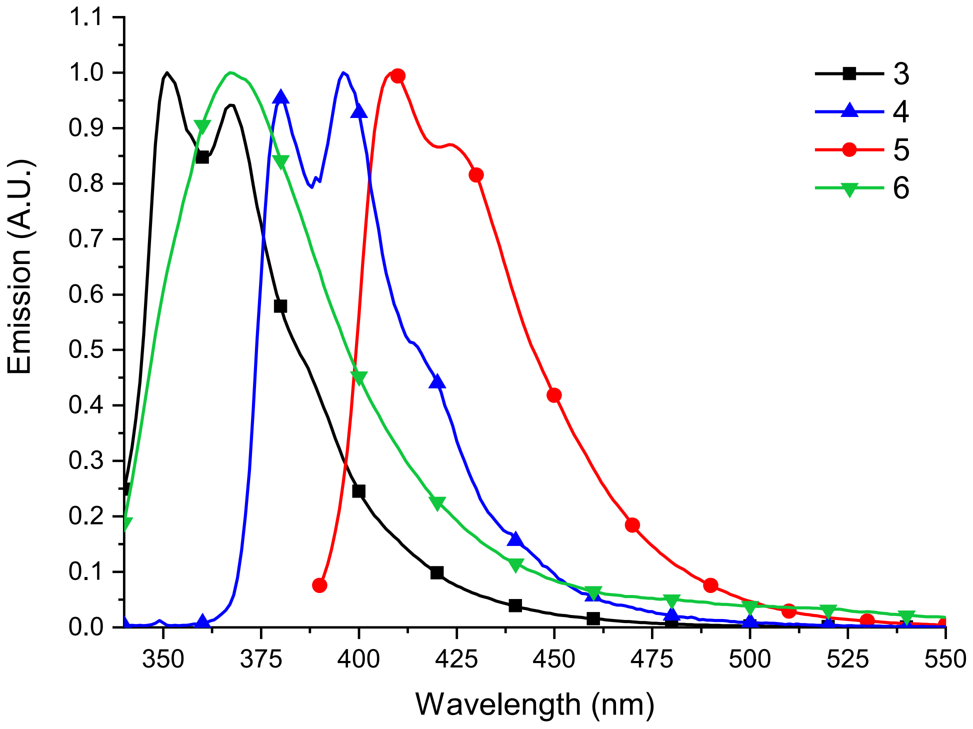

Figure S2. Emission spectra of **3–6** in THF.

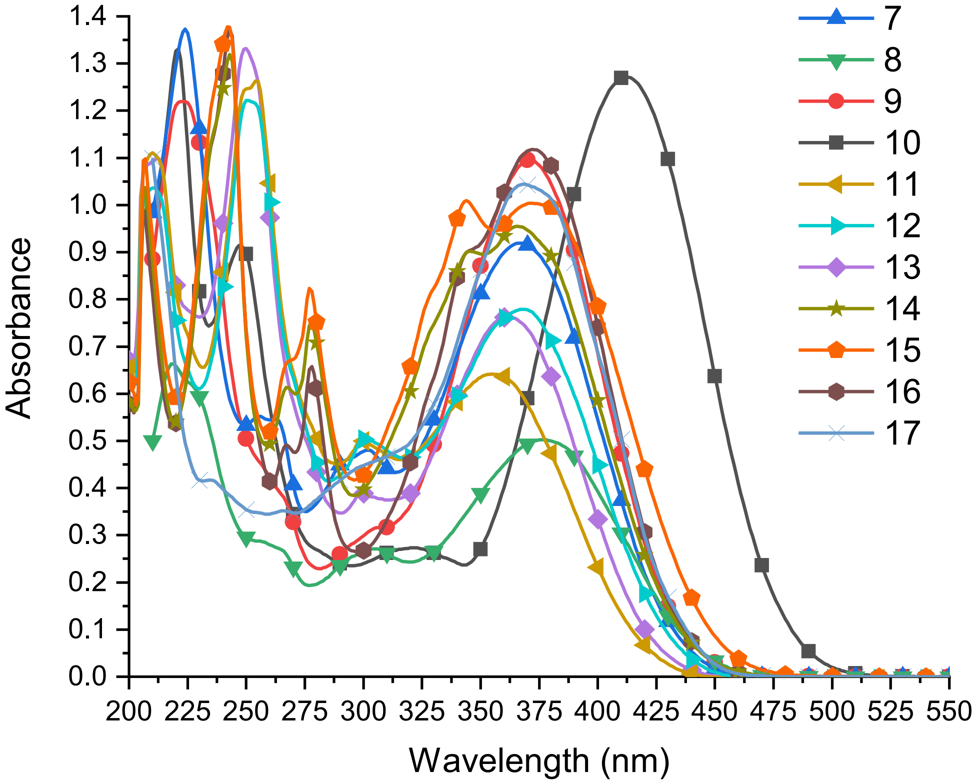

Figure S3. Absorption spectra of **7–17** in THF.

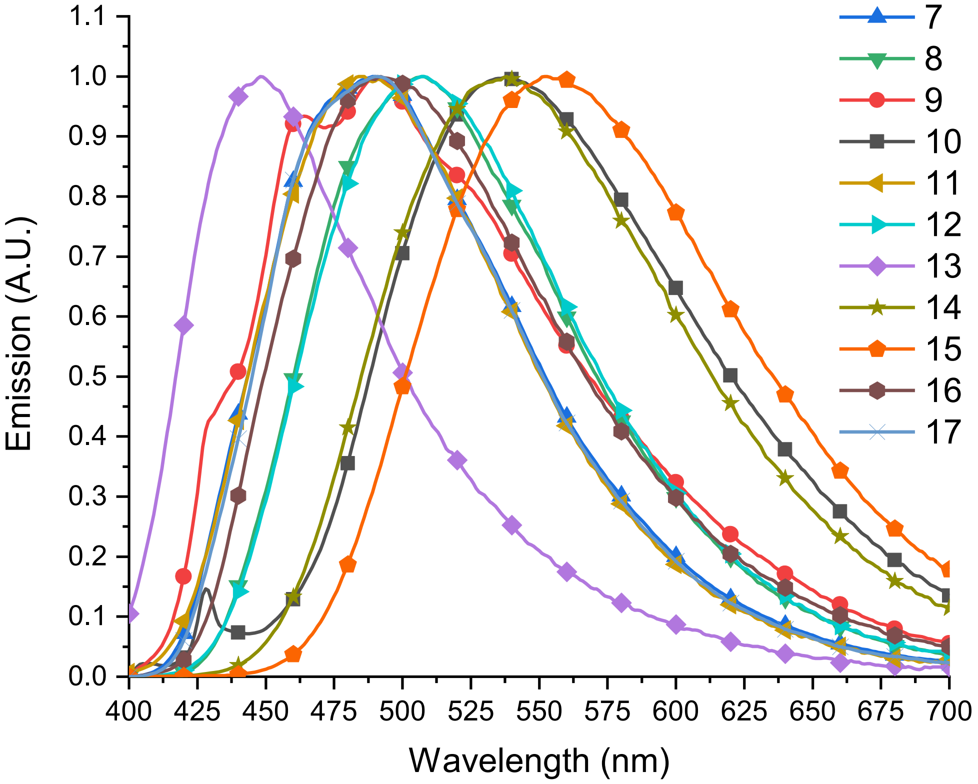

Figure S4. Fluorescence emission spectra of **7–17** in THF.

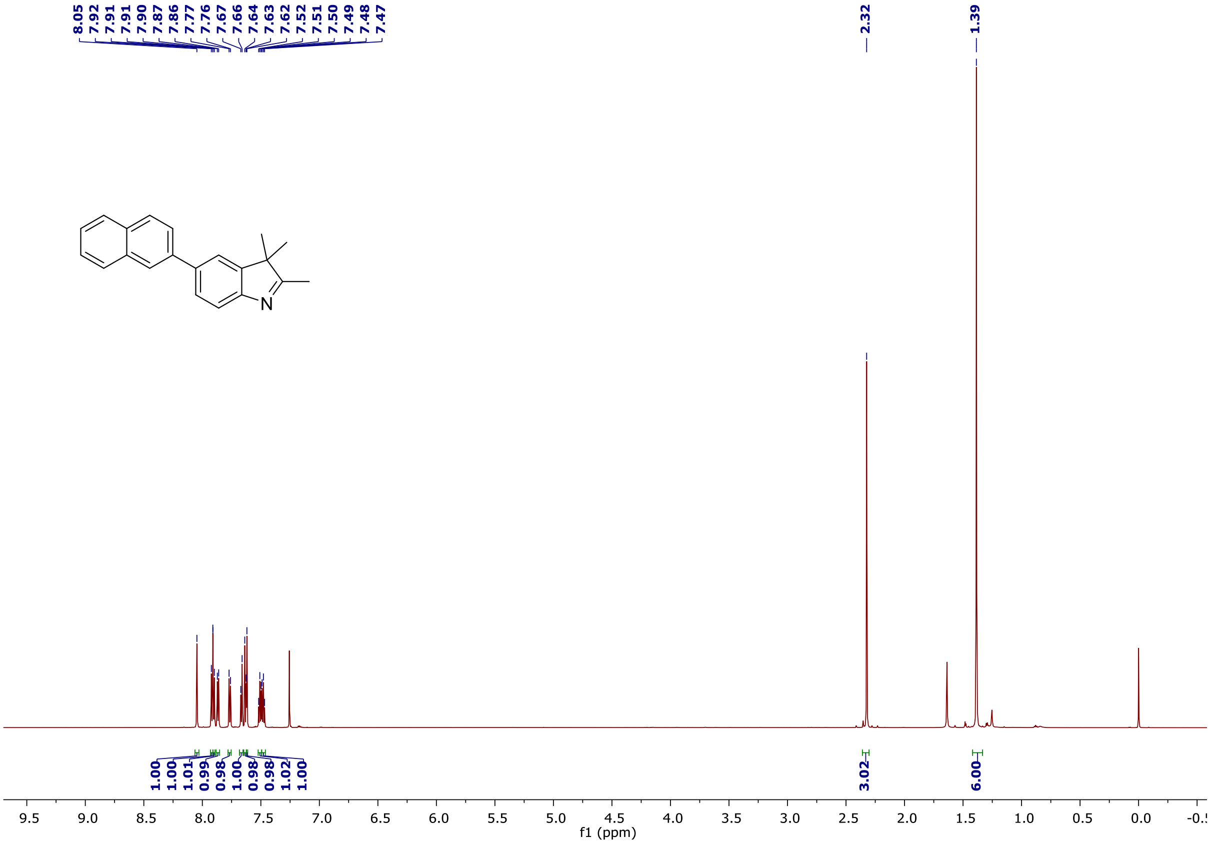

Figure S5. 2,3,3-Trimethyl-5-(naphthalen-2-yl)-3*H*-indole (**3**).^{1}H NMR spectrum (700 MHz, $CDCl_3$).

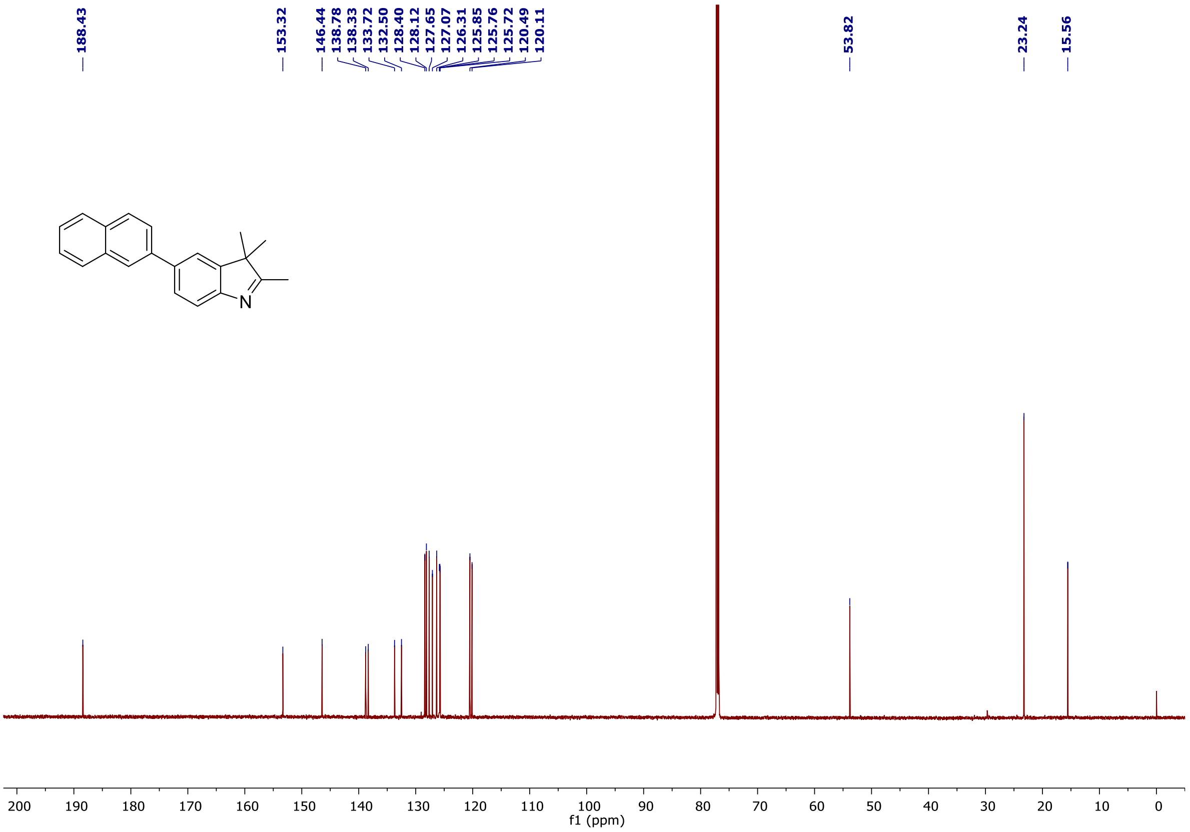

Figure S6. 2,3,3-Trimethyl-5-(naphthalen-2-yl)-3*H*-indole (**3**). ^{13}C NMR spectrum (176 MHz, $CDCl_3$).

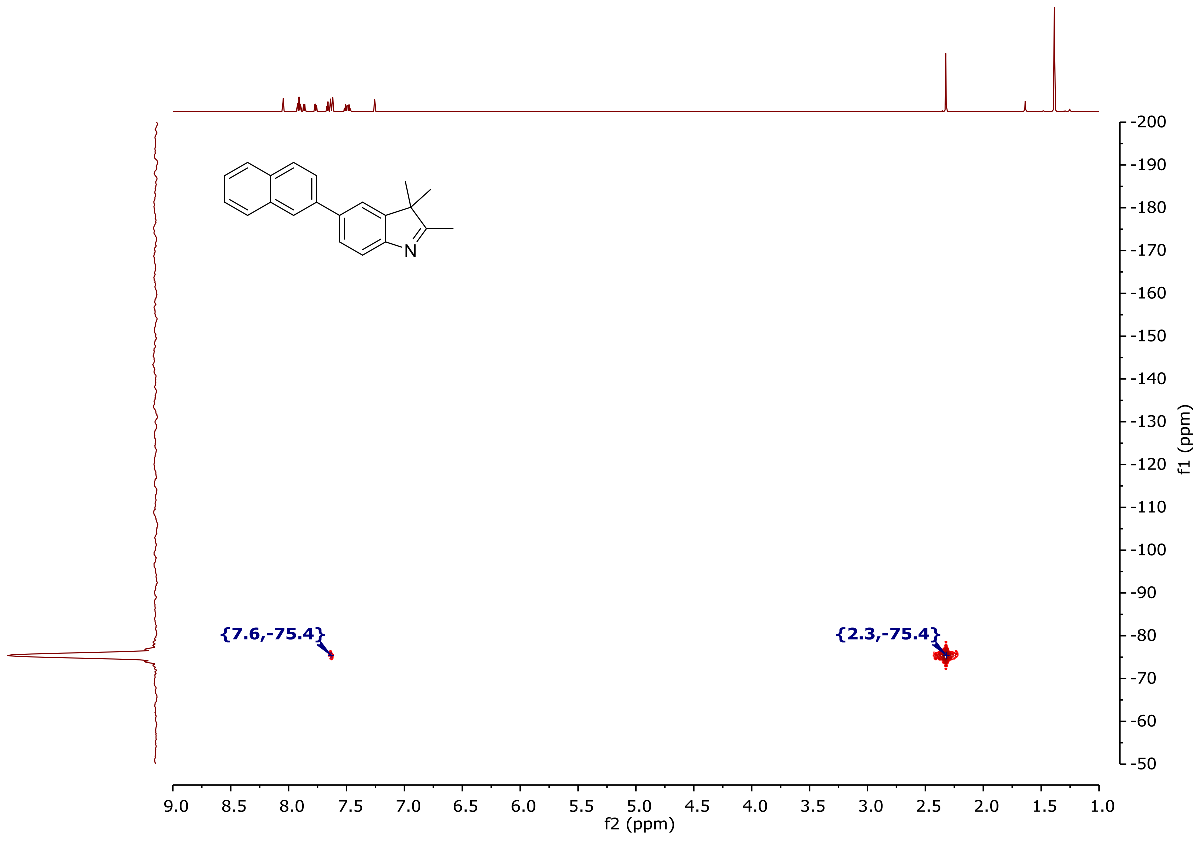

Figure S7. 2,3,3-Trimethyl-5-(naphthalen-2-yl)-3*H*-indole (**3**). ^{1}H-^{15}N HMBC NMR spectrum (71 MHz, $CDCl_3$).

Compound Spectrum SmartFormula Report

Analysis Info

Analysis Name	D:\Data\MRB-3.d	Acquisition Date	8/17/2021 11:38:25 AM
Method	DirectInfusion_TuneLow_pos.m	Operator	hplc
Sample Name	MRB-3	Instrument	micrOTOF-Q III 8228888.20448
Comment	SB		

Acquisition Parameter

Source Type	ESI	Ion Polarity	Positive	Set Nebulizer	0.4 Bar
Focus	Not active	Set Capillary	4500 V	Set Dry Heater	180 °C
Scan Begin	50 m/z	Set End Plate Offset	-500 V	Set Dry Gas	4.0 l/min
Scan End	1000 m/z	Set Collision Cell RF	140.0 Vpp	Set Divert Valve	Waste

#	RT [min]	Area	Int. Type	I	S/N	Chromatogram	Max. m/z	FWHM [min]
n.a.	0.0	n.a.	Single spectrum	n.a.	n.a.	n.a.	226.9513	n.a.
n.a.	5.8	n.a.	Single spectrum	n.a.	n.a.	n.a.	286.1590	n.a.

+MS, 5.8min #345

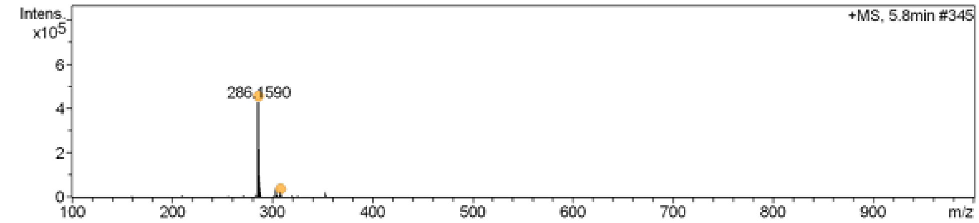

Meas. m/z	#	Ion Formula	m/z	err [ppm]	mSigma	# Sigma	Score	rdb	e⁻ Conf	N-Rule
286.1590	1	C21H20N	286.1590	-0.0	0.8	1	100.00	12.5	even	ok
308.1405	1	C21H19NNa	308.1410	-1.4	14.5	1	100.00	12.5	even	ok

Figure S8. 2,3,3-Trimethyl-5-(naphthalen-2-yl)-3*H*-indole (**3**). HRMS (ESI-TOF).

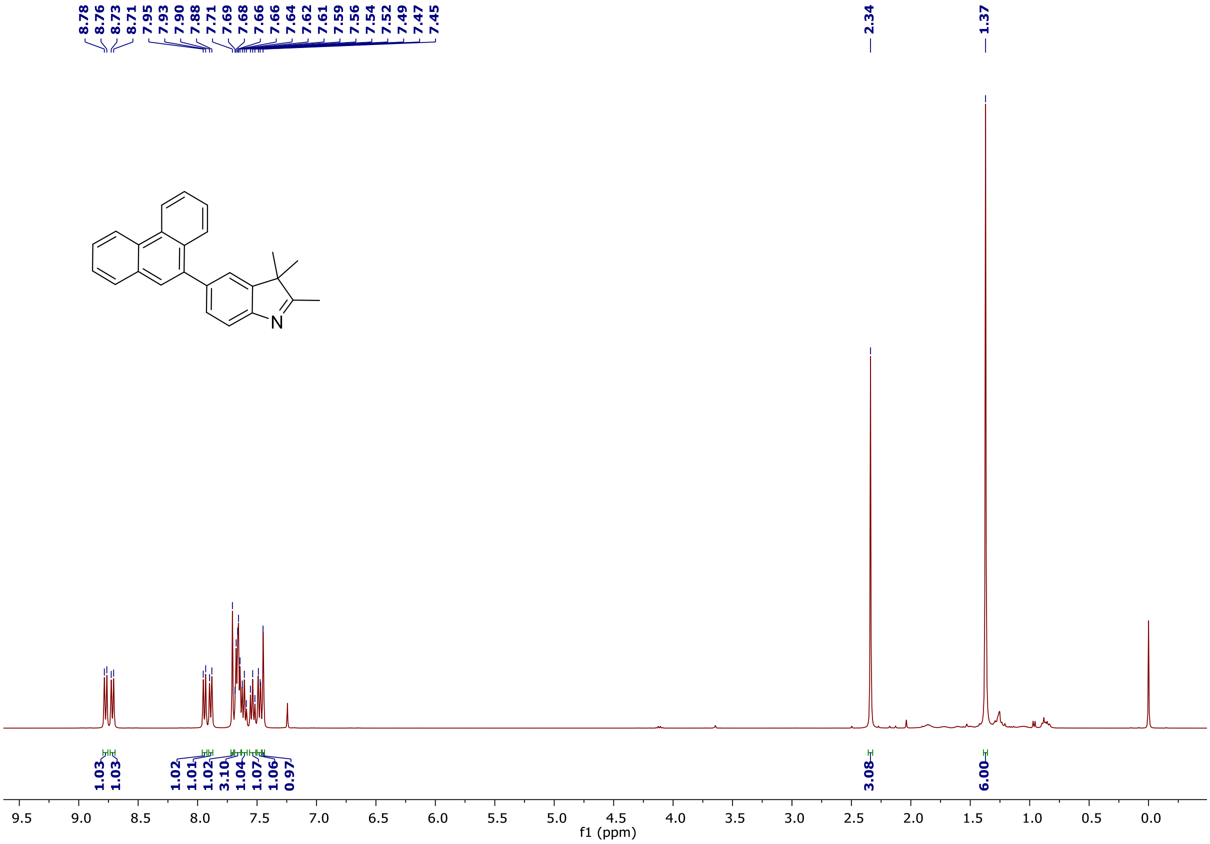

Figure S9. 2,3,3-Trimethyl-5-(phenanthren-9-yl)-3*H*-indole (**4**). ^{1}H NMR spectrum (400 MHz, $CDCl_3$).

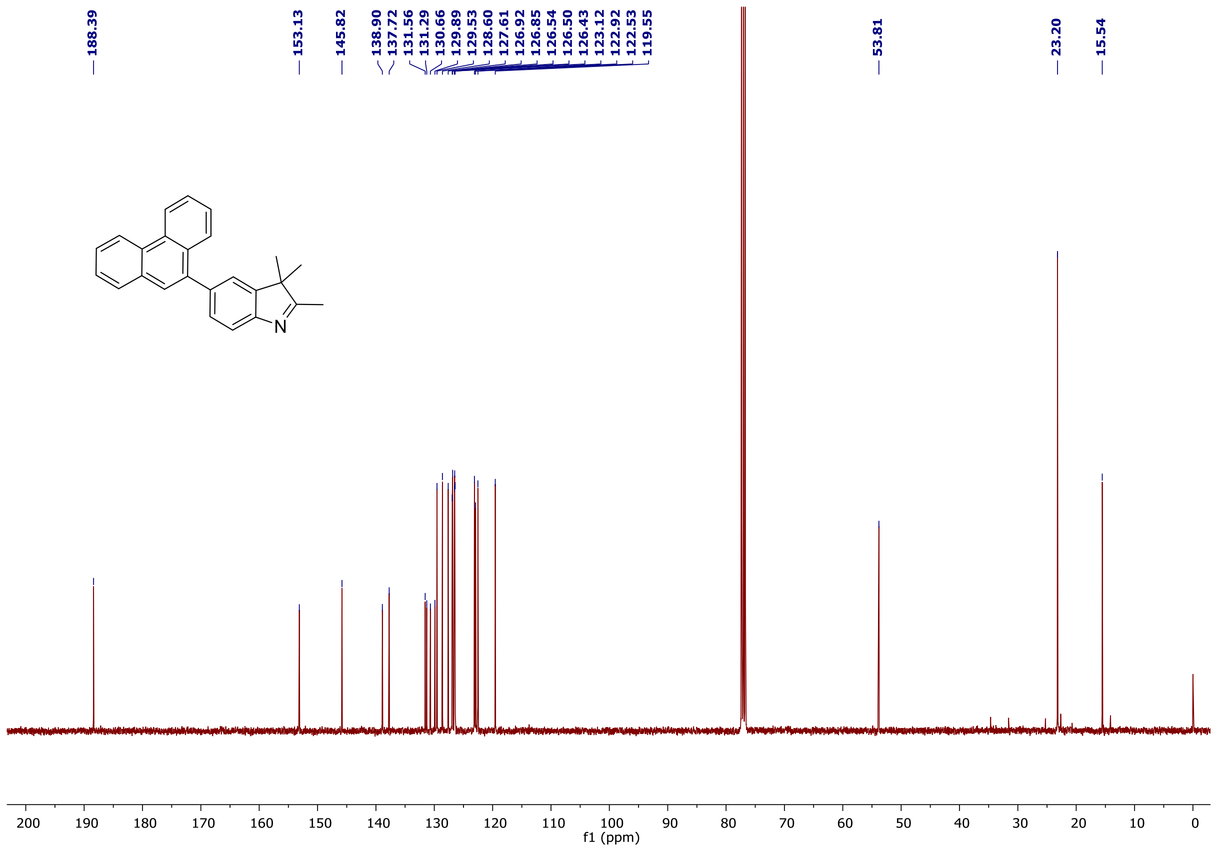

Figure S10. 2,3,3-Trimethyl-5-(phenanthren-9-yl)-3*H*-indole (**4**). ^{13}C NMR spectrum (101 MHz, $CDCl_3$).

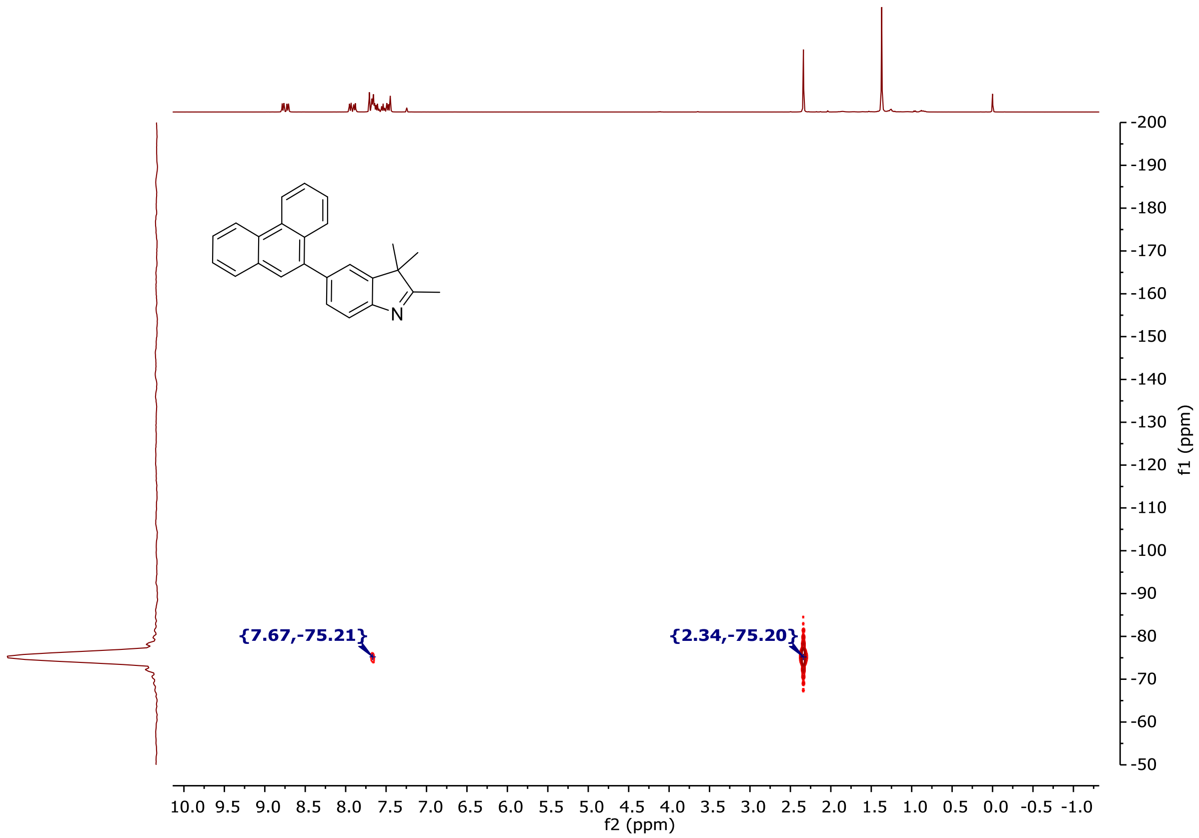

Figure S11. 2,3,3-Trimethyl-5-(phenanthren-9-yl)-3*H*-indole (**4**). ^{1}H-^{15}N HMBC NMR spectrum (71 MHz, $CDCl_3$).

Compound Spectrum SmartFormula Report

Analysis Info

Analysis Name	D:\Data\MRB-32.d	Acquisition Date	3/12/2025 3:52:55 PM
Method	DirectInfusion_TuneLow_pos.m	Operator	hplc
Sample Name	MRB-32	Instrument	micrOTOF-Q III 8228888.20448
Comment	SB		

Acquisition Parameter

Source Type	ESI	Ion Polarity	Positive	Set Nebulizer	0.4 Bar
Focus	Not active	Set Capillary	4500 V	Set Dry Heater	180 °C
Scan Begin	50 m/z	Set End Plate Offset	-500 V	Set Dry Gas	4.0 l/min
Scan End	1000 m/z	Set Collision Cell RF	140.0 Vpp	Set Divert Valve	Waste

#	RT [min]	Area	Int. Type	I	S/N	Chromatogram	Max. m/z	FWHM [min]
n.a.	0.4	n.a.	Single spectrum	n.a.	n.a.	n.a.	226.9525	n.a.
n.a.	3.0	n.a.	Single spectrum	n.a.	n.a.	n.a.	336.1747	n.a.

+MS, 3.0min #181

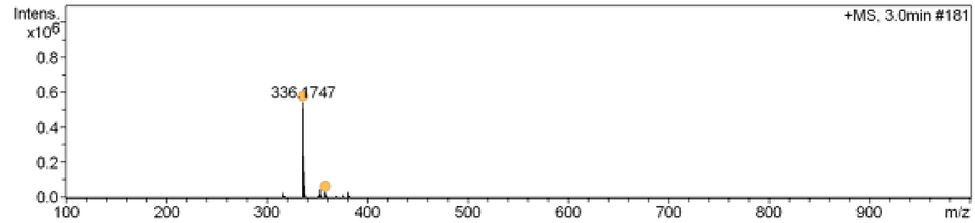

Meas. m/z	#	Ion Formula	m/z	err [ppm]	mSigma	# Sigma	Score	rdb	e⁻ Conf	N-Rule
336.1747	1	C25H22N	336.1747	-0.0	11.3	1	100.00	15.5	even	ok
358.1558	1	C25H21NNa	358.1566	2.3	74.2	1	100.00	15.5	even	ok

Figure S12. 2,3,3-Trimethyl-5-(phenanthren-9-yl)-3*H*-indole (**4**). HRMS (ESI-TOF).

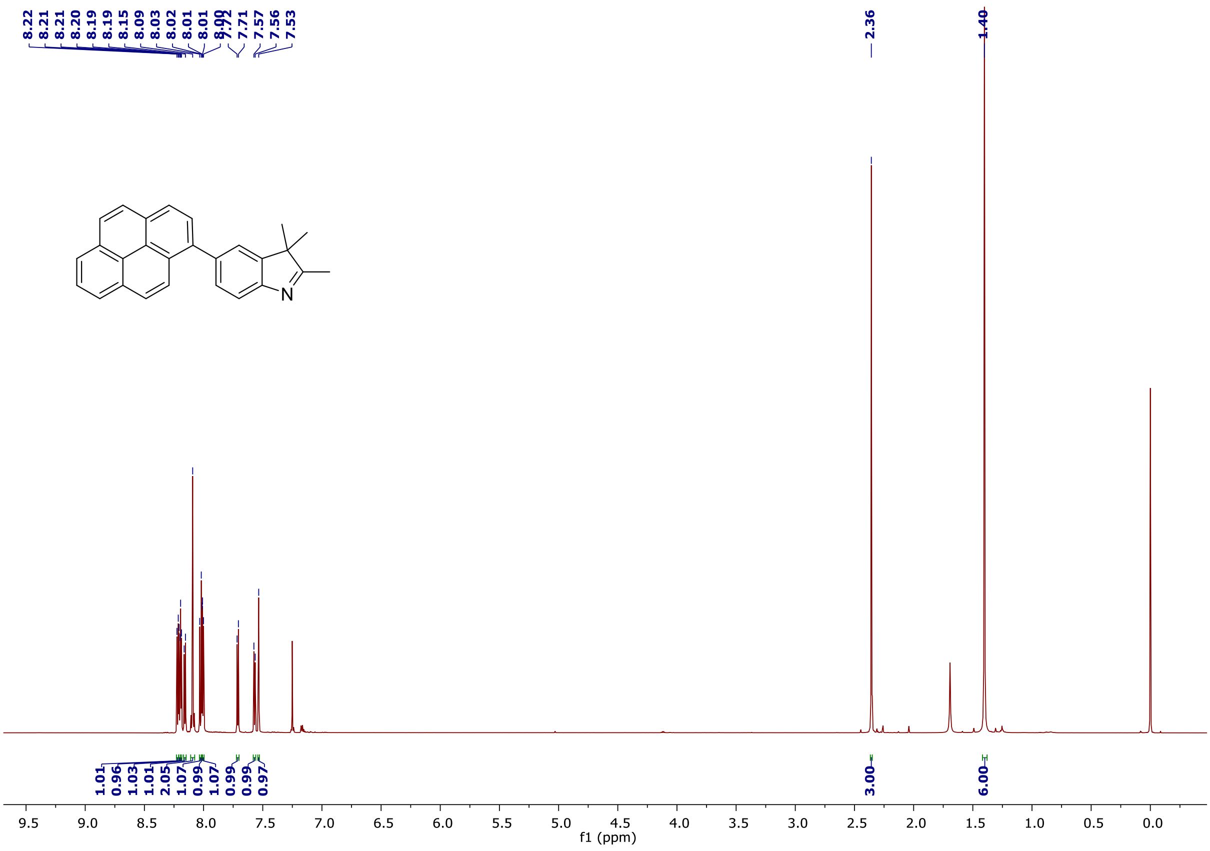

Figure S13. 2,3,3-Trimethyl-5-(pyren-1-yl)-3*H*-indole (**5**). ^{1}H NMR spectrum (700 MHz, $CDCl_3$).

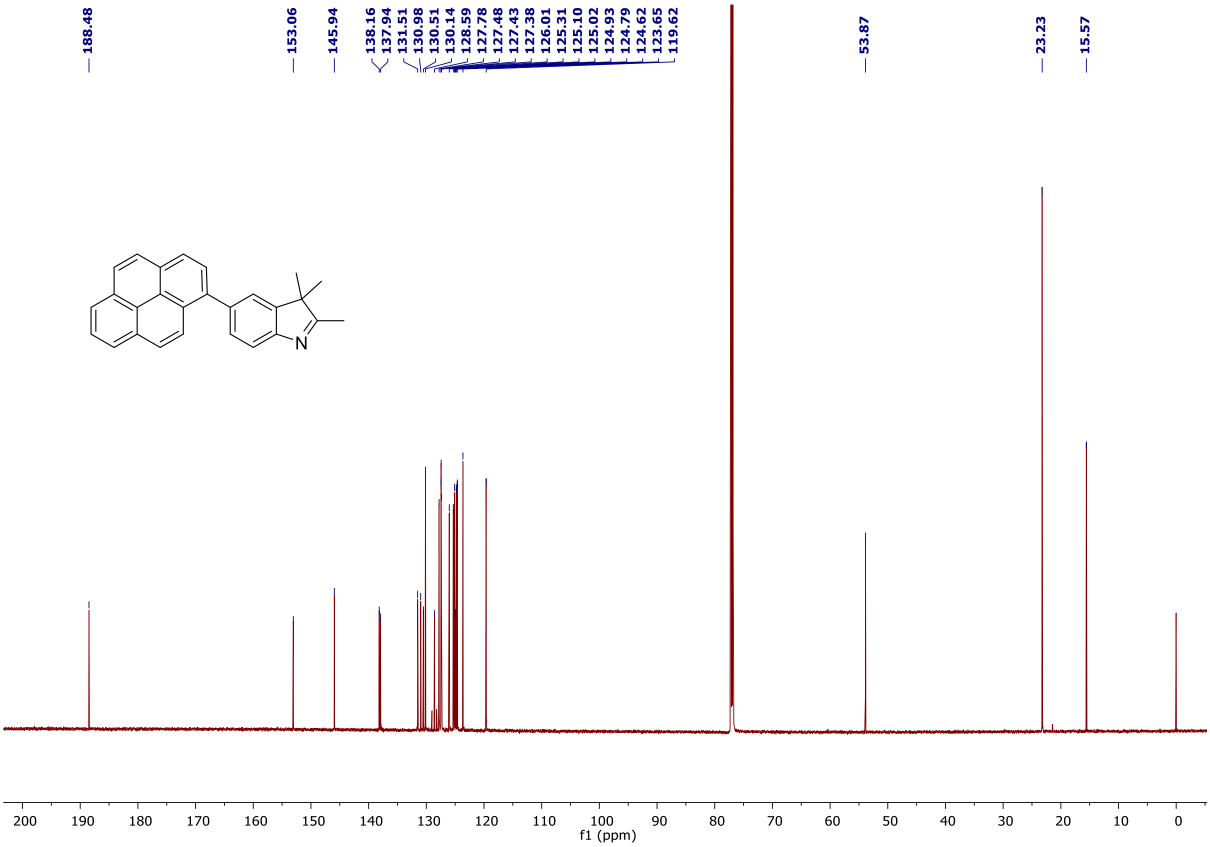

Figure S14. 2,3,3-Trimethyl-5-(pyren-1-yl)-3*H*-indole (**5**). ^{13}C NMR spectrum (176 MHz, $CDCl_3$).

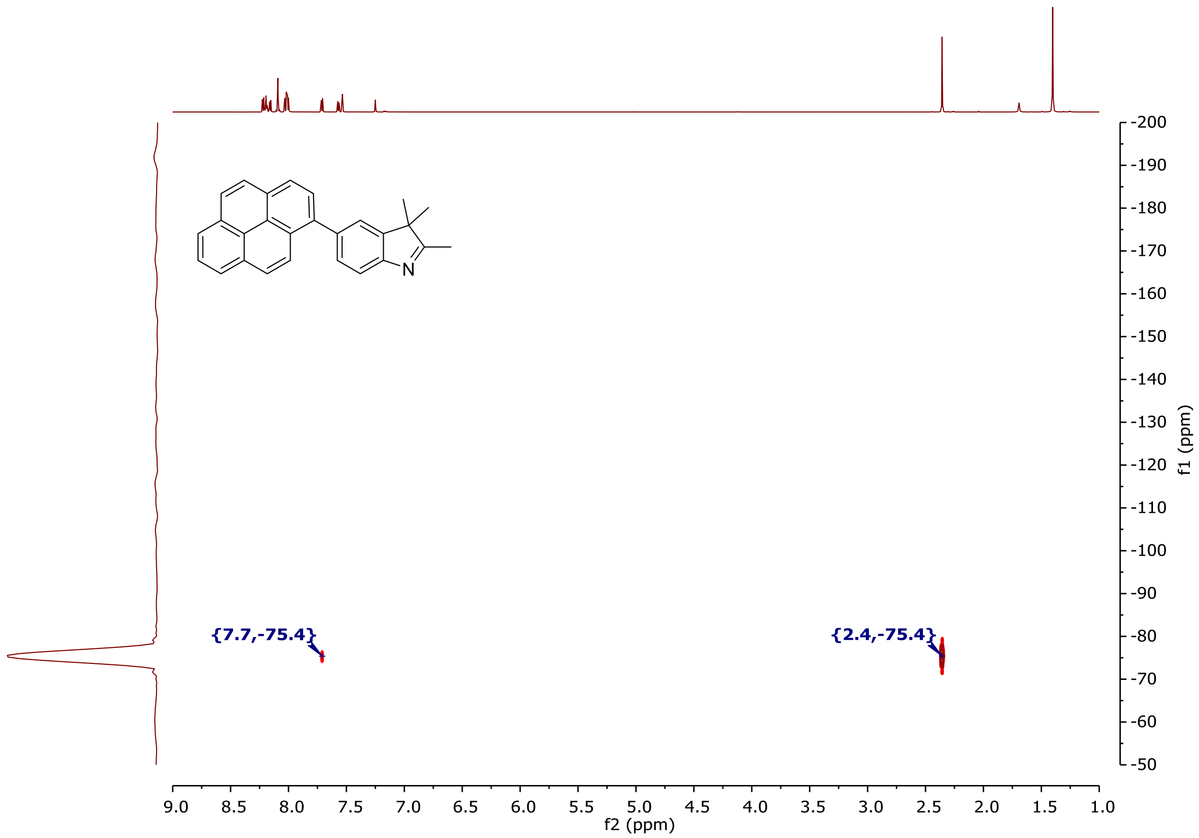

Figure S15. 2,3,3-Trimethyl-5-(pyren-1-yl)-3*H*-indole (**5**). ^{1}H-^{15}N HMBC NMR spectrum (71 MHz, $CDCl_3$).

Compound Spectrum SmartFormula Report

Analysis Info

Analysis Name	D:\Data\MRB-31.d	Acquisition Date	3/12/2025 3:37:03 PM
Method	DirectInfusion_TuneLow_pos.m	Operator	hplc
Sample Name	MRB-31	Instrument	micrOTOF-Q III 8228888.20448
Comment	SB		

Acquisition Parameter

Source Type	ESI	Ion Polarity	Positive	Set Nebulizer	0.4 Bar
Focus	Not active	Set Capillary	4500 V	Set Dry Heater	180 °C
Scan Begin	50 m/z	Set End Plate Offset	-500 V	Set Dry Gas	4.0 l/min
Scan End	1000 m/z	Set Collision Cell RF	140.0 Vpp	Set Divert Valve	Waste

#	RT [min]	Area	Int. Type	I	S/N	Chromatogram	Max. m/z	FWHM [min]
n.a.	0.1	n.a.	Single spectrum	n.a.	n.a.	n.a.	226.9525	n.a.
n.a.	3.6	n.a.	Single spectrum	n.a.	n.a.	n.a.	360.1747	n.a.

+MS, 3.6min #218

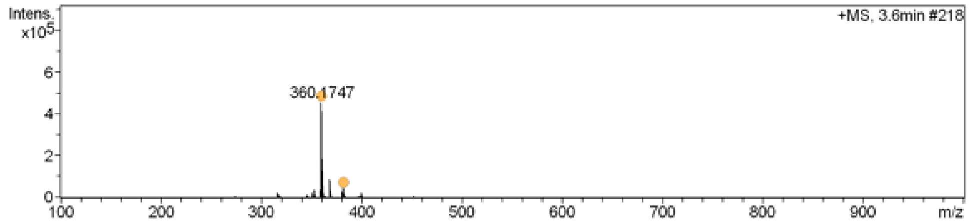

Meas. m/z	#	Ion Formula	m/z	err [ppm]	mSigma	# Sigma	Score	rdb	e⁻ Conf	N-Rule
360.1747	1	C27H22N	360.1747	0.2	5.3	1	100.00	17.5	even	ok
382.1555	1	C27H21NNa	382.1566	3.0	56.6	1	100.00	17.5	even	ok

Figure S16. 2,3,3-Trimethyl-5-(pyren-1-yl)-3*H*-indole (**5**). HRMS (ESI-TOF).

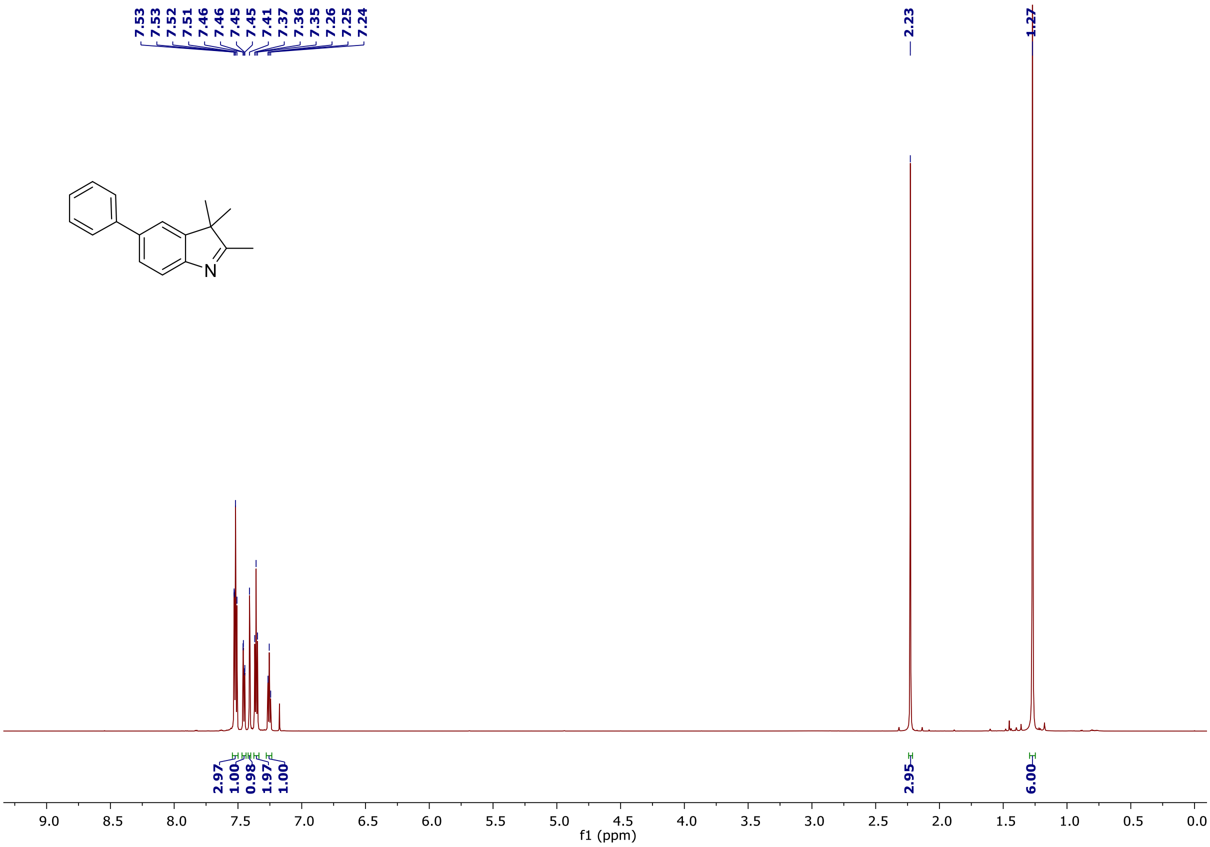

Figure S17. 2,3,3-Trimethyl-5-phenyl-3*H*-indole (**6**). ^{1}H NMR spectrum (700 MHz, $CDCl_3$).

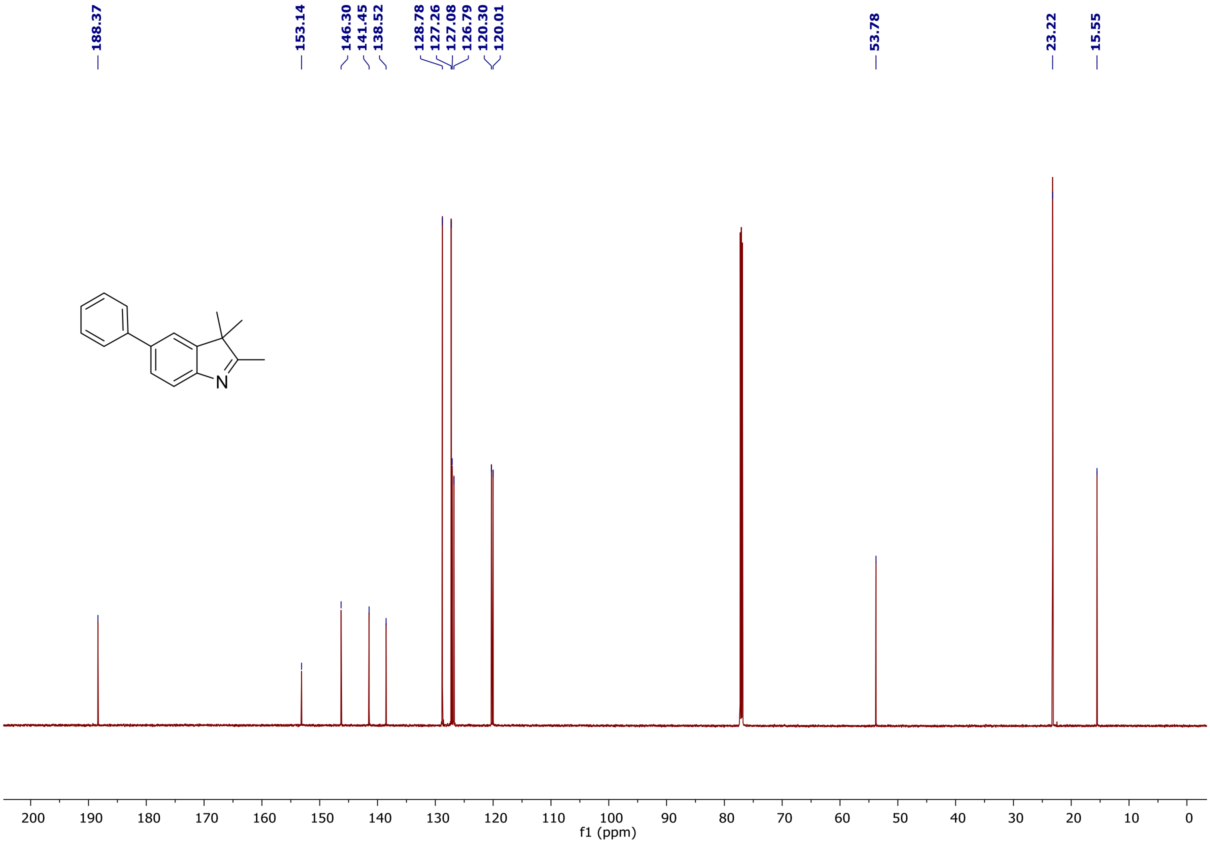

Figure S18. 2,3,3-Trimethyl-5-phenyl-3*H*-indole (**6**). ^{13}C NMR spectrum (176 MHz, $CDCl_3$).

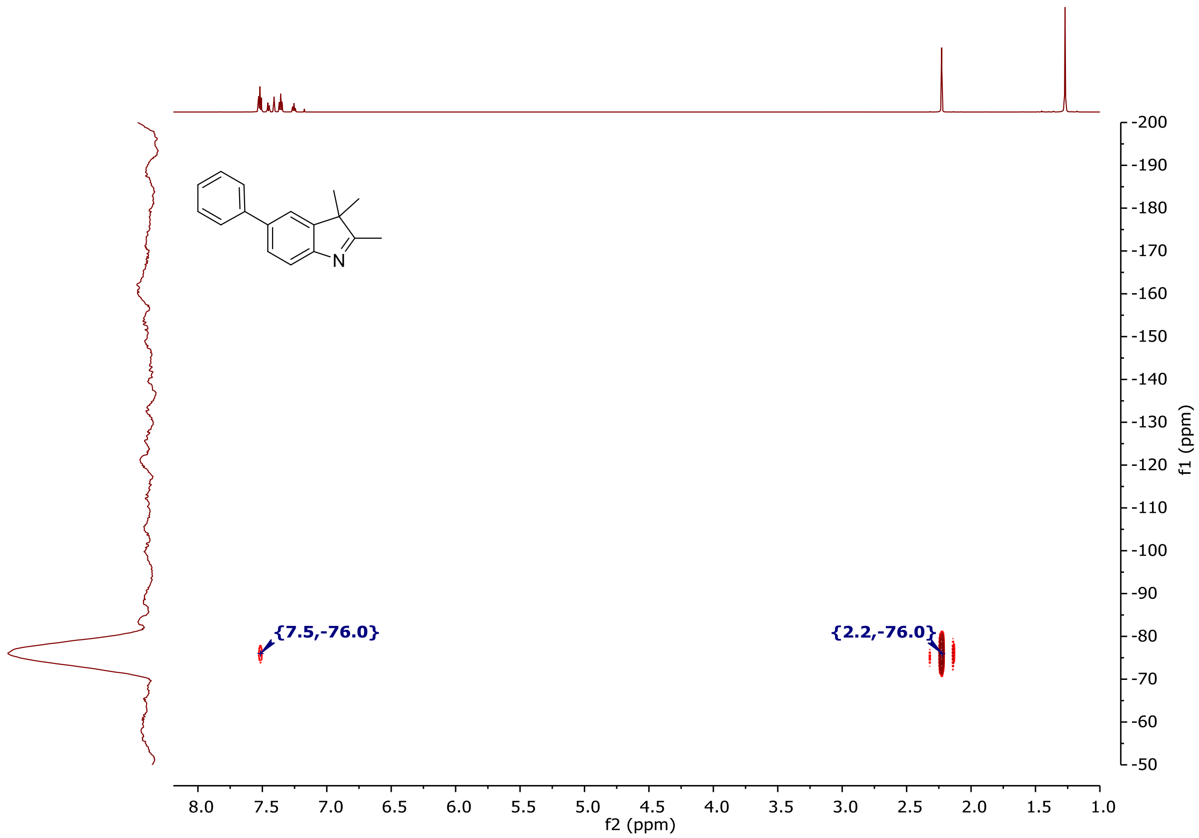

Figure S19. 2,3,3-Trimethyl-5-phenyl-3*H*-indole (**6**). ^{1}H-^{15}N HMBC NMR spectrum (71 MHz, $CDCl_3$).

Compound Spectrum SmartFormula Report

Analysis Info

Analysis Name	D:\Data\MRB-62.d	Acquisition Date	3/13/2025 10:47:52 AM
Method	DirectInfusion_TuneLow_pos.m	Operator	hplc
Sample Name	MRB-62	Instrument	micrOTOF-Q III 8228888.20448
Comment	SB		

Acquisition Parameter

Source Type	ESI	Ion Polarity	Positive	Set Nebulizer	0.4 Bar
Focus	Not active	Set Capillary	4500 V	Set Dry Heater	180 °C
Scan Begin	50 m/z	Set End Plate Offset	-500 V	Set Dry Gas	4.0 l/min
Scan End	1000 m/z	Set Collision Cell RF	140.0 Vpp	Set Divert Valve	Waste

#	RT [min]	Area	Int. Type	I	S/N	Chromatogram	Max. m/z	FWHM [min]
n.a.	0.2	n.a.	Single spectrum	n.a.	n.a.	n.a.	226.9524	n.a.
n.a.	4.8	n.a.	Single spectrum	n.a.	n.a.	n.a.	236.1434	n.a.

+MS, 4.8min #290

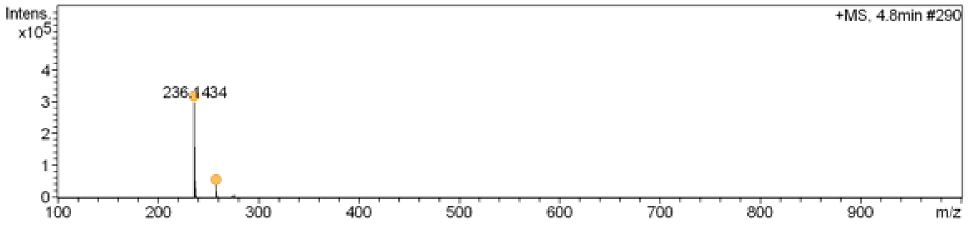

Meas. m/z	#	Ion Formula	m/z	err [ppm]	mSigma	# Sigma	Score	rdb	e⁻ Conf	N-Rule
236.1434	1	C17H18N	236.1434	-0.2	3.9	1	100.00	9.5	even	ok
258.1249	1	C17H17NNa	258.1253	1.7	10.4	1	100.00	9.5	even	ok

Figure S20. 2,3,3-Trimethyl-5-phenyl-3*H*-indole (**6**). HRMS (ESI-TOF).

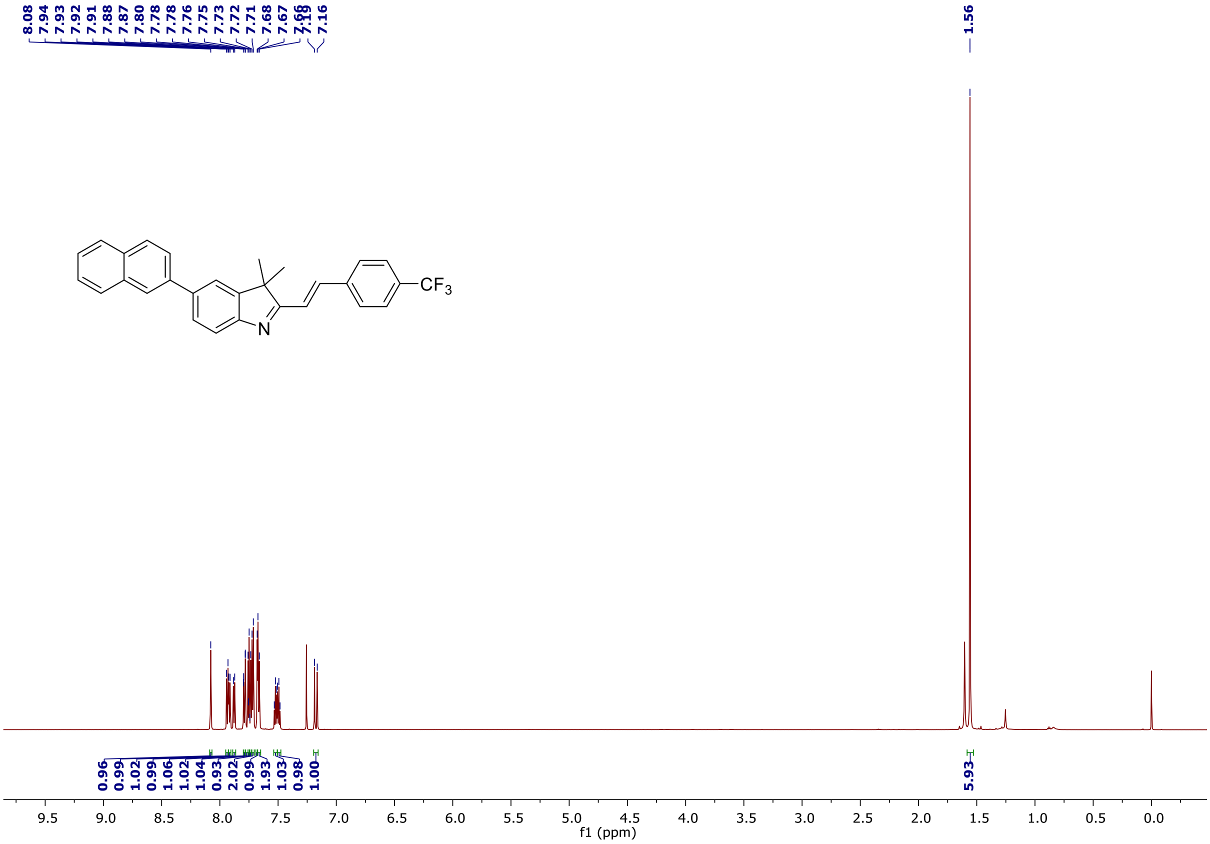

Figure S21. 3,3-Dimethyl-5-(naphthalen-2-yl)-2-{(*E*)-2-[4-(trifluoromethyl)phenyl]ethenyl}-3*H*-indole (**7**). ^{1}H NMR spectrum (700 MHz, $CDCl_3$).

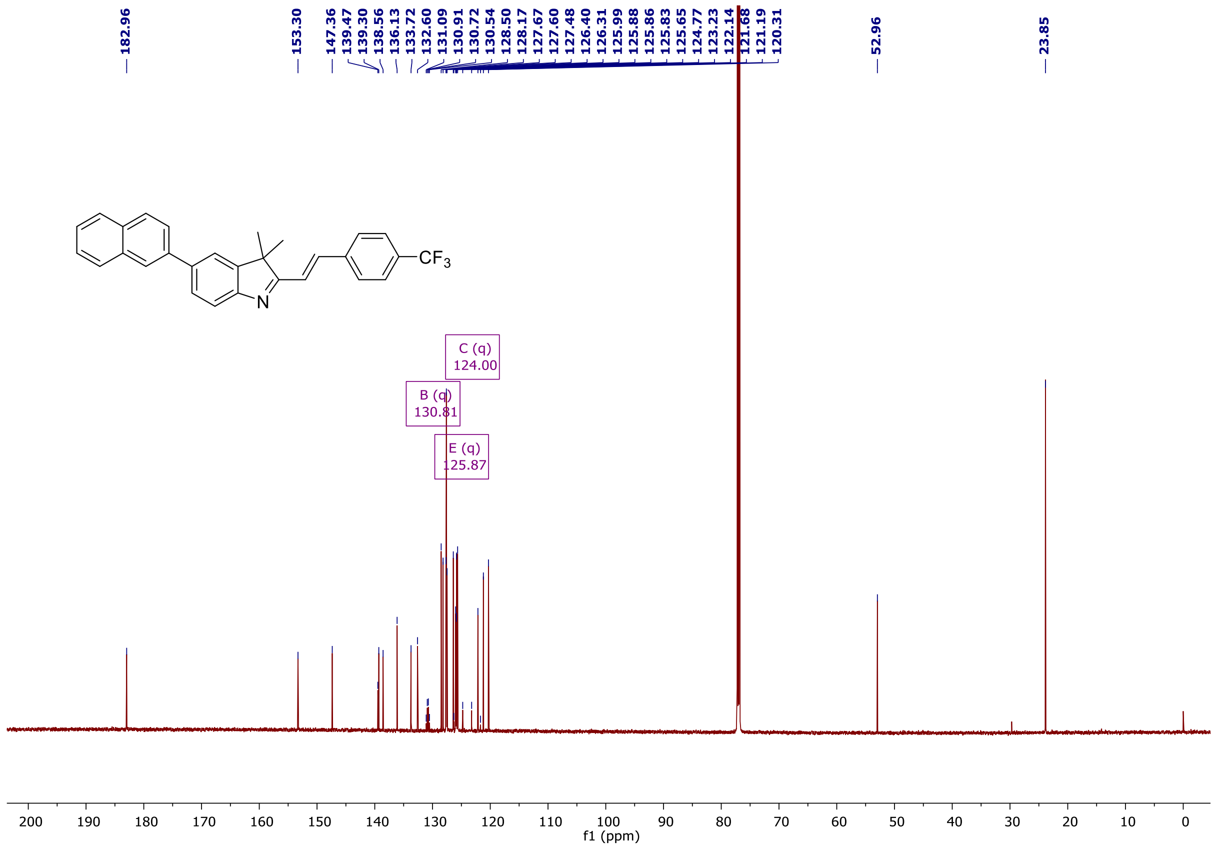

Figure S22. 3,3-Dimethyl-5-(naphthalen-2-yl)-2-{(*E*)-2-[4-(trifluoromethyl)phenyl]ethenyl}-3*H*-indole (**7**). ^{13}C NMR spectrum (176 MHz, $CDCl_3$).

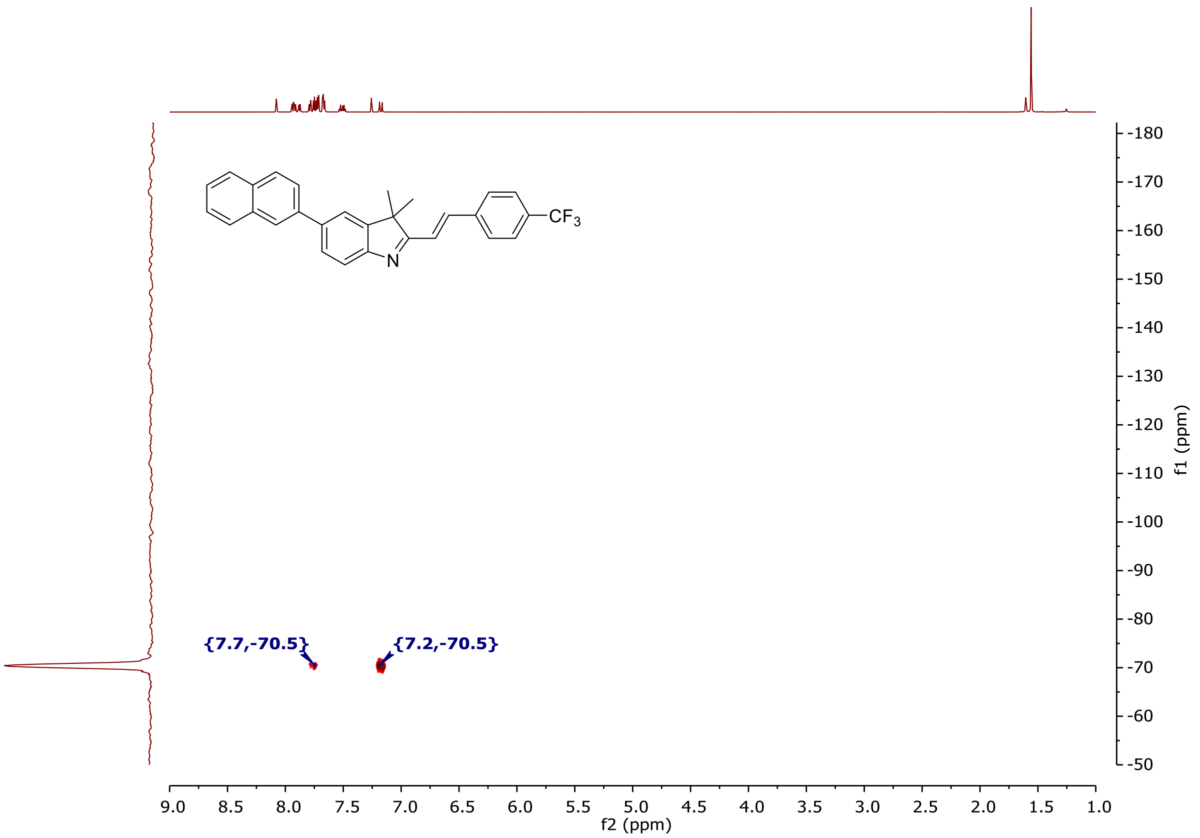

Figure S23. 3,3-Dimethyl-5-(naphthalen-2-yl)-2-{(*E*)-2-[4-(trifluoromethyl)phenyl]ethenyl}-3*H*-indole (**7**). ^{1}H-^{15}N HMBC NMR spectrum (71 MHz, $CDCl_3$).

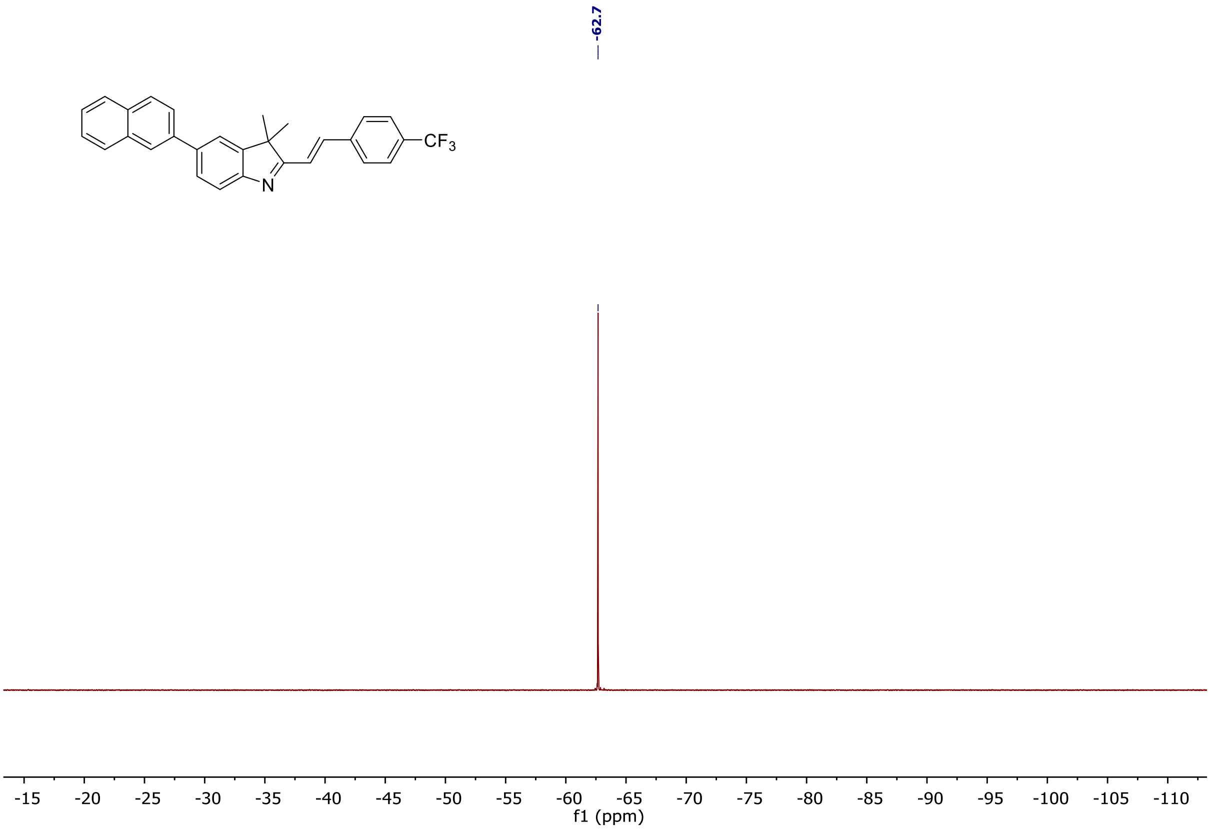

Figure S24. 3,3-Dimethyl-5-(naphthalen-2-yl)-2-{(*E*)-2-[4-(trifluoromethyl)phenyl]ethenyl}-3*H*-indole (**7**). ^{19}F NMR spectrum (376 MHz, $CDCl_3$).

Compound Spectrum SmartFormula Report

Analysis Info

Analysis Name	D:\Data\MRB-6.d	Acquisition Date	8/17/2021 3:06:49 PM
Method	DirectInfusion_TuneLow_pos.m	Operator	hplc
Sample Name	MRB-6	Instrument	micrOTOF-Q III 8228888.20448
Comment	SB		

Acquisition Parameter

Source Type	ESI	Ion Polarity	Positive	Set Nebulizer	0.4 Bar
Focus	Not active	Set Capillary	4500 V	Set Dry Heater	180 °C
Scan Begin	50 m/z	Set End Plate Offset	-500 V	Set Dry Gas	4.0 l/min
Scan End	1000 m/z	Set Collision Cell RF	140.0 Vpp	Set Divert Valve	Waste

#	RT [min]	Area	Int. Type	I	S/N	Chromatogram	Max. m/z	FWHM [min]
n.a.	0.1	n.a.	Single spectrum	n.a.	n.a.	n.a.	226.9521	n.a.
n.a.	3.0	n.a.	Single spectrum	n.a.	n.a.	n.a.	442.1777	n.a.

+MS, 3.0min #177

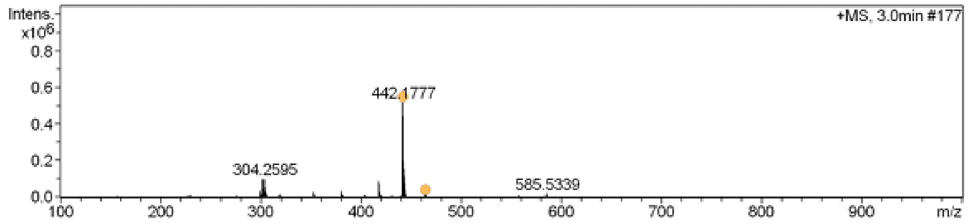

Meas. m/z	#	Ion Formula	m/z	err [ppm]	mSigma	# Sigma	Score	rdb	e⁻ Conf	N-Rule
442.1777	1	C29H23F3N	442.1777	-0.1	0.5	1	100.00	17.5	even	ok
464.1592	1	C29H22F3NNa	464.1597	0.9	25.9	1	100.00	17.5	even	ok

Figure S25. 3,3-Dimethyl-5-(naphthalen-2-yl)-2-{(*E*)-2-[4-(trifluoromethyl)phenyl]ethenyl}-3*H*-indole (**7**). HRMS (ESI-TOF).

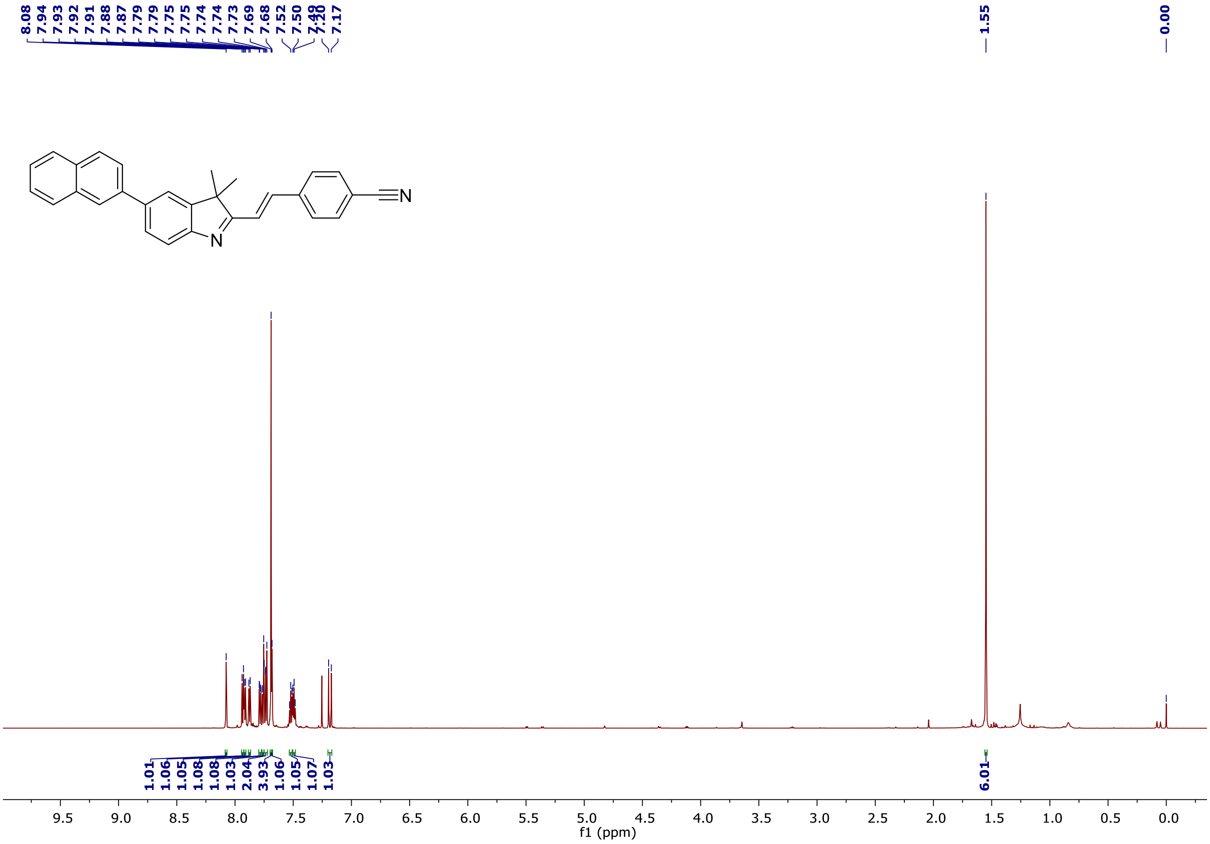

Figure S26. 4-{(*E*)-2-[3,3-Dimethyl-5-(naphthalen-2-yl)-3*H*-indol-2-yl]ethenyl}benzonitrile (**8**). ^{1}H NMR spectrum (700 MHz, $CDCl_3$).

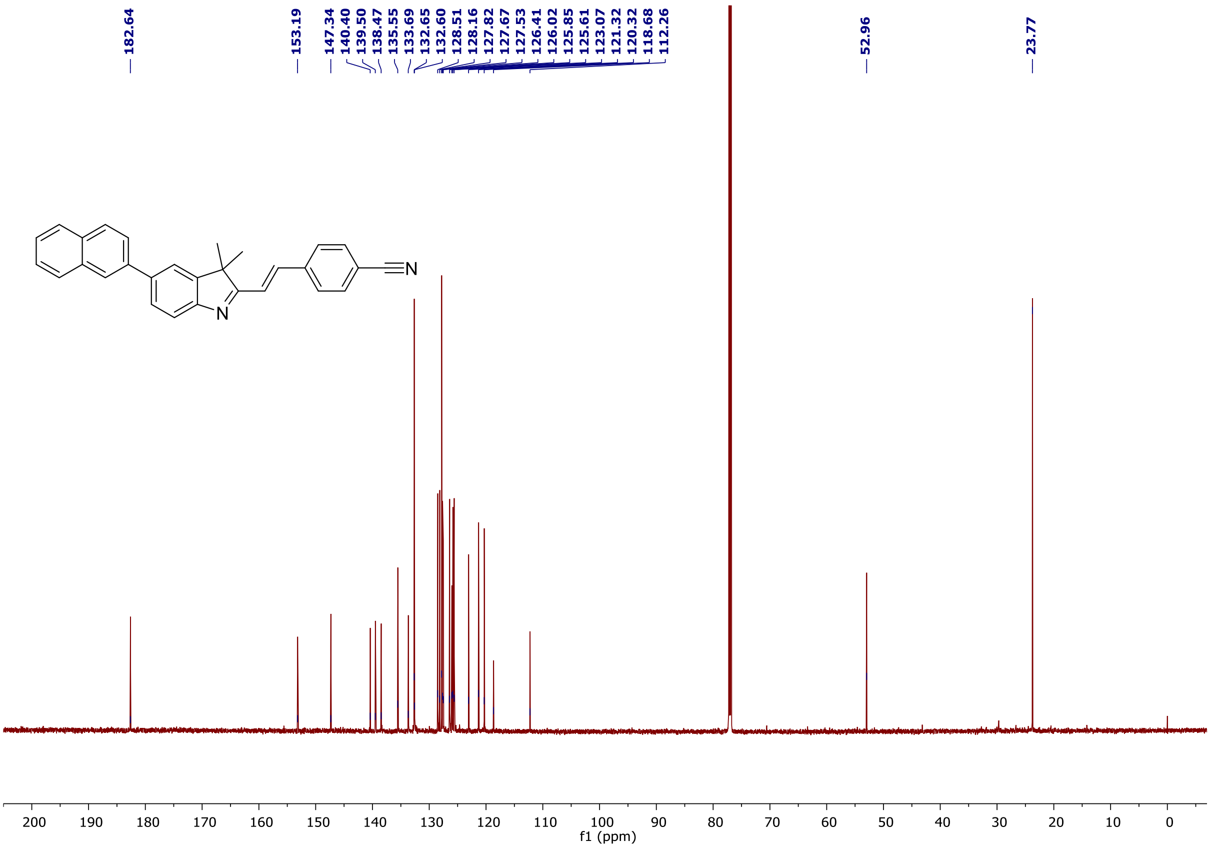

Figure S27. 4-{(*E*)-2-[3,3-Dimethyl-5-(naphthalen-2-yl)-3*H*-indol-2-yl]ethenyl}benzonitrile (**8**). ^{13}C NMR spectrum (176 MHz, $CDCl_3$).

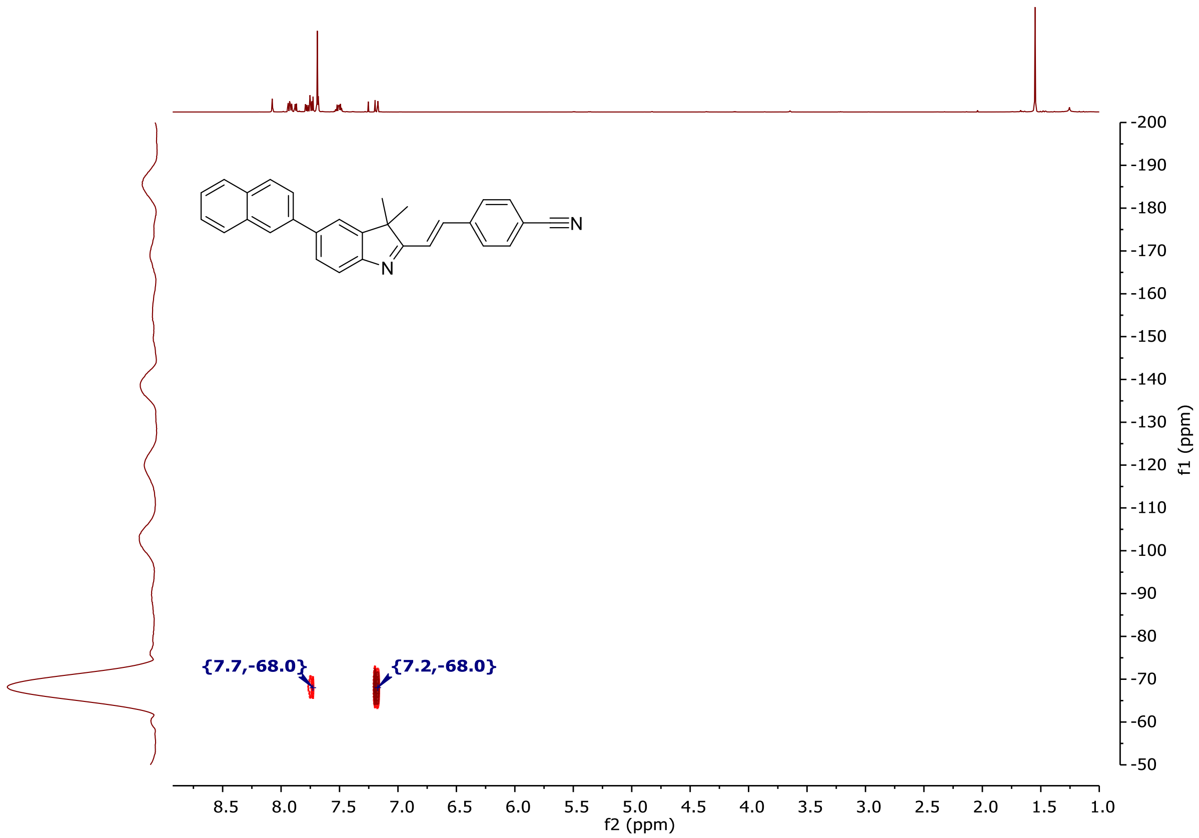

Figure S28. 4-{(*E*)-2-[3,3-Dimethyl-5-(naphthalen-2-yl)-3*H*-indol-2-yl]ethenyl}benzonitrile (**8**). ^{1}H-^{15}N HMBC NMR spectrum (71 MHz, $CDCl_3$).

Compound Spectrum SmartFormula Report

Analysis Info

Analysis Name	D:\Data\MRB-23.d	Acquisition Date	3/12/2025 3:17:59 PM
Method	DirectInfusion_TuneLow_pos.m	Operator	hplc
Sample Name	MRB-23	Instrument	micrOTOF-Q III 8228888.20448
Comment	SB		

Acquisition Parameter

Source Type	ESI	Ion Polarity	Positive	Set Nebulizer	0.4 Bar
Focus	Not active	Set Capillary	4500 V	Set Dry Heater	180 °C
Scan Begin	50 m/z	Set End Plate Offset	-500 V	Set Dry Gas	4.0 l/min
Scan End	1000 m/z	Set Collision Cell RF	140.0 Vpp	Set Divert Valve	Waste

#	RT [min]	Area	Int. Type	I	S/N	Chromatogram	Max. m/z	FWHM [min]
n.a.	0.3	n.a.	Single spectrum	n.a.	n.a.	n.a.	226.9526	n.a.
n.a.	6.1	n.a.	Single spectrum	n.a.	n.a.	n.a.	399.1856	n.a.

+MS, 6.1min #366

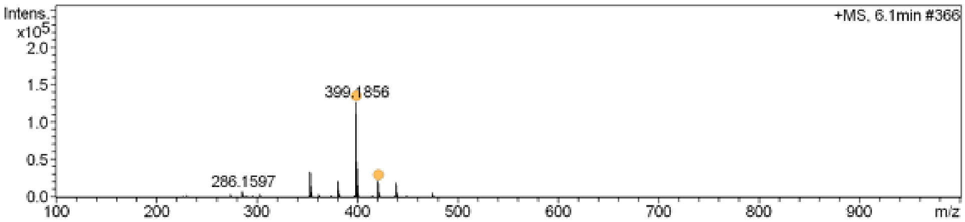

Meas. m/z	#	Ion Formula	m/z	err [ppm]	mSigma	# Sigma	Score	rdb	e⁻ Conf	N-Rule
399.1856	1	C29H23N2	399.1856	-0.2	7.9	1	100.00	19.5	even	ok
421.1688	1	C29H22N2Na	421.1675	-3.0	5.6	1	100.00	19.5	even	ok

Figure S29. 4-{(*E*)-2-[3,3-Dimethyl-5-(naphthalen-2-yl)-3*H*-indol-2-yl]ethenyl}benzonitrile (**8**). HRMS (ESI-TOF).

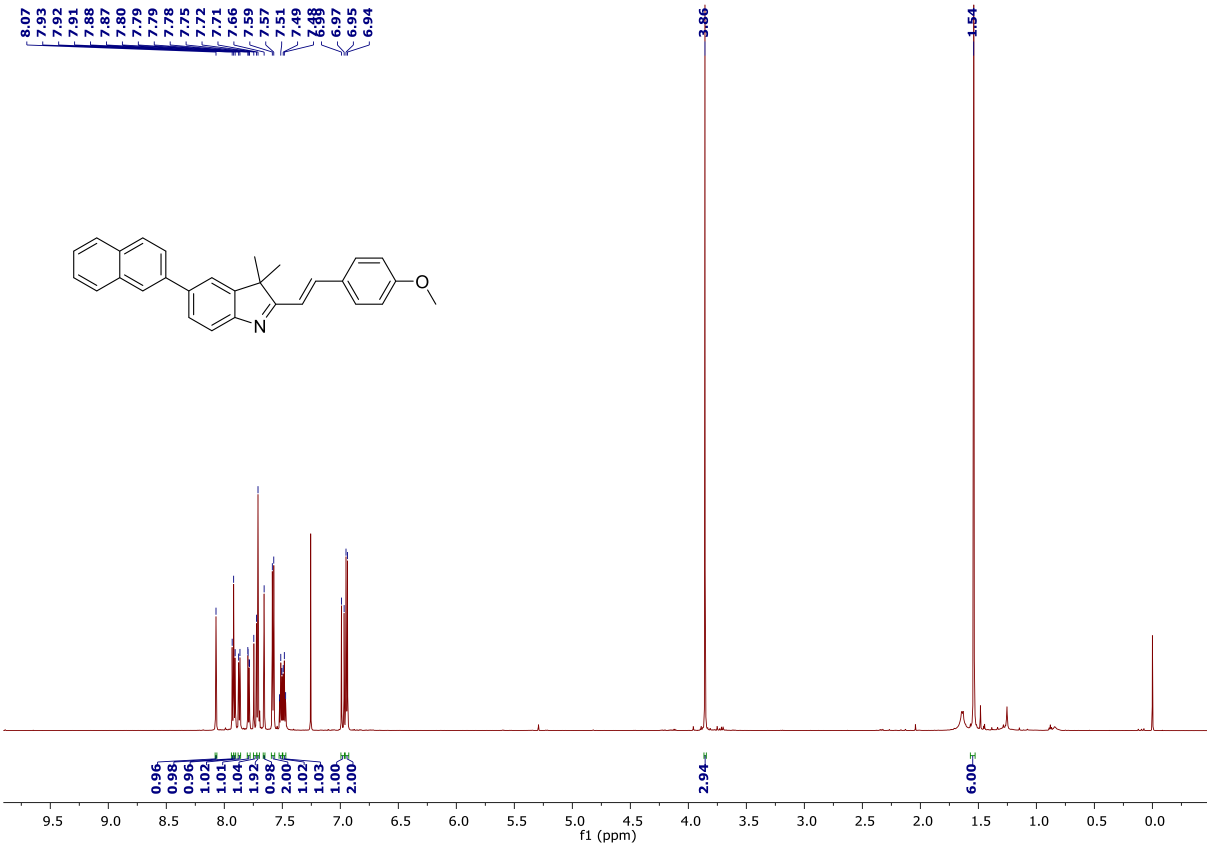

Figure S30. 2-[(*E*)-2-(4-Methoxyphenyl)ethenyl]-3,3-dimethyl-5-(naphthalen-2-yl)-3*H*-indole (**9**). ^{1}H NMR spectrum (700 MHz, $CDCl_3$).

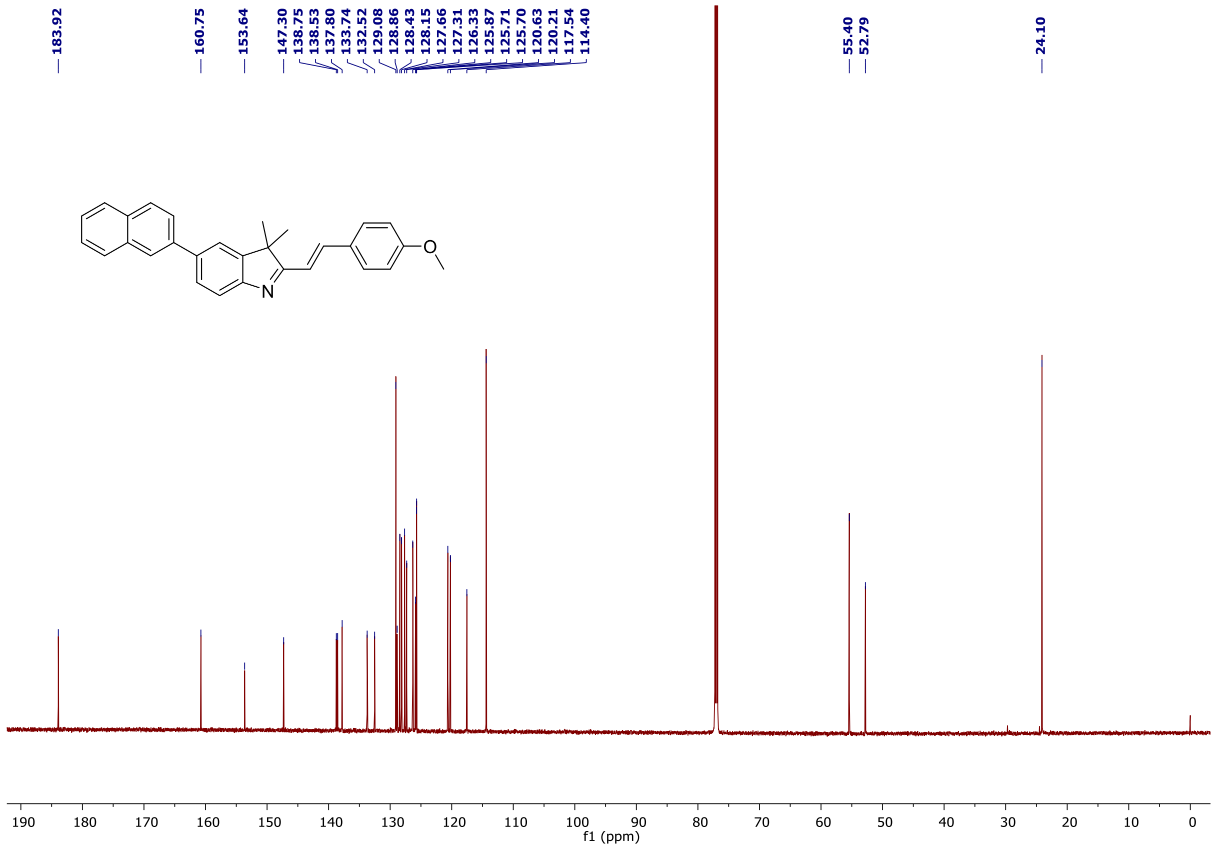

Figure S31. 2-[(*E*)-2-(4-Methoxyphenyl)ethenyl]-3,3-dimethyl-5-(naphthalen-2-yl)-3*H*-indole (**9**). ^{13}C NMR spectrum (176 MHz, $CDCl_3$).

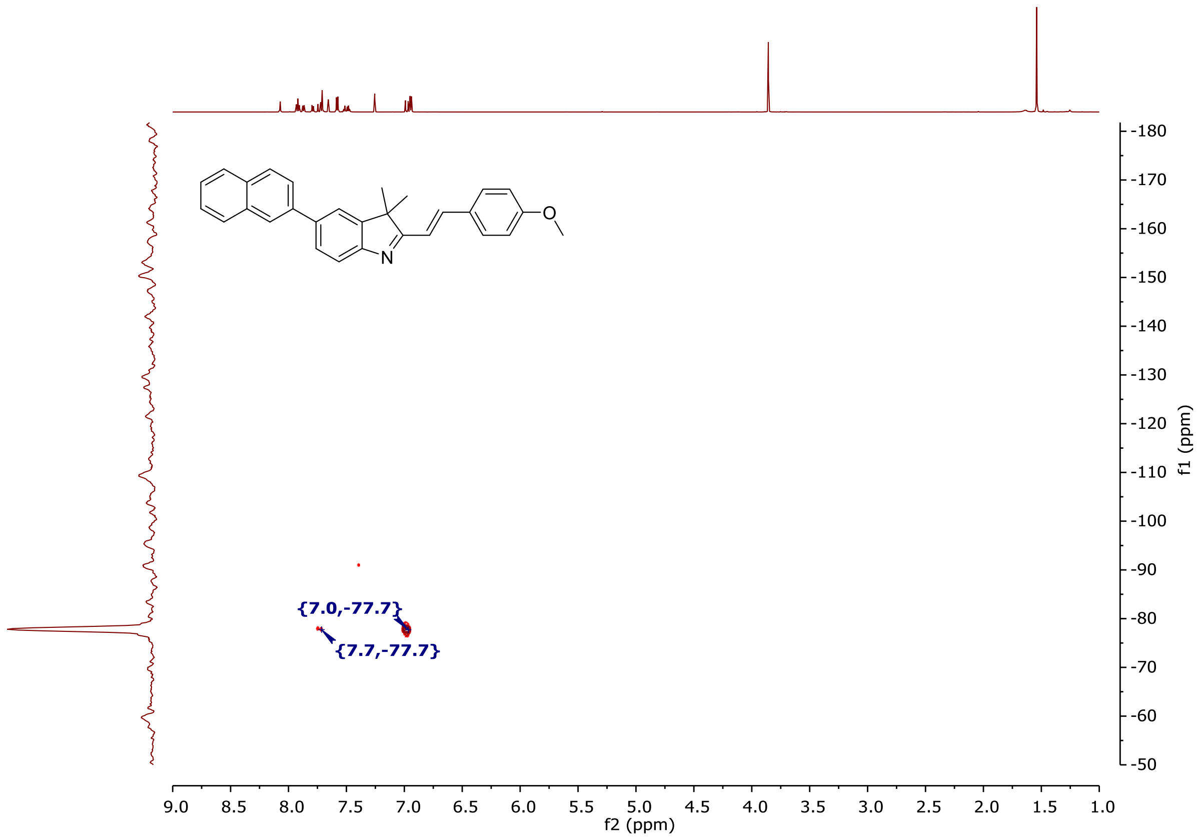

Figure S32. 2-[(*E*)-2-(4-Methoxyphenyl)ethenyl]-3,3-dimethyl-5-(naphthalen-2-yl)-3*H*-indole (**9**). ^{1}H-^{15}N HMBC NMR spectrum (71 MHz, $CDCl_3$).

Compound Spectrum SmartFormula Report

Analysis Info

Analysis Name	D:\Data\MRB-5.d	Acquisition Date	8/17/2021 2:31:07 PM
Method	DirectInfusion_TuneLow_pos.m	Operator	hplc
Sample Name	MRB-5	Instrument	micrOTOF-Q III 8228888.20448
Comment	SB		

Acquisition Parameter

Source Type	ESI	Ion Polarity	Positive	Set Nebulizer	0.4 Bar
Focus	Not active	Set Capillary	4500 V	Set Dry Heater	180 °C
Scan Begin	50 m/z	Set End Plate Offset	-500 V	Set Dry Gas	4.0 l/min
Scan End	1000 m/z	Set Collision Cell RF	140.0 Vpp	Set Divert Valve	Waste

#	RT [min]	Area	Int. Type	I	S/N	Chromatogram	Max. m/z	FWHM [min]
n.a.	0.2	n.a.	Single spectrum	n.a.	n.a.	n.a.	226.9521	n.a.
n.a.	7.6	n.a.	Single spectrum	n.a.	n.a.	n.a.	404.2009	n.a.

+MS, 7.6min #455

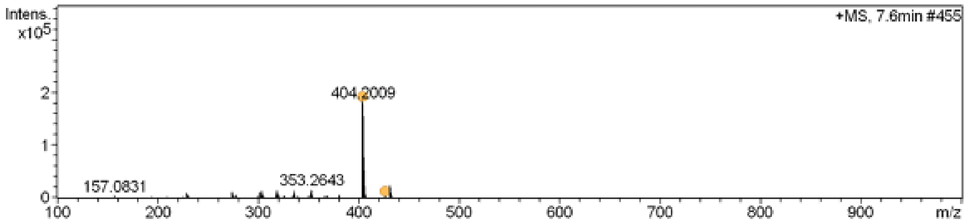

Meas. m/z	#	Ion Formula	m/z	err [ppm]	mSigma	# Sigma	Score	rdb	e⁻ Conf	N-Rule
404.2009	1	C29H26NO	404.2009	-0.1	4.8	1	100.00	17.5	even	ok
426.1841	1	C29H25NNaO	426.1828	3.0	34.2	1	100.00	17.5	even	ok

Figure S33. 2-[(*E*)-2-(4-Methoxyphenyl)ethenyl]-3,3-dimethyl-5-(naphthalen-2-yl)-3*H*-indole (**9**). HRMS (ESI-TOF).

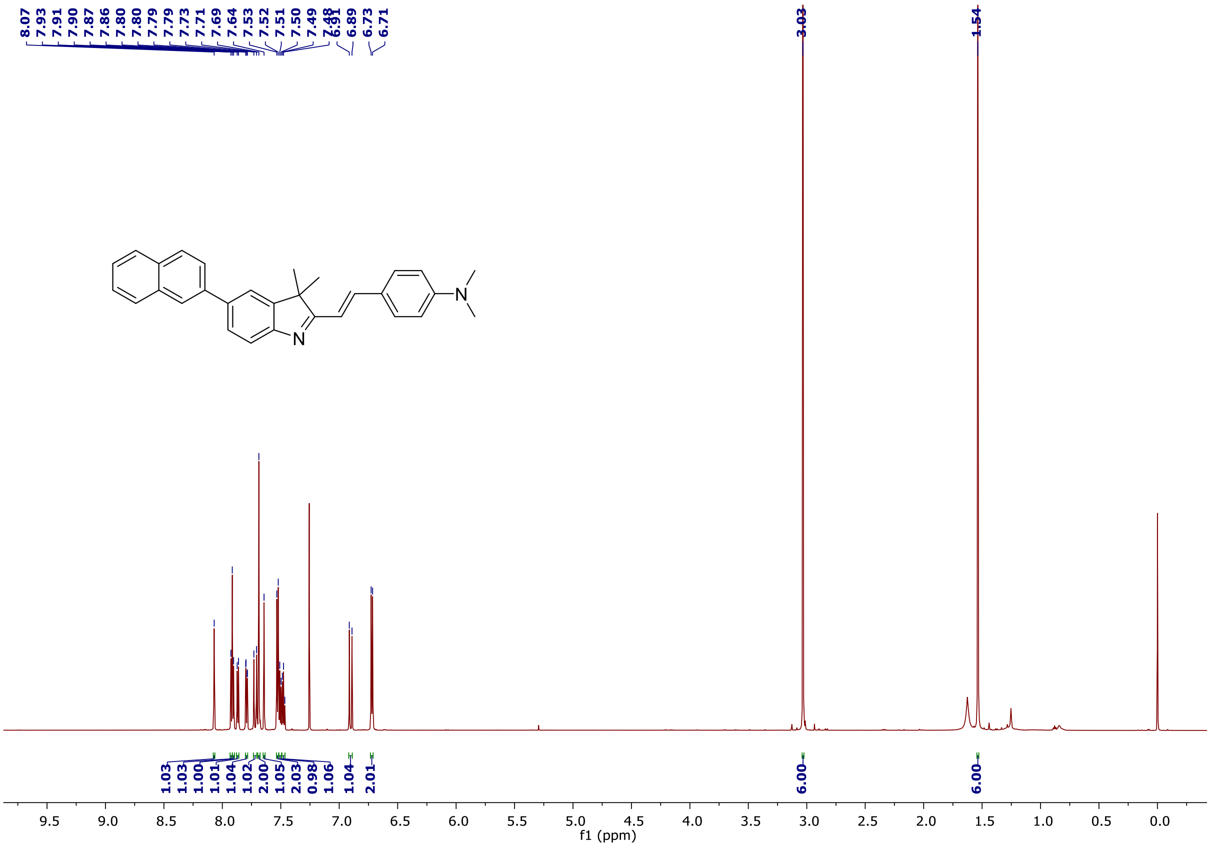

Figure S34. 4-{(*E*)-2-[3,3-Dimethyl-5-(naphthalen-2-yl)-3*H*-indol-2-yl]ethenyl}-*N*,*N*-dimethylaniline (**10**). ^{1}H NMR spectrum (700 MHz, $CDCl_3$).

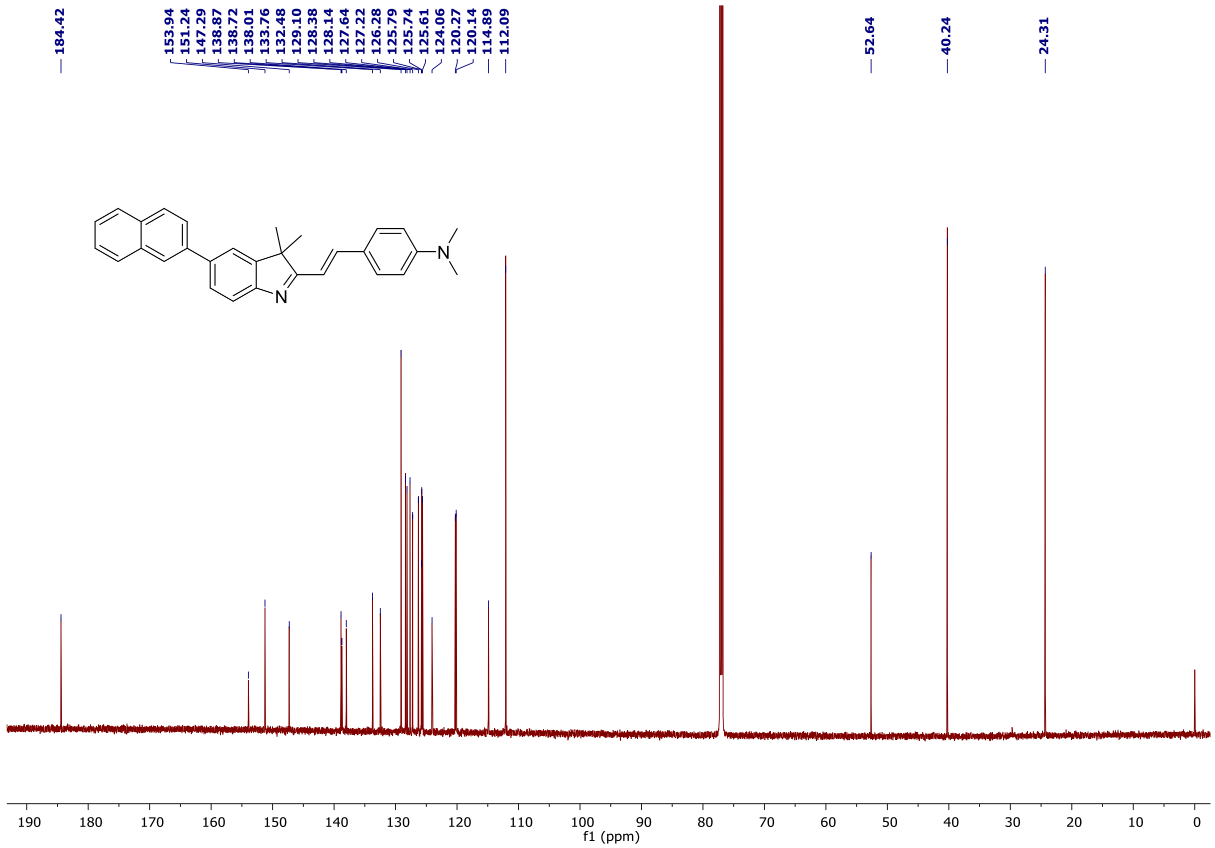

Figure S35. 4-{(*E*)-2-[3,3-Dimethyl-5-(naphthalen-2-yl)-3*H*-indol-2-yl]ethenyl}-*N*,*N*-dimethylaniline (**10**). ^{13}C NMR spectrum (176 MHz, $CDCl_3$).

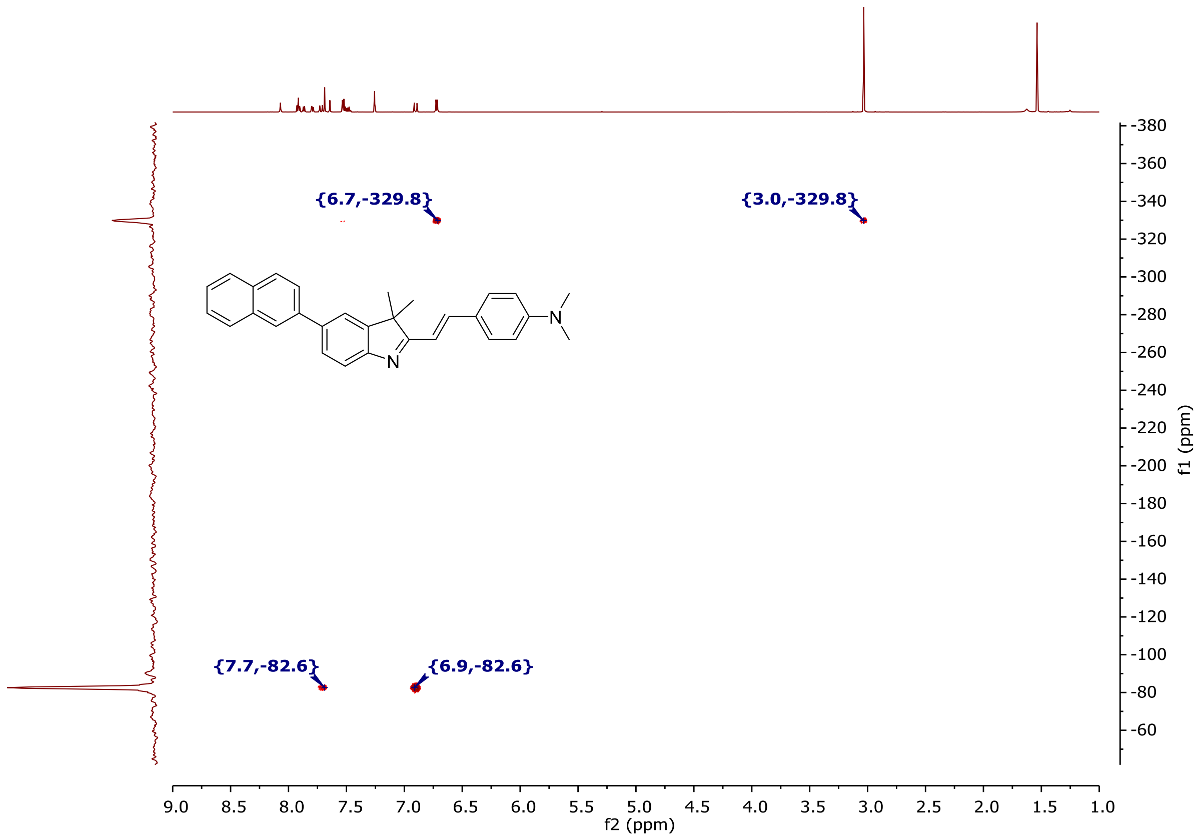

Figure S36. 4-{(*E*)-2-[3,3-Dimethyl-5-(naphthalen-2-yl)-3*H*-indol-2-yl]ethenyl}-*N*,*N*-dimethylaniline (**10**). ^{1}H-^{15}N HMBC NMR spectrum (71 MHz, $CDCl_3$).

Compound Spectrum SmartFormula Report

Analysis Info

Analysis Name	D:\Data\MRB-4.d	Acquisition Date	8/17/2021 12:26:52 PM
Method	DirectInfusion_TuneLow_pos.m	Operator	hplc
Sample Name	MRB-4	Instrument	micrOTOF-Q III 8228888.20448
Comment	SB		

Acquisition Parameter

Source Type	ESI	Ion Polarity	Positive	Set Nebulizer	0.4 Bar
Focus	Not active	Set Capillary	4500 V	Set Dry Heater	180 °C
Scan Begin	50 m/z	Set End Plate Offset	-500 V	Set Dry Gas	4.0 l/min
Scan End	1000 m/z	Set Collision Cell RF	140.0 Vpp	Set Divert Valve	Waste

#	RT [min]	Area	Int. Type	I	S/N	Chromatogram	Max. m/z	FWHM [min]
n.a.	0.0	n.a.	Single spectrum	n.a.	n.a.	n.a.	226.9519	n.a.
n.a.	3.6	n.a.	Single spectrum	n.a.	n.a.	n.a.	417.2325	n.a.

+MS, 3.6min #216

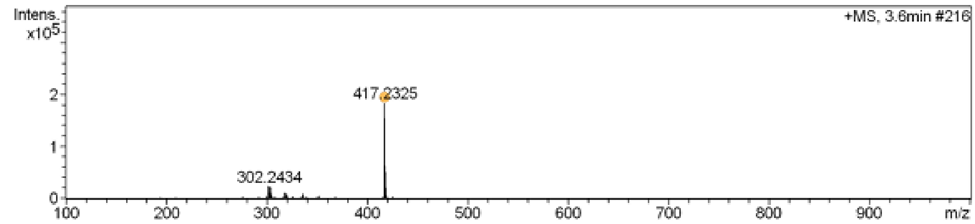

Meas. m/z	#	Ion Formula	m/z	err [ppm]	mSigma	# Sigma	Score	rdb	e⁻ Conf	N-Rule
417.2325	1	C30H29N2	417.2325	-0.1	3.0	1	100.00	17.5	even	ok

Figure S37. 4-{(*E*)-2-[3,3-Dimethyl-5-(naphthalen-2-yl)-3*H*-indol-2-yl]ethenyl}-*N*,*N*-dimethylaniline (**10**). HRMS (ESI-TOF).

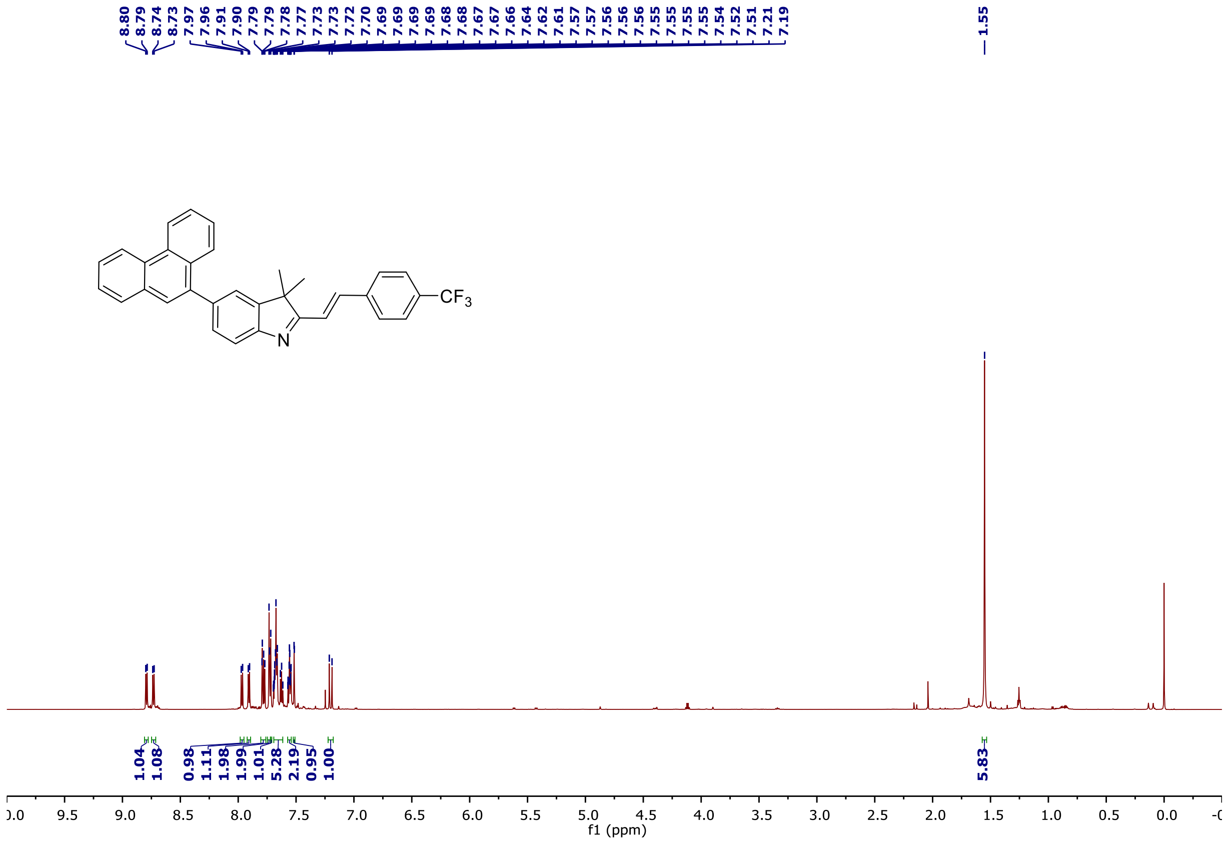

Figure S38. 3,3-Dimethyl-5-(phenanthren-9-yl)-2-{(*E*)-2-[4-(trifluoromethyl)phenyl]ethenyl}-3*H*-indole (**11**). ^{1}H NMR spectrum (700 MHz, $CDCl_3$).

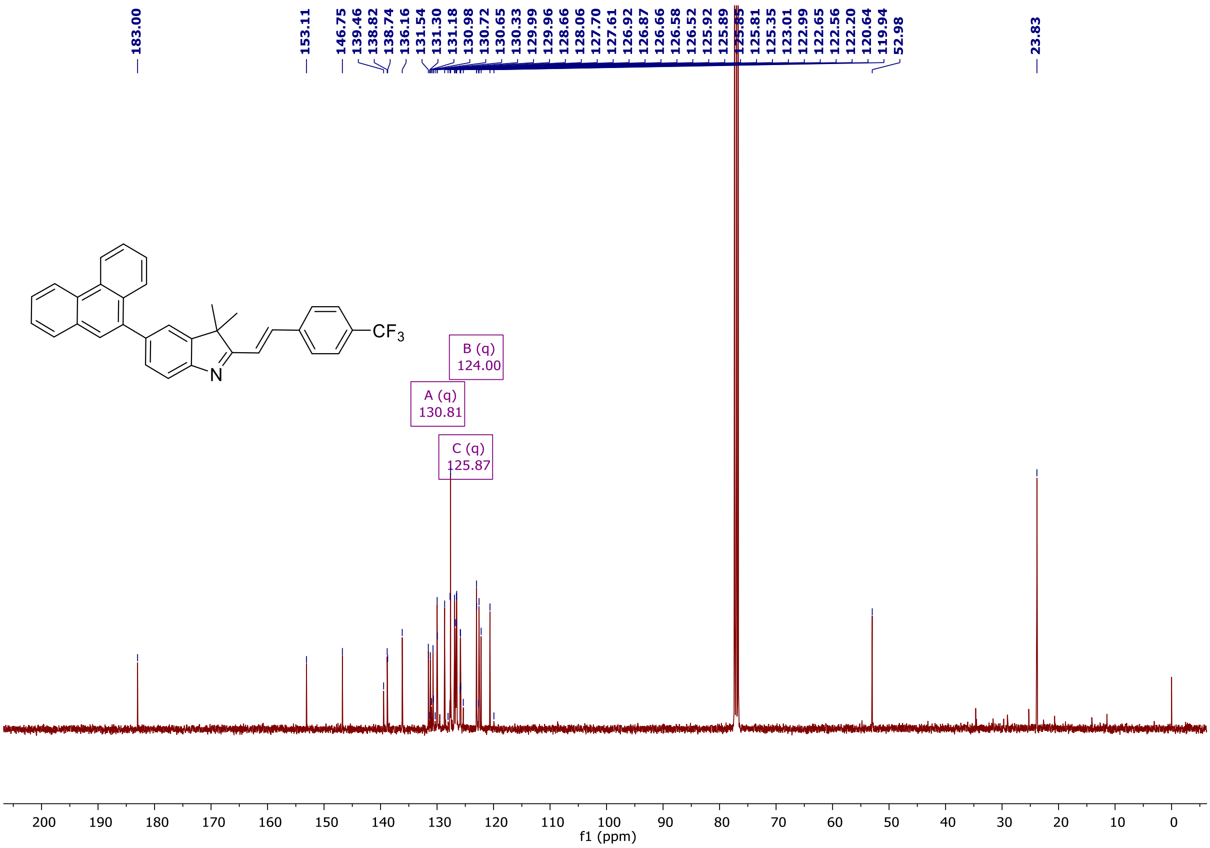

Figure S39. 3,3-Dimethyl-5-(phenanthren-9-yl)-2-{(*E*)-2-[4-(trifluoromethyl)phenyl]ethenyl}-3*H*-indole (**11**). ^{13}C NMR spectrum (101 MHz, $CDCl_3$).

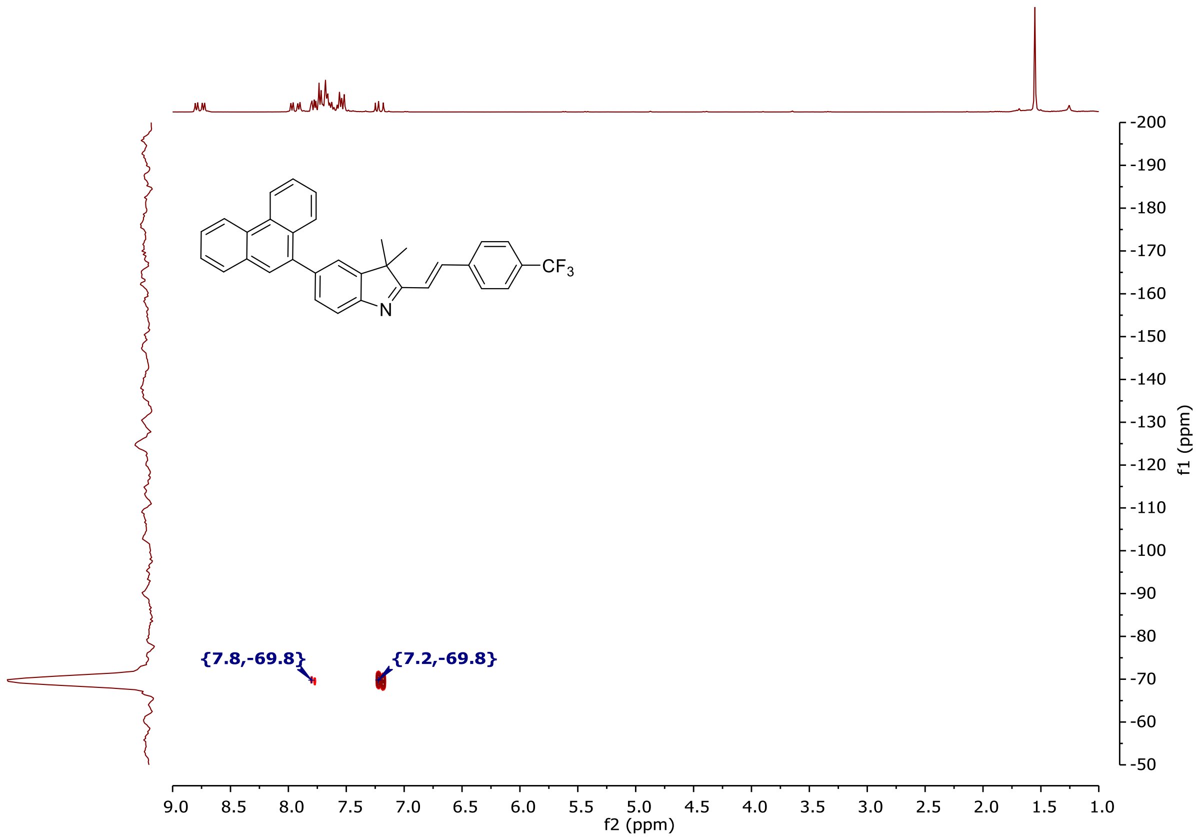

Figure S40. 3,3-Dimethyl-5-(phenanthren-9-yl)-2-{(*E*)-2-[4-(trifluoromethyl)phenyl]ethenyl}-3*H*-indole (**11**). ^{1}H-^{15}N HMBC NMR spectrum (41 MHz, $CDCl_3$).

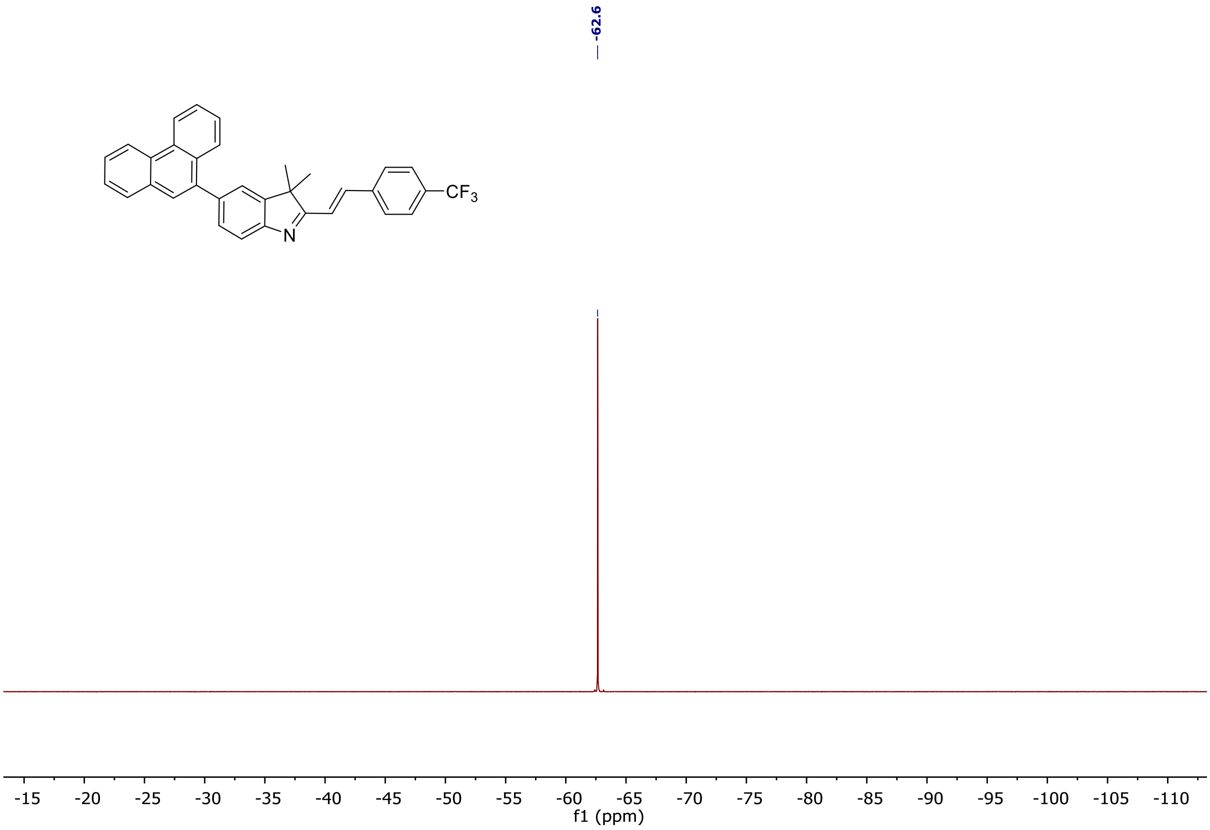

Figure S41. 3,3-Dimethyl-5-(phenanthren-9-yl)-2-{(*E*)-2-[4-(trifluoromethyl)phenyl]ethenyl}-3*H*-indole (**11**). ^{19}F NMR spectrum (376 MHz, $CDCl_3$).

Compound Spectrum SmartFormula Report

Analysis Info

Analysis Name	D:\Data\MRB-39_po_BMR.d	Acquisition Date	3/17/2025 5:35:50 PM
Method	DirectInfusion_TuneLow_pos.m	Operator	hplc
Sample Name	MRB-39_po_BMR	Instrument	micrOTOF-Q III 8228888.20448
Comment	AB		

Acquisition Parameter

Source Type	ESI	Ion Polarity	Positive	Set Nebulizer	0.4 Bar
Focus	Not active	Set Capillary	4500 V	Set Dry Heater	180 °C
Scan Begin	50 m/z	Set End Plate Offset	-500 V	Set Dry Gas	4.0 l/min
Scan End	1000 m/z	Set Collision Cell RF	140.0 Vpp	Set Divert Valve	Waste

#	RT [min]	Area	Int. Type	I	S/N	Chromatogram	Max. m/z	FWHM [min]
n.a.	7.1	n.a.	Single spectrum	n.a.	n.a.	n.a.	492.1936	n.a.

+MS, 7.1min #427

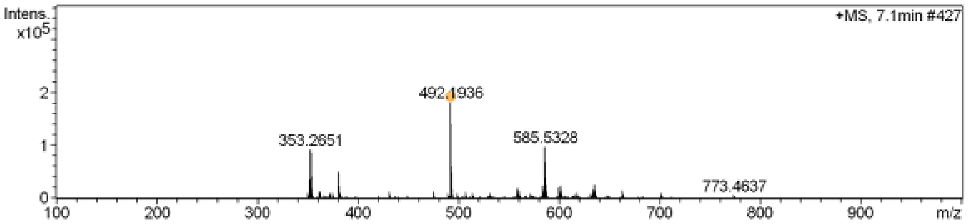

Meas. m/z	#	Ion Formula	m/z	err [ppm]	mSigma	# Sigma	Score	rdb	e⁻ Conf	N-Rule
492.1936	1	C33H25F3N	492.1934	-0.5	11.2	1	100.00	20.5	even	ok

Figure S42. 3,3-Dimethyl-5-(phenanthren-9-yl)-2-{(*E*)-2-[4-(trifluoromethyl)phenyl]ethenyl}-3*H*-indole (**11**). HRMS (ESI-TOF).

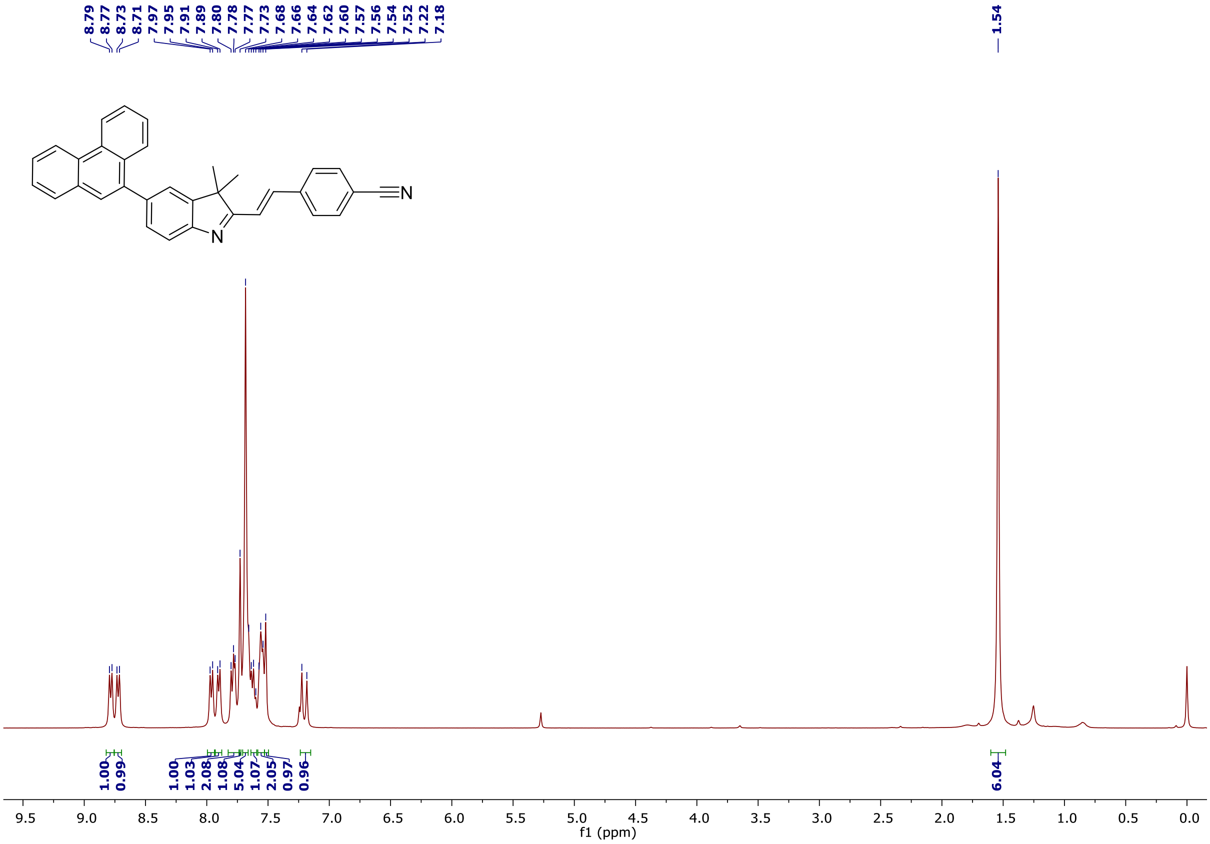

Figure S43. 4-{(*E*)-2-[3,3-Dimethyl-5-(phenanthren-9-yl)-3*H*-indol-2-yl]ethenyl}benzonitrile (**12**). ^{1}H NMR spectrum (400 MHz, $CDCl_3$).

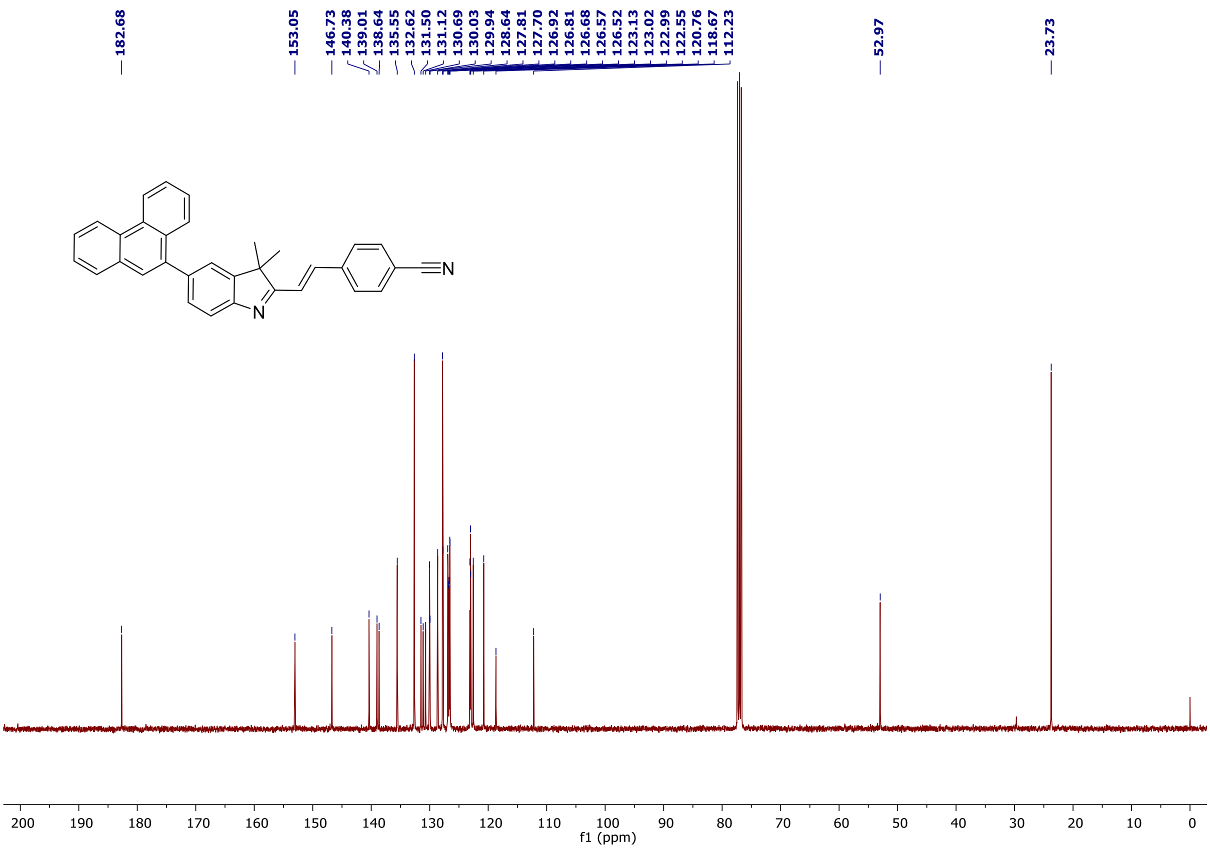

Figure S44. 4-{(*E*)-2-[3,3-Dimethyl-5-(phenanthren-9-yl)-3*H*-indol-2-yl]ethenyl}benzonitrile (**12**). ^{13}C NMR spectrum (101 MHz, $CDCl_3$).

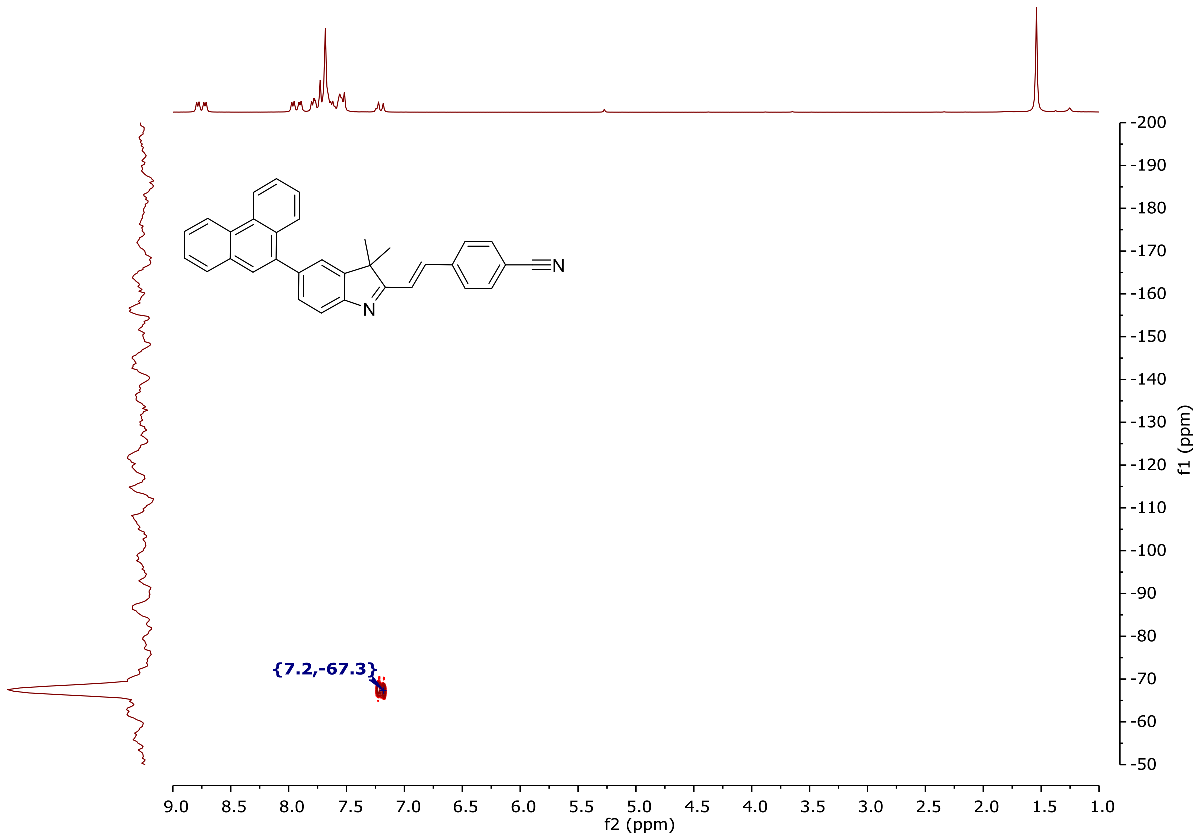

Figure S45. 4-{(*E*)-2-[3,3-Dimethyl-5-(phenanthren-9-yl)-3*H*-indol-2-yl]ethenyl}benzonitrile (**12**). ^{1}H-^{15}N HMBC NMR spectrum (41 MHz, CDCl$_3$).

Compound Spectrum SmartFormula Report

Analysis Info

Analysis Name	D:\Data\MRB-43.d	Acquisition Date	3/13/2025 9:58:30 AM
Method	DirectInfusion_TuneLow_pos.m	Operator	hplc
Sample Name	MRB-43	Instrument	micrOTOF-Q III 8228888.20448
Comment	SB		

Acquisition Parameter

Source Type	ESI	Ion Polarity	Positive	Set Nebulizer	0.4 Bar
Focus	Not active	Set Capillary	4500 V	Set Dry Heater	180 °C
Scan Begin	50 m/z	Set End Plate Offset	-500 V	Set Dry Gas	4.0 l/min
Scan End	1000 m/z	Set Collision Cell RF	140.0 Vpp	Set Divert Valve	Waste

#	RT [min]	Area	Int. Type	I	S/N	Chromatogram	Max. m/z	FWHM [min]
n.a.	1.3	n.a.	Single spectrum	n.a.	n.a.	n.a.	226.9528	n.a.
n.a.	4.7	n.a.	Single spectrum	n.a.	n.a.	n.a.	449.2012	n.a.

+MS, 4.7min #279

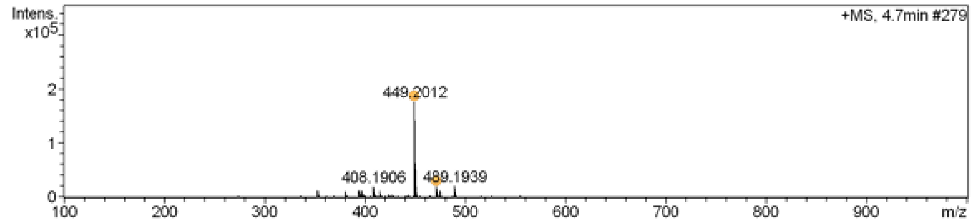

Meas. m/z	#	Ion Formula	m/z	err [ppm]	mSigma	# Sigma	Score	rdb	e⁻ Conf	N-Rule
449.2012	1	C33H25N2	449.2012	-0.2	1.6	1	100.00	22.5	even	ok
471.1837	1	C33H24N2Na	471.1832	1.1	11.6	1	100.00	22.5	even	ok

Figure S46. 4-{(*E*)-2-[3,3-Dimethyl-5-(phenanthren-9-yl)-3*H*-indol-2-yl]ethenyl}benzonitrile (**12**). HRMS (ESI-TOF).

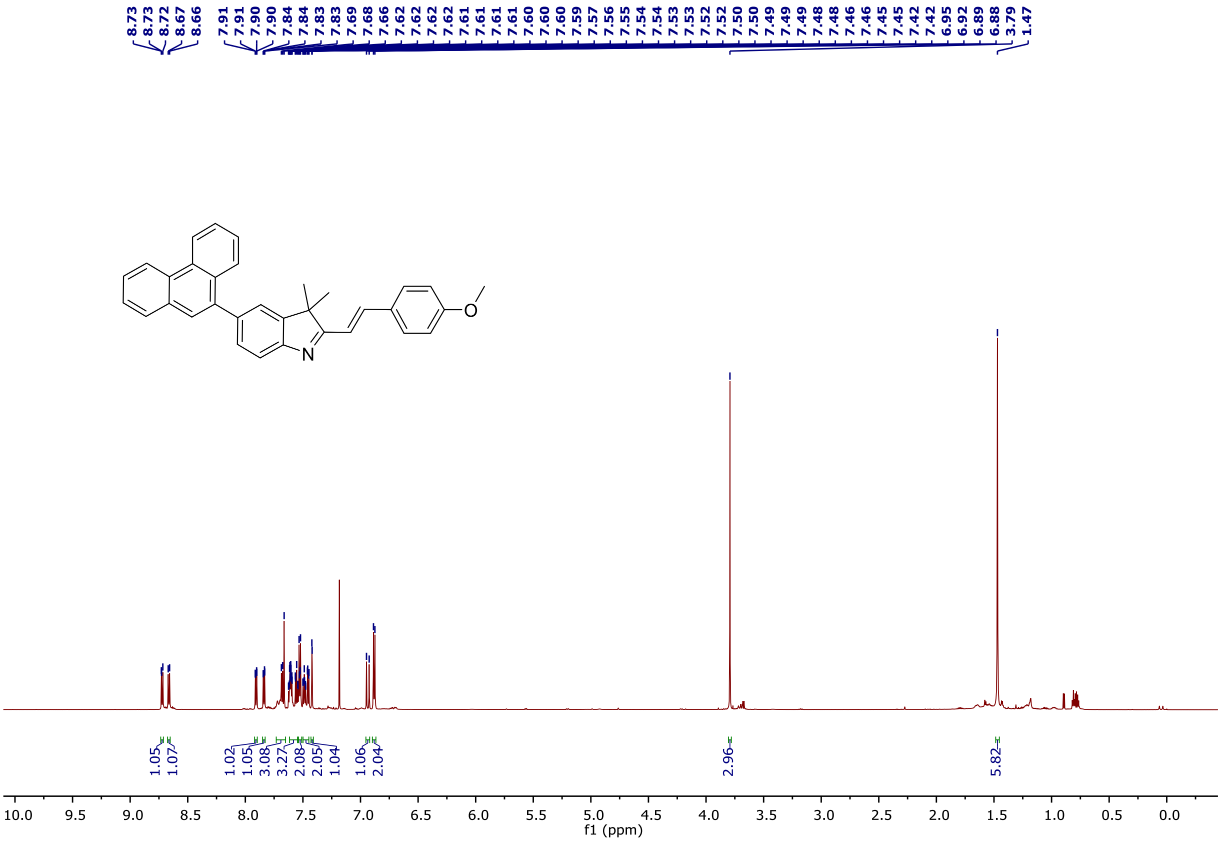

Figure S47. 2-[(*E*)-2-(4-Methoxyphenyl)ethenyl]-3,3-dimethyl-5-(phenanthren-9-yl)-3*H*-indole (**13**). ^{1}H NMR spectrum (700 MHz, $CDCl_3$).

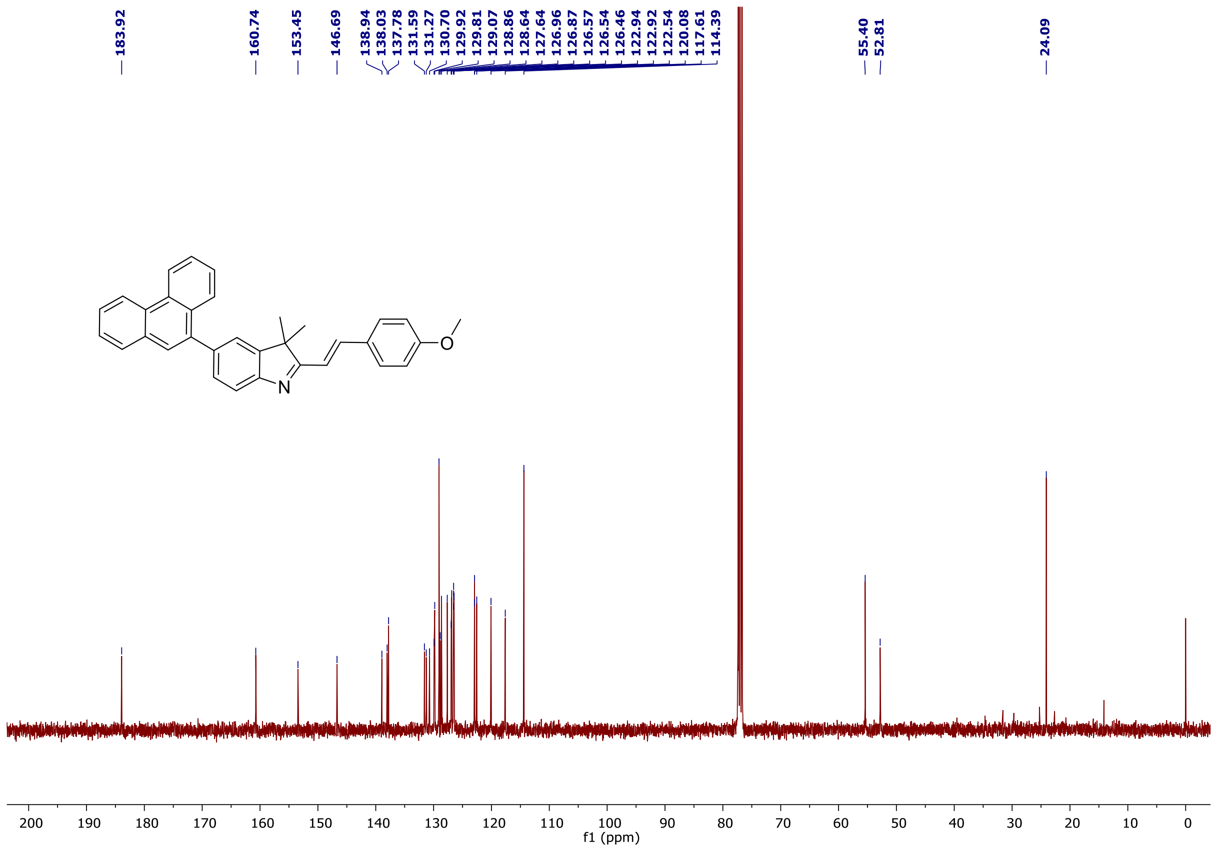

Figure S48. 2-[(*E*)-2-(4-Methoxyphenyl)ethenyl]-3,3-dimethyl-5-(phenanthren-9-yl)-3*H*-indole (**13**). ^{13}C NMR spectrum (101 MHz, $CDCl_3$).

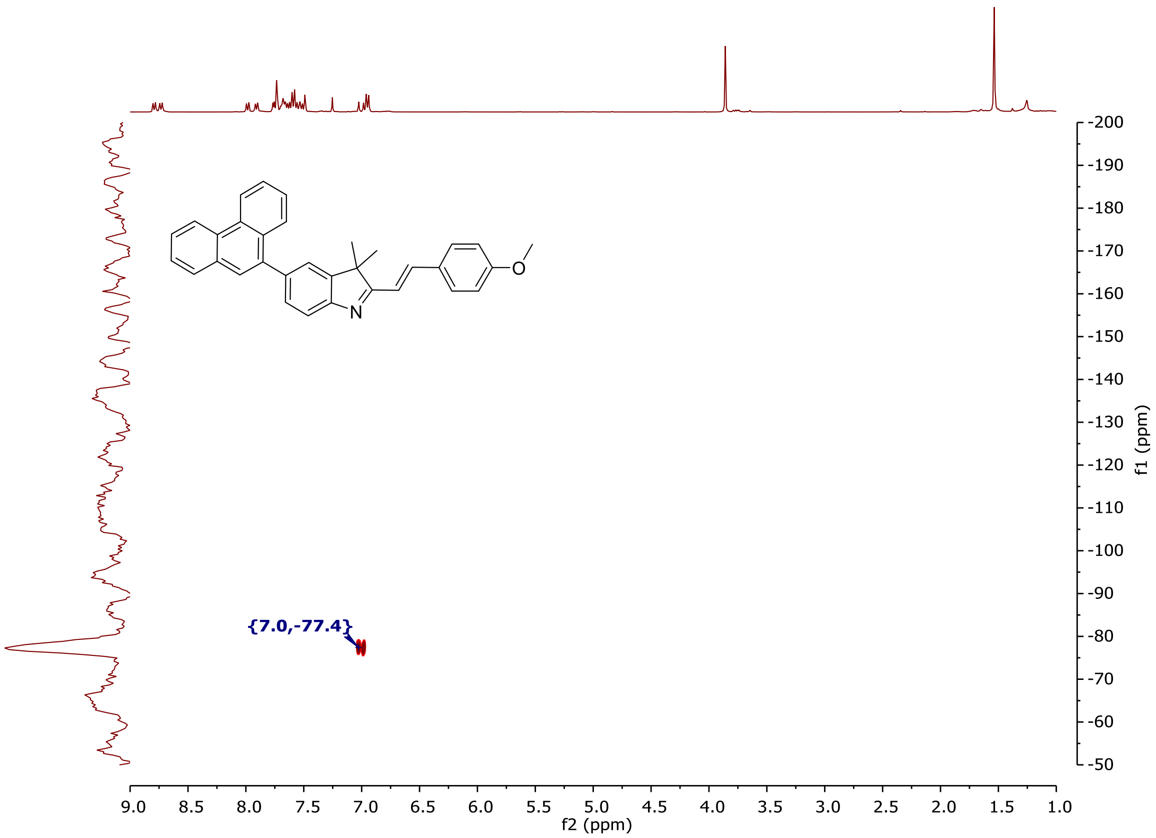

Figure S49. 2-[(*E*)-2-(4-Methoxyphenyl)ethenyl]-3,3-dimethyl-5-(phenanthren-9-yl)-3*H*-indole (**13**). ^{1}H-^{15}N HMBC NMR spectrum (41 MHz, $CDCl_3$).

Compound Spectrum SmartFormula Report

Analysis Info

Analysis Name	D:\Data\MRB-46.d	Acquisition Date	3/13/2025 10:13:29 AM
Method	DirectInfusion_TuneLow_pos.m	Operator	hplc
Sample Name	MRB-46	Instrument	micrOTOF-Q III 8228888.20448
Comment	SB		

Acquisition Parameter

Source Type	ESI	Ion Polarity	Positive	Set Nebulizer	0.4 Bar
Focus	Not active	Set Capillary	4500 V	Set Dry Heater	180 °C
Scan Begin	50 m/z	Set End Plate Offset	-500 V	Set Dry Gas	4.0 l/min
Scan End	1000 m/z	Set Collision Cell RF	140.0 Vpp	Set Divert Valve	Waste

#	RT [min]	Area	Int. Type	I	S/N	Chromatogram	Max. m/z	FWHM [min]
n.a.	0.5	n.a.	Single spectrum	n.a.	n.a.	n.a.	226.9527	n.a.
n.a.	3.0	n.a.	Single spectrum	n.a.	n.a.	n.a.	454.2165	n.a.

+MS, 3.0min #178

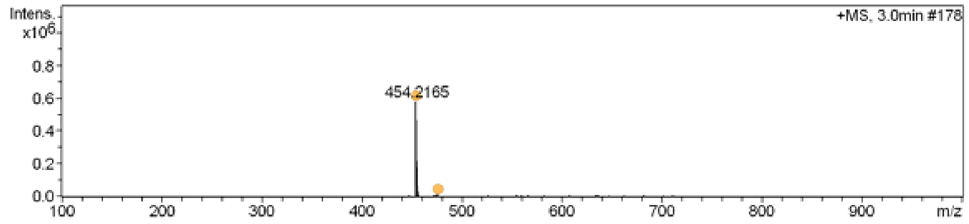

Meas. m/z	#	Ion Formula	m/z	err [ppm]	mSigma	# Sigma	Score	rdb	e⁻ Conf	N-Rule
454.2165	1	C33H28NO	454.2165	0.0	9.9	1	100.00	20.5	even	ok
476.1980	1	C33H27NNaO	476.1985	-1.1	26.9	1	100.00	20.5	even	ok

Figure S50. 2-[(*E*)-2-(4-Methoxyphenyl)ethenyl]-3,3-dimethyl-5-(phenanthren-9-yl)-3*H*-indole (**13**). HRMS (ESI-TOF).

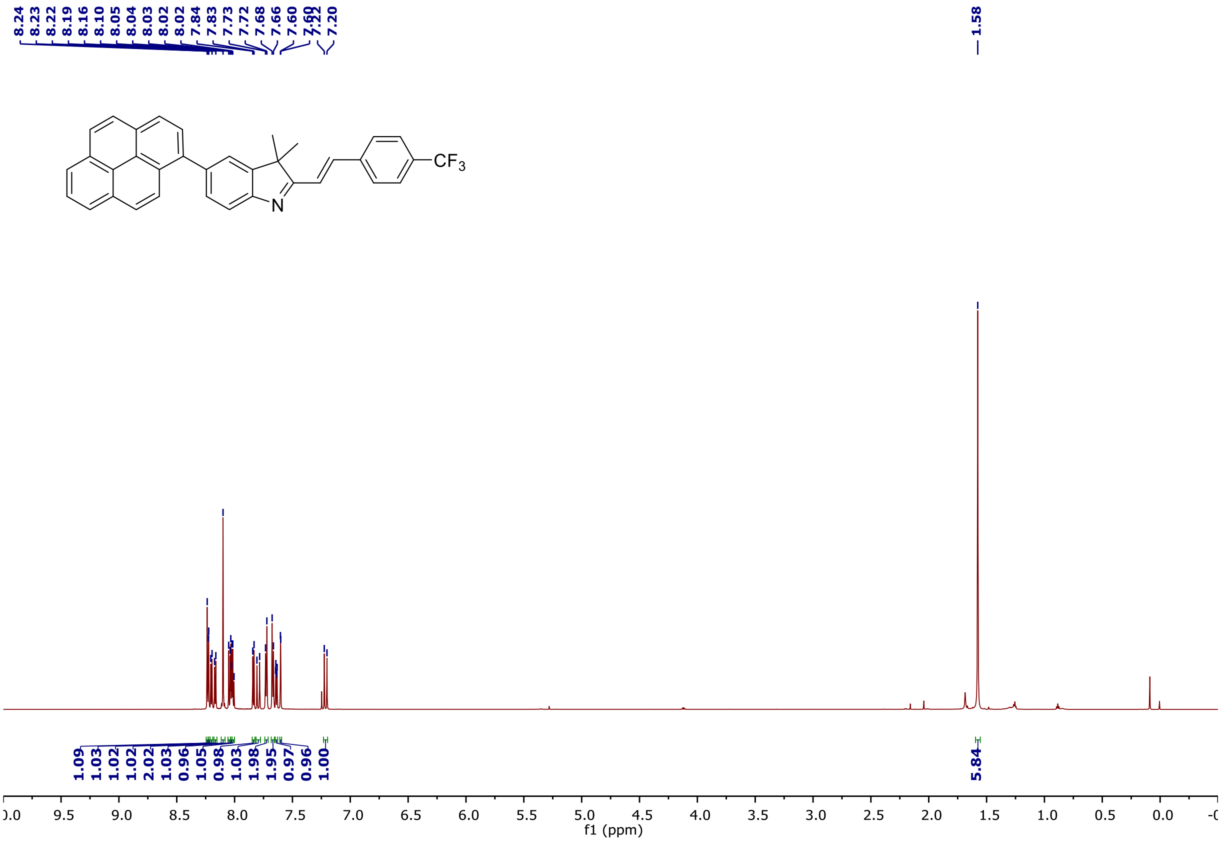

Figure S51. 3,3-Dimethyl-5-(pyren-1-yl)-2-{(*E*)-2-[4-(trifluoromethyl)phenyl]ethenyl}-3*H*-indole (**14**). ^{1}H NMR spectrum (700 MHz, $CDCl_3$).

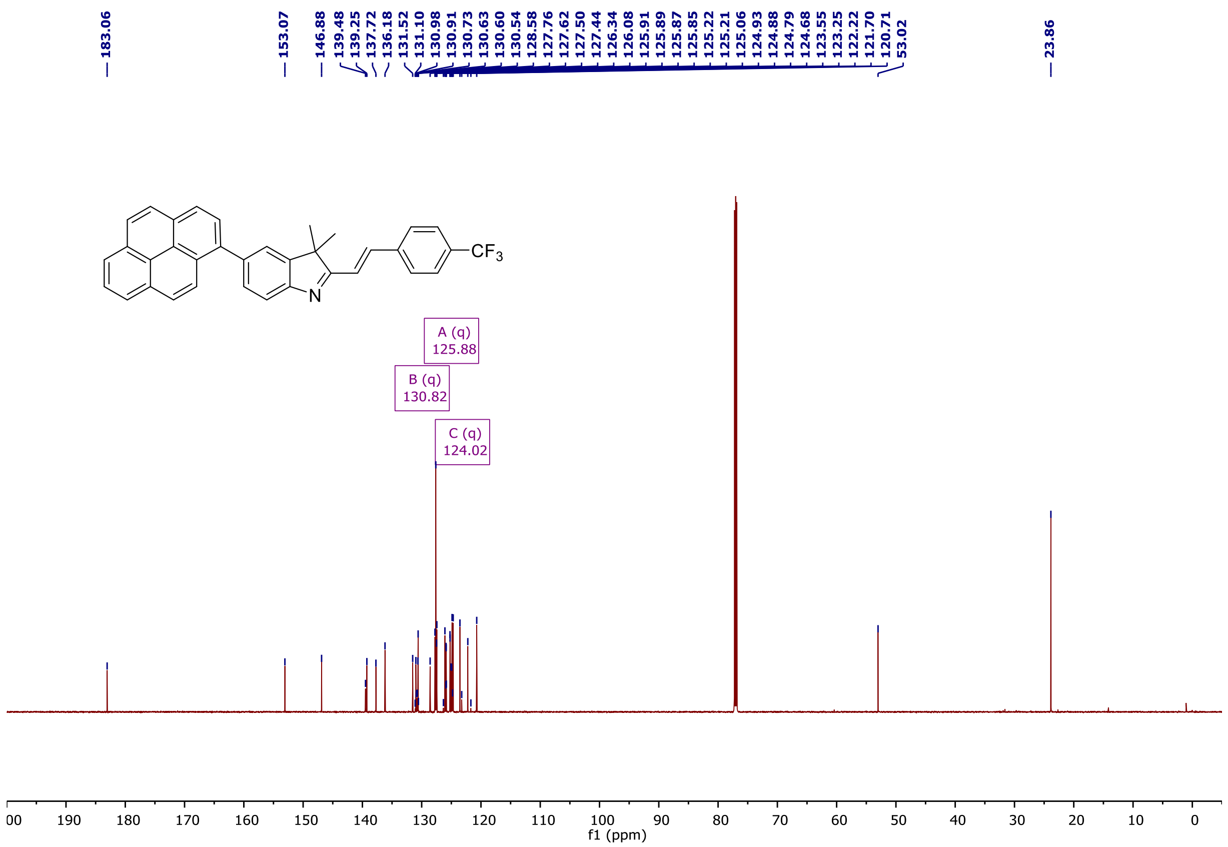

Figure S52. 3,3-Dimethyl-5-(pyren-1-yl)-2-{(*E*)-2-[4-(trifluoromethyl)phenyl]ethenyl}-3*H*-indole (**14**). ^{13}C NMR spectrum (176 MHz, $CDCl_3$).

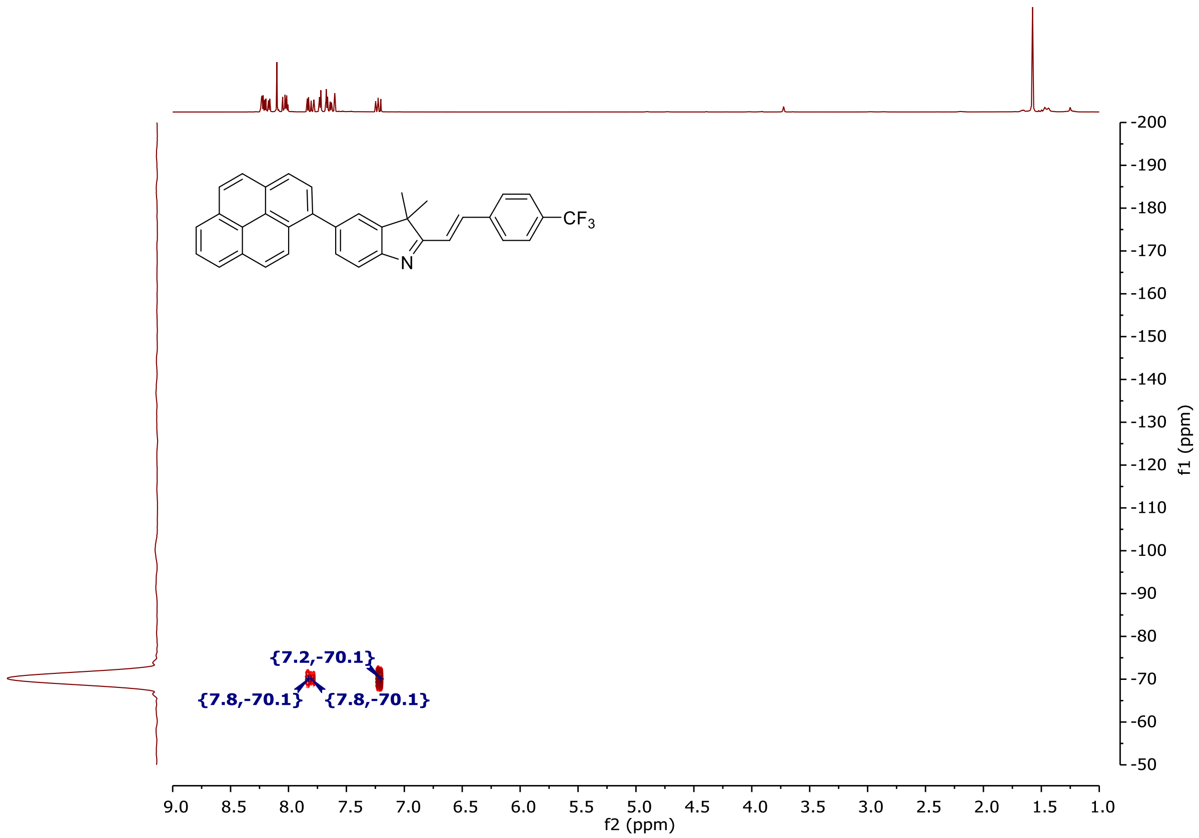

Figure S53. 3,3-Dimethyl-5-(pyren-1-yl)-2-{(*E*)-2-[4-(trifluoromethyl)phenyl]ethenyl}-3*H*-indole (**14**). ^{1}H-^{15}N HMBC NMR spectrum (71 MHz, $CDCl_3$).

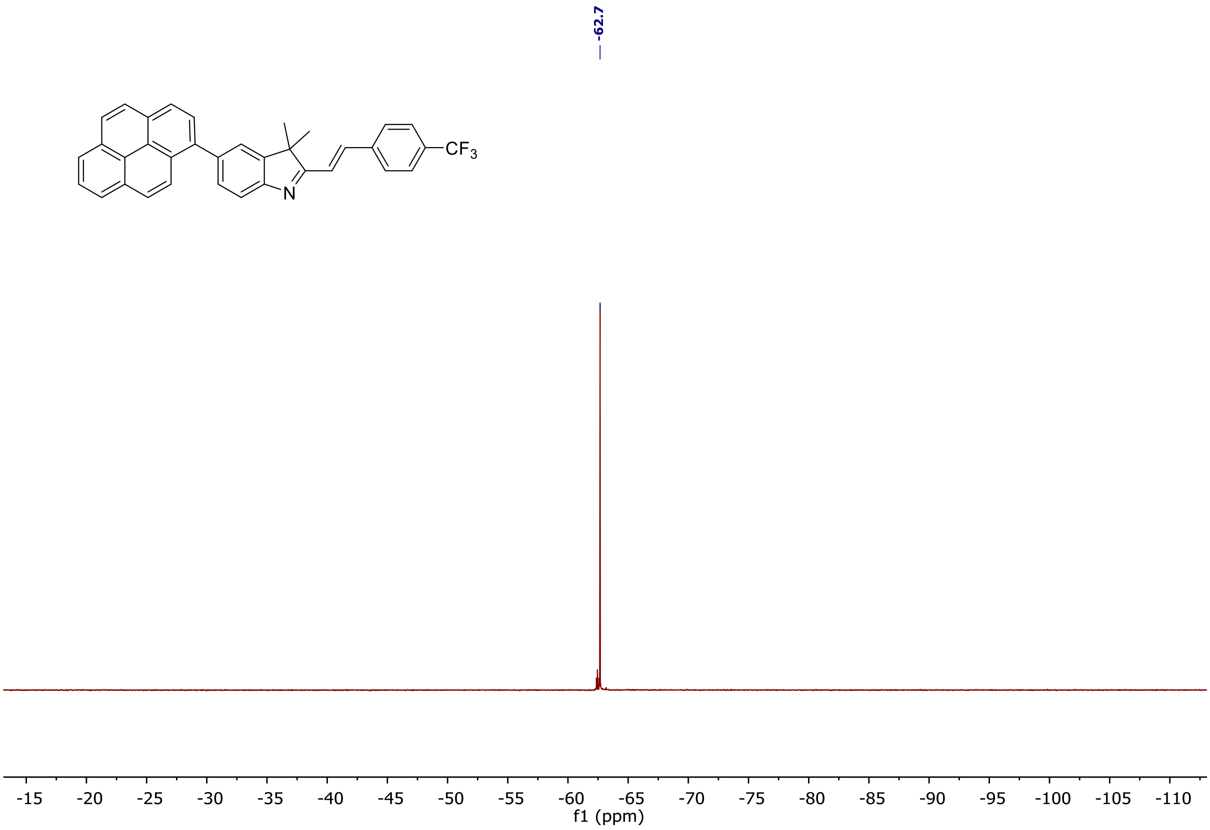

Figure S54. 3,3-Dimethyl-5-(pyren-1-yl)-2-{(*E*)-2-[4-(trifluoromethyl)phenyl]ethenyl}-3*H*-indole (**14**). ^{19}F NMR spectrum (376 MHz, $CDCl_3$).

Compound Spectrum SmartFormula Report

Analysis Info

Analysis Name	D:\Data\MRB-34.d	Acquisition Date	3/13/2025 1:25:21 PM
Method	DirectInfusion_TuneLow_pos.m	Operator	hplc
Sample Name	MRB-34	Instrument	micrOTOF-Q III 8228888.20448
Comment	SB		

Acquisition Parameter

Source Type	ESI	Ion Polarity	Positive	Set Nebulizer	0.4 Bar
Focus	Not active	Set Capillary	4500 V	Set Dry Heater	180 °C
Scan Begin	50 m/z	Set End Plate Offset	-500 V	Set Dry Gas	4.0 l/min
Scan End	1000 m/z	Set Collision Cell RF	140.0 Vpp	Set Divert Valve	Waste

#	RT [min]	Area	Int. Type	I	S/N	Chromatogram	Max. m/z	FWHM [min]
n.a.	0.1	n.a.	Single spectrum	n.a.	n.a.	n.a.	226.9528	n.a.
n.a.	4.6	n.a.	Single spectrum	n.a.	n.a.	n.a.	516.1934	n.a.

+MS, 4.6min #273

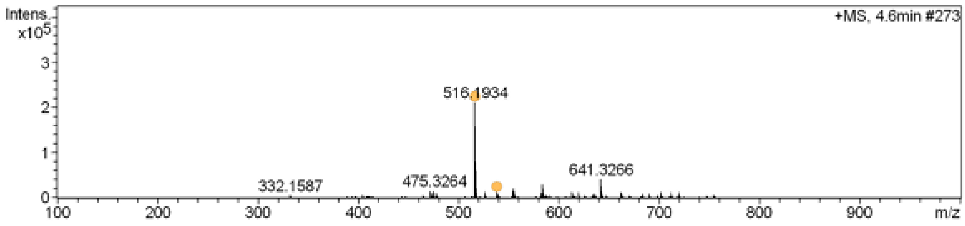

Meas. m/z	#	Ion Formula	m/z	err [ppm]	mSigma	# Sigma	Score	rdb	e⁻ Conf	N-Rule
516.1934	1	C35H25F3N	516.1934	0.0	2.7	1	100.00	22.5	even	ok
538.1747	1	C35H24F3NNa	538.1753	-1.2	48.2	1	100.00	22.5	even	ok

Bruker Compass DataAnalysis 4.1 printed: 3/13/2025 1:36:26 PM by: hplc Page 1 of 1

Figure S55. 3,3-Dimethyl-5-(pyren-1-yl)-2-{(*E*)-2-[4-(trifluoromethyl)phenyl]ethenyl}-3*H*-indole (**14**). HRMS (ESI-TOF).

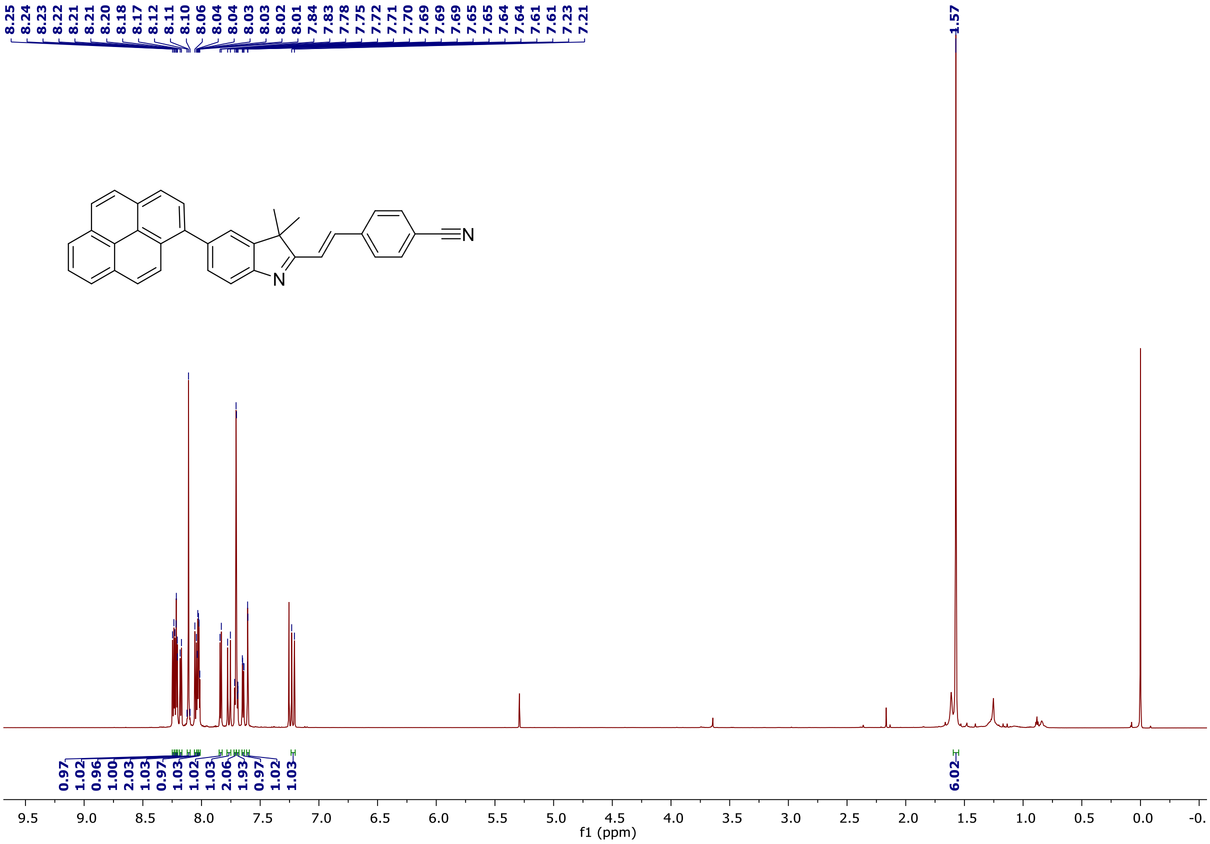

Figure S56. 4-{(*E*)-2-[3,3-Dimethyl-5-(pyren-1-yl)-3*H*-indol-2-yl]ethenyl}benzonitrile (**15**). ^{1}H NMR spectrum (700 MHz, $CDCl_3$).

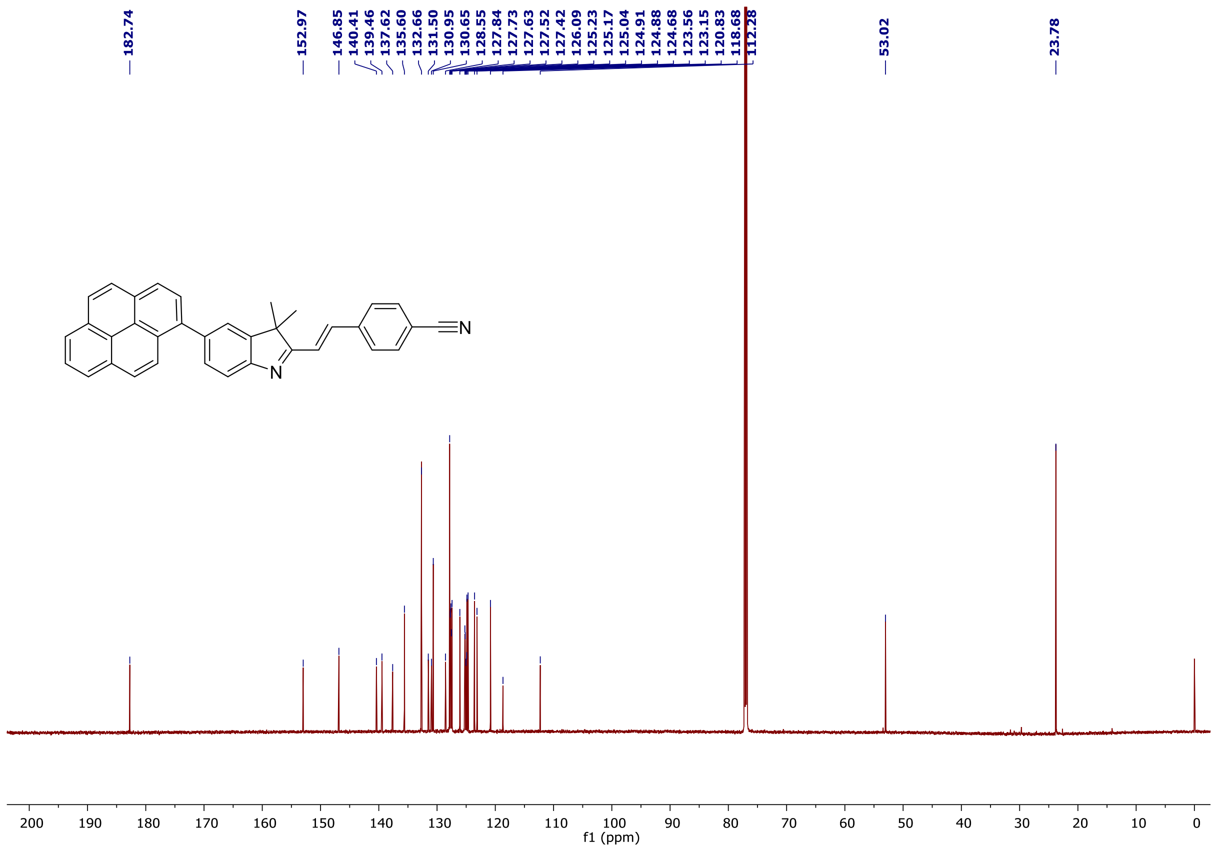

Figure S57. 4-{(*E*)-2-[3,3-Dimethyl-5-(pyren-1-yl)-3*H*-indol-2-yl]ethenyl}benzonitrile (**15**). ^{13}C NMR spectrum (176 MHz, $CDCl_3$).

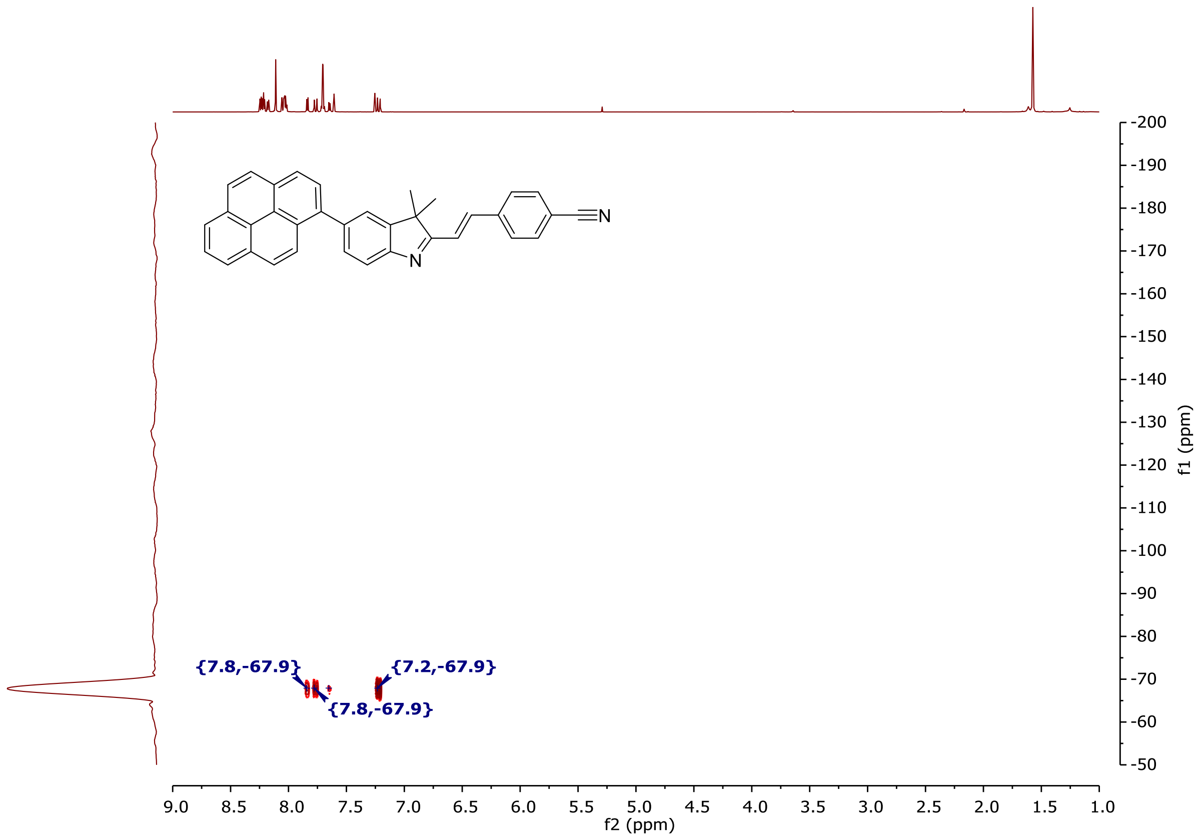

Figure S58. 4-{(*E*)-2-[3,3-Dimethyl-5-(pyren-1-yl)-3*H*-indol-2-yl]ethenyl}benzonitrile (**15**). ^{1}H-^{15}N HMBC NMR spectrum (71 MHz, $CDCl_3$).

Compound Spectrum SmartFormula Report

Analysis Info

Analysis Name	D:\Data\MRB-52.d	Acquisition Date	3/13/2025 10:26:12 AM
Method	DirectInfusion_TuneLow_pos.m	Operator	hplc
Sample Name	MRB-52	Instrument	micrOTOF-Q III 8228888.20448
Comment	SB		

Acquisition Parameter

Source Type	ESI	Ion Polarity	Positive	Set Nebulizer	0.4 Bar
Focus	Not active	Set Capillary	4500 V	Set Dry Heater	180 °C
Scan Begin	50 m/z	Set End Plate Offset	-500 V	Set Dry Gas	4.0 l/min
Scan End	1000 m/z	Set Collision Cell RF	140.0 Vpp	Set Divert Valve	Waste

#	RT [min]	Area	Int. Type	I	S/N	Chromatogram	Max. m/z	FWHM [min]
n.a.	0.1	n.a.	Single spectrum	n.a.	n.a.	n.a.	226.9524	n.a.
n.a.	8.0	n.a.	Single spectrum	n.a.	n.a.	n.a.	473.2012	n.a.

+MS, 8.0min #478

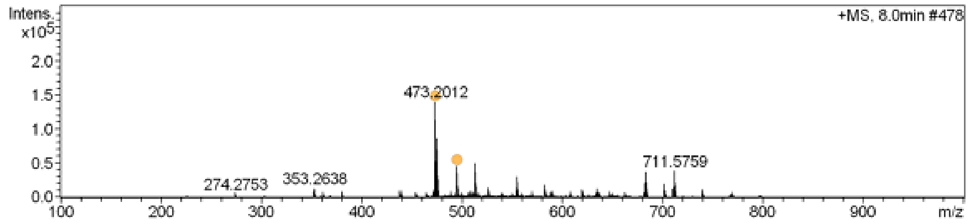

Meas. m/z	#	Ion Formula	m/z	err [ppm]	mSigma	# Sigma	Score	rdb	e⁻ Conf	N-Rule
473.2012	1	C35H25N2	473.2012	-0.0	15.4	1	100.00	24.5	even	ok
495.1823	1	C35H24N2Na	495.1832	-1.8	22.6	1	100.00	24.5	even	ok

Figure S59. 4-{(*E*)-2-[3,3-Dimethyl-5-(pyren-1-yl)-3*H*-indol-2-yl]ethenyl}benzonitrile (**15**). HRMS (ESI-TOF).

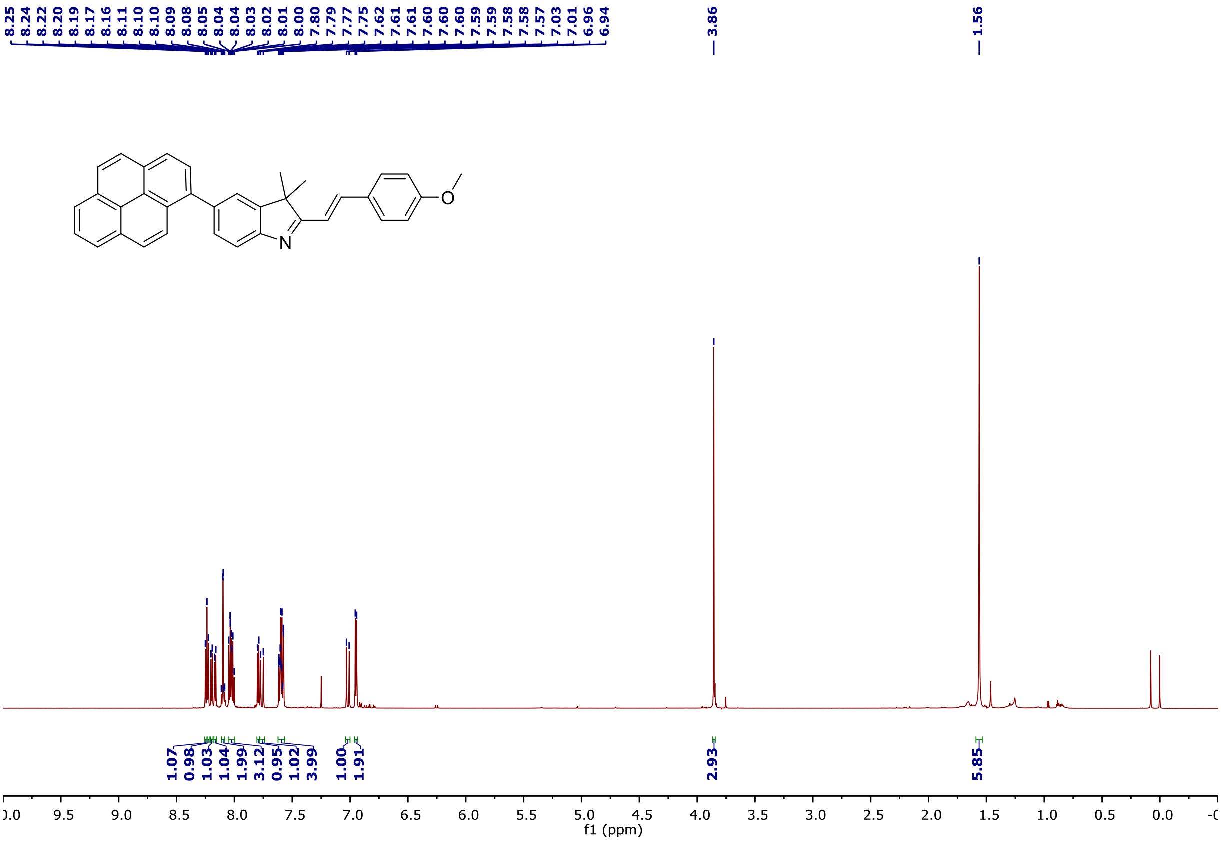

Figure S60. 2-[(*E*)-2-(4-Methoxyphenyl)ethenyl]-3,3-dimethyl-5-(pyren-1-yl)-3*H*-indole (**16**). ^{1}H NMR spectrum (700 MHz, $CDCl_3$).

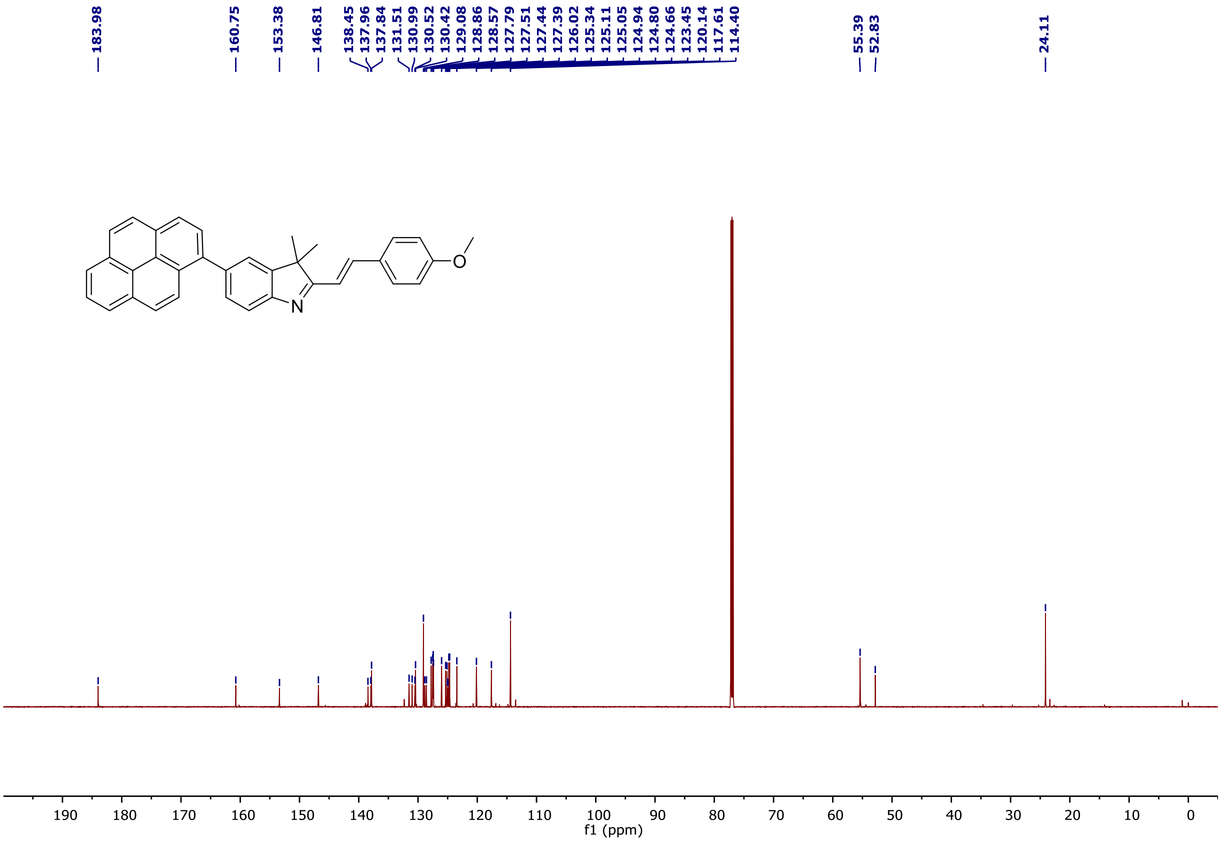

Figure S61. 2-[(*E*)-2-(4-Methoxyphenyl)ethenyl]-3,3-dimethyl-5-(pyren-1-yl)-3*H*-indole (**16**). ^{13}C NMR spectrum (176 MHz, $CDCl_3$).

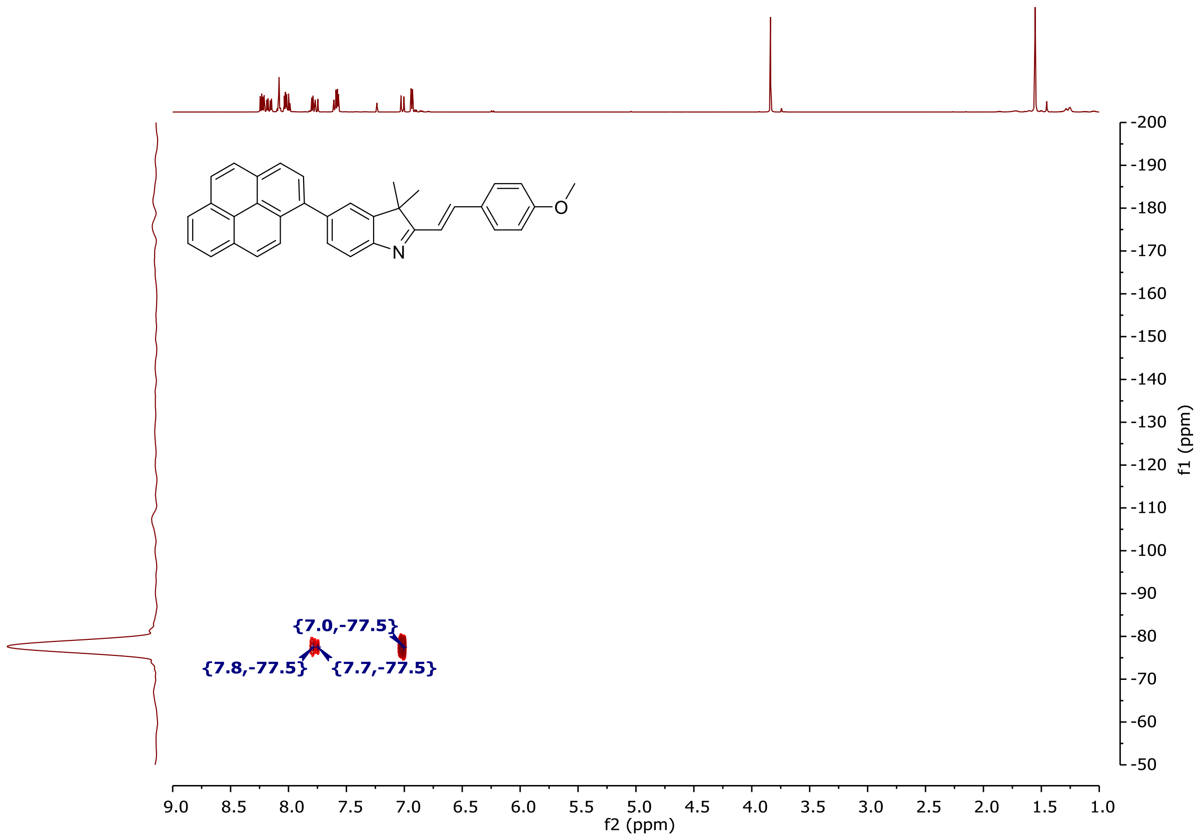

Figure S62. 2-[(*E*)-2-(4-Methoxyphenyl)ethenyl]-3,3-dimethyl-5-(pyren-1-yl)-3*H*-indole (**16**). ^{1}H-^{15}N HMBC NMR spectrum (71 MHz, $CDCl_3$).

Compound Spectrum SmartFormula Report

Analysis Info

Analysis Name	D:\Data\MRB-67.d	Acquisition Date	3/13/2025 11:01:14 AM
Method	DirectInfusion_TuneLow_pos.m	Operator	hplc
Sample Name	MRB-67	Instrument	micrOTOF-Q III 8228888.20448
Comment	SB		

Acquisition Parameter

Source Type	ESI	Ion Polarity	Positive	Set Nebulizer	0.4 Bar
Focus	Not active	Set Capillary	4500 V	Set Dry Heater	180 °C
Scan Begin	50 m/z	Set End Plate Offset	-500 V	Set Dry Gas	4.0 l/min
Scan End	1000 m/z	Set Collision Cell RF	140.0 Vpp	Set Divert Valve	Waste

#	RT [min]	Area	Int. Type	I	S/N	Chromatogram	Max. m/z	FWHM [min]
n.a.	1.4	n.a.	Single spectrum	n.a.	n.a.	n.a.	226.9528	n.a.
n.a.	2.2	n.a.	Single spectrum	n.a.	n.a.	n.a.	478.2165	n.a.

+MS, 2.2min #133

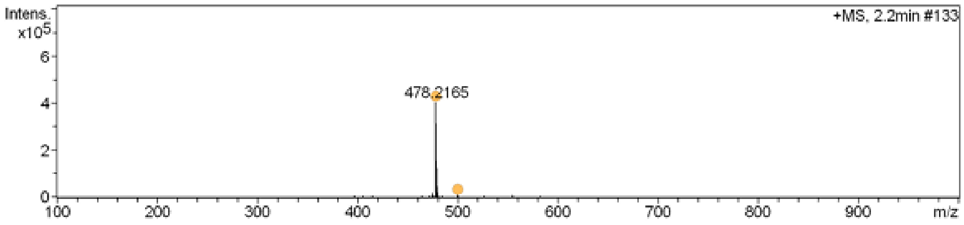

Meas. m/z	#	Ion Formula	m/z	err [ppm]	mSigma	# Sigma	Score	rdb	e⁻ Conf	N-Rule
478.2165	1	C35H28NO	478.2165	0.2	1.6	1	100.00	22.5	even	ok
500.1987	1	C35H27NNaO	500.1985	0.4	69.8	1	100.00	22.5	even	ok

Figure S63. 2-[(*E*)-2-(4-Methoxyphenyl)ethenyl]-3,3-dimethyl-5-(pyren-1-yl)-3*H*-indole (**16**). HRMS (ESI-TOF).

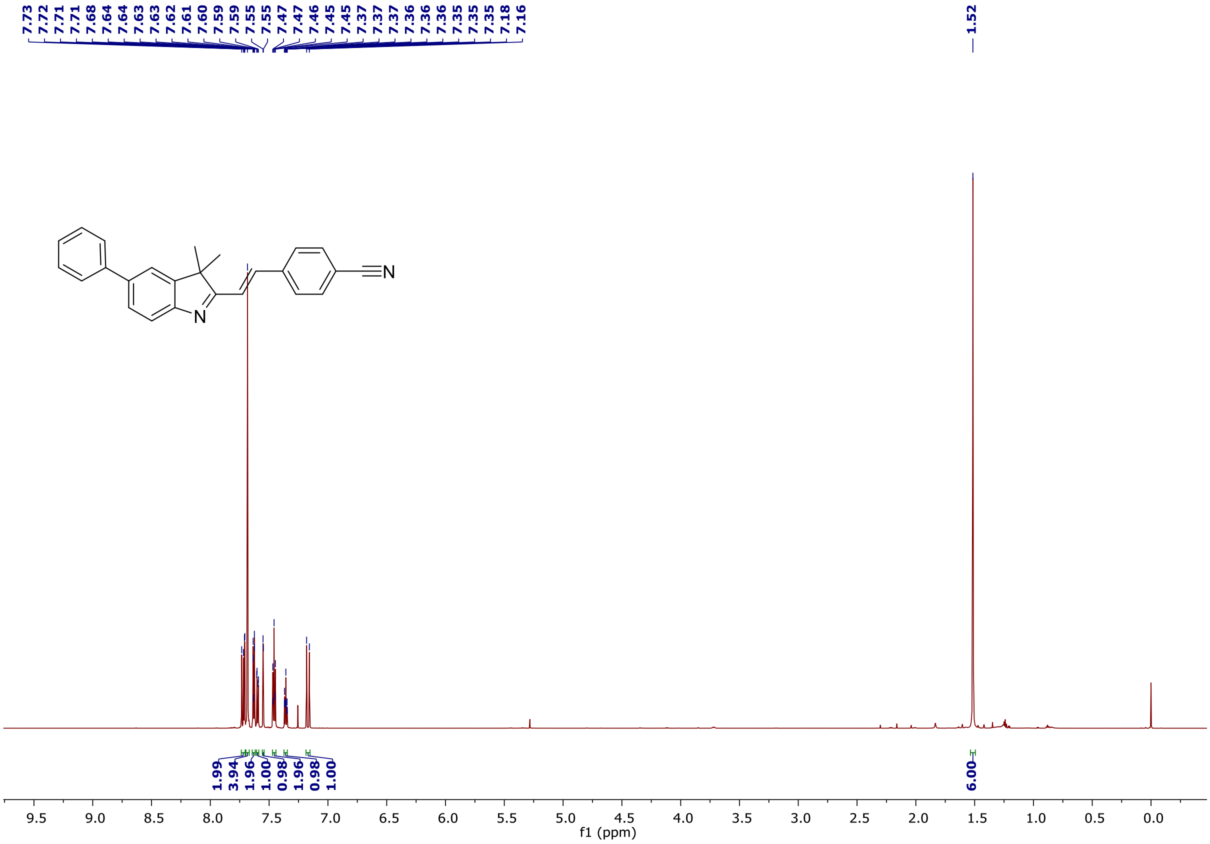

Figure S64. 4-{(*E*)-2-[3,3-Dimethyl-5-phenyl-3*H*-indol-2-yl]ethenyl}benzonitrile (**17**). ^{1}H NMR spectrum (700 MHz, $CDCl_3$).

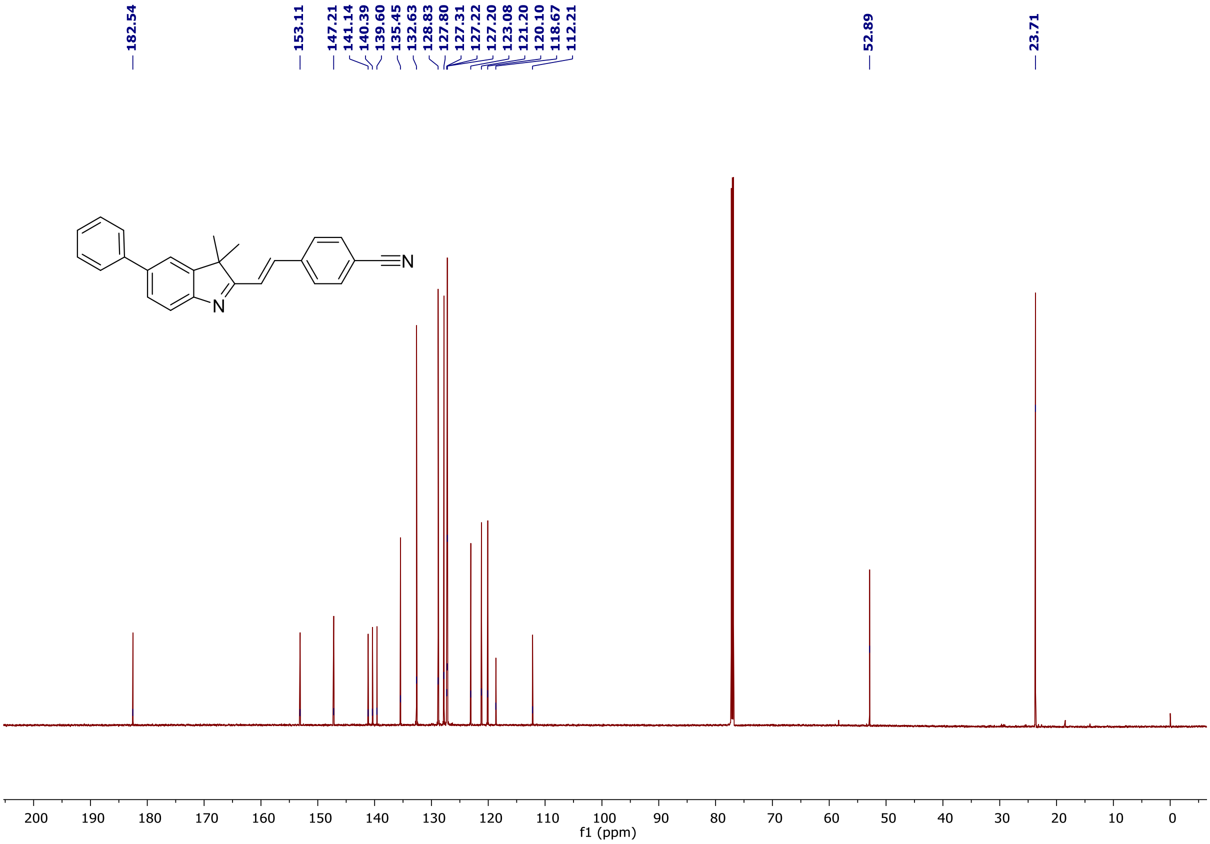

Figure S65. 4-{(*E*)-2-[3,3-Dimethyl-5-phenyl-3*H*-indol-2-yl]ethenyl}benzonitrile (**17**). ^{13}C NMR spectrum (176 MHz, $CDCl_3$).

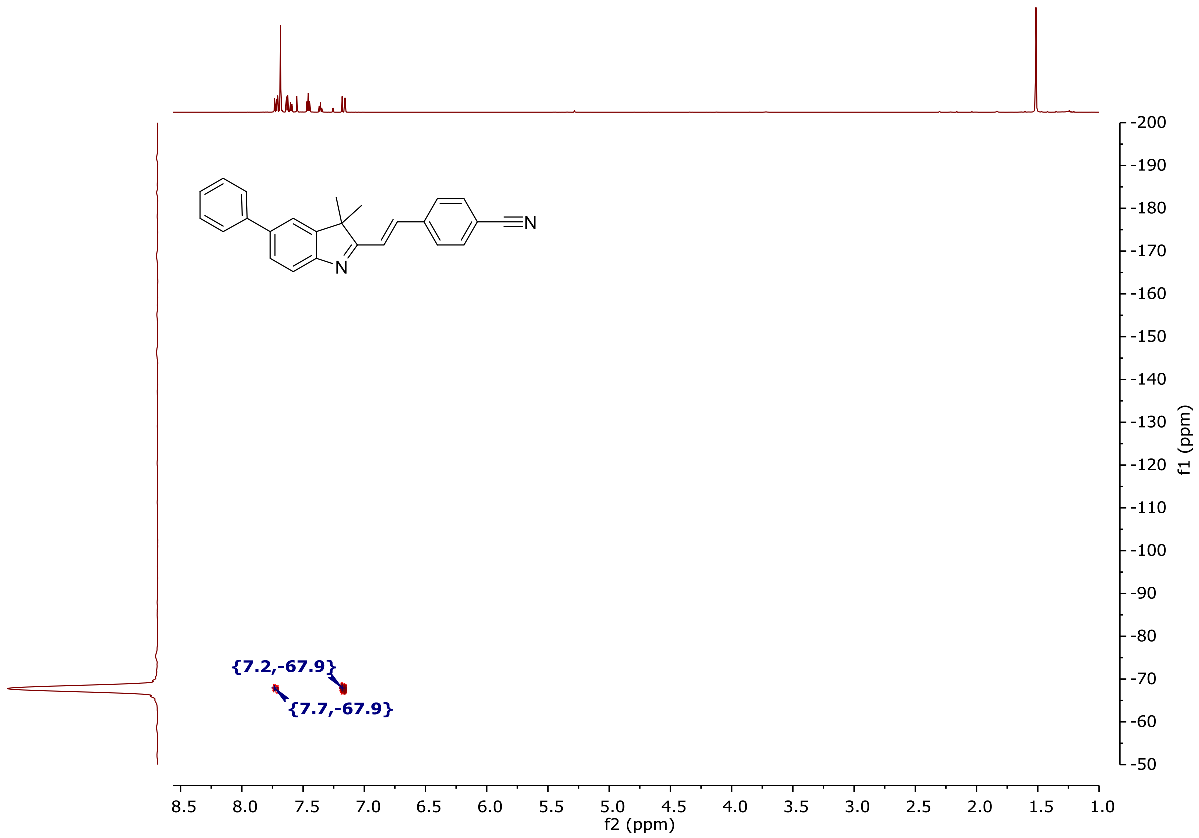

Figure S66. 4-{(*E*)-2-[3,3-Dimethyl-5-phenyl-3*H*-indol-2-yl]ethenyl}benzonitrile (**17**). ^{1}H-^{15}N HMBC NMR spectrum (71 MHz, $CDCl_3$).

Compound Spectrum SmartFormula Report

Analysis Info

Analysis Name	D:\Data\MRB-105.d	Acquisition Date	3/13/2025 11:13:28 AM
Method	DirectInfusion_TuneLow_pos.m	Operator	hplc
Sample Name	MRB-105	Instrument	micrOTOF-Q III 8228888.20448
Comment	SB		

Acquisition Parameter

Source Type	ESI	Ion Polarity	Positive	Set Nebulizer	0.4 Bar
Focus	Not active	Set Capillary	4500 V	Set Dry Heater	180 °C
Scan Begin	50 m/z	Set End Plate Offset	-500 V	Set Dry Gas	4.0 l/min
Scan End	1000 m/z	Set Collision Cell RF	140.0 Vpp	Set Divert Valve	Waste

#	RT [min]	Area	Int. Type	I	S/N	Chromatogram	Max. m/z	FWHM [min]
n.a.	0.1	n.a.	Single spectrum	n.a.	n.a.	n.a.	226.9528	n.a.
n.a.	3.6	n.a.	Single spectrum	n.a.	n.a.	n.a.	349.1699	n.a.

+MS, 3.6min #216

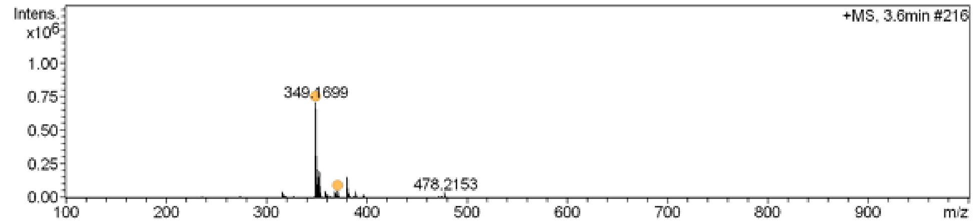

Meas. m/z	#	Ion Formula	m/z	err [ppm]	mSigma	# Sigma	Score	rdb	e⁻ Conf	N-Rule
349.1699	1	C25H21N2	349.1699	0.0	23.5	1	100.00	16.5	even	ok
371.1504	1	C25H20N2Na	371.1519	4.0	7.0	1	100.00	16.5	even	ok

Figure S67. 4-{(*E*)-2-[3,3-Dimethyl-5-phenyl-3*H*-indol-2-yl]ethenyl}benzonitrile (**17**). HRMS (ESI-TOF).
